# A New Class of Benzo[*b*]thiophene-chalcones as Cholinesterase Inhibitors: Synthesis, Biological Evaluation, Molecular Docking and ADME Studies

**DOI:** 10.3390/molecules29163748

**Published:** 2024-08-07

**Authors:** Giovanna Lucia Delogu, Michela Begala, Maria João Matos, Davide Crucitti, Valeria Sogos, Benedetta Era, Antonella Fais

**Affiliations:** 1 Department of Live and Environmental Sciences, University of Cagliari, Cittadella Universitaria, SS 554, Km 4.5, 09042 Monserrato, Italy; michelabegala@unica.it (M.B.); era@unica.it (B.E.); fais@unica.it (A.F.); 2 Departamento de Química Orgánica, Facultad de Farmacia, Universidade de Santiago de Compostela, 15782 Santiago de Compostela, Spain; 3 Group of Computational Genomics and Hematology (GreCoXen), Health Research Institute of Santiago de Compostela (IDIS), 15782 Santiago de Compostela, Spain; davide.crucitti@grheco.com; 4 Departamento de Farmacología, Farmacia y Tecnología Farmacéutica, Universidade de Santiago de Compostela, 15782 Santiago de Compostela, Spain; 5 Department of Biomediacal Science, University of Cagliari, Cittadella Universitaria, SS 554, Km 4.5, 09042 Monserrato, Italy; sogos@unica.it

**Keywords:** benzothiophenes, heterocycles, cholinesterase inhibitors

## Abstract

In this study, heterocyclic compounds containing a benzothiophene scaffold were designed and synthetized, and their inhibitory activity against cholinesterases (ChE) and the viability of SH-SY5Y cells have been evaluated. Benzothiophenes **4a**–**4i** and benzothiophene-chalcone hybrids **5a**–**5i** were tested against both acetylcholinesterase (AChE) and butyrylcholinesterase (BChE), revealing interesting structure–activity relationships. In general, benzothiophene–chalcone hybrids from series **5** proved to be better inhibitors of both enzymes, with compound **5f** being the best AChE inhibitor (IC_50_ = 62.10 μM) and compound **5h** being the best BChE inhibitor (IC_50_ = 24.35 μM), the last one having an IC_50_ similar to that of galantamine (IC_50_ = 28.08 μM), the reference compound. The in silico ADME profile of the compounds was also studied. Molecular docking calculations were carried out to analyze the best binding scores and to elucidate enzyme–inhibitors’ interactions.

## 1. Introduction

Medicinal chemists have successfully explored the versatile synthesis and biological applications of heterocyclic compounds to discover novel drugs with high efficacy and potency [1]. Heterocyclic compounds are extensively found in nature, and a significant number of drugs used in clinics contain a heterocyclic structure [2,3]. In the design of new drugs, specific heterocyclic fragments may enhance particular physicochemical properties [4]. By incorporating specific substitutions at strategic positions, heterocyclic ring systems have emerged as potent scaffolds for biological assessment, making them indispensable in pursuing scientific breakthroughs [5].

**Figure 1:**
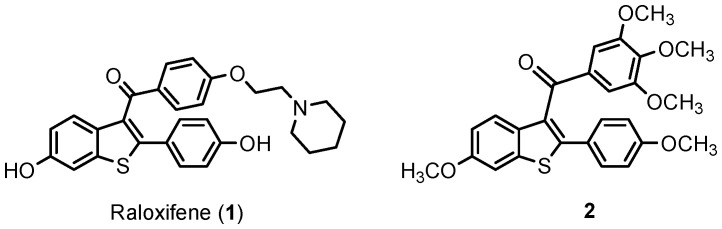
Representative examples of biologically active 3-acylbenzothiophene derivatives.

Heterocycles containing sulfur as a heteroatom are an important class of compounds with high utility in both organic and medicinal chemistry [6]. Benzothiophene is a heterocyclic scaffold among sulfur heterocycles and a common moiety found in many biologically active natural and synthetic therapeutic products, representing a very important pharmacophore [7]. Compounds containing the benzothiophene moiety have been present at various stages of drug development as anti-inflammatory agents [8], urokinase inhibitors [9], endothelial cell activation inhibitors [10] and cognition-enhancing agents with potential application in Alzheimer’s disease (AD) [11], amongst others. Also, 3-acylbenzo[*b*]thiophenes have received considerable attention due to their presence in different bioactive compounds, such as raloxifene (**1**) and 6-methoxy-3-(3,4,5-trimethoxybenzoyl)-2-(4-methoxyphenyl)benzothiophene (**2**). Both molecules have been reported as exclusive selective estrogen receptor modulators (SERMs) and anti-tubulin agents that may prevent osteoporosis in postmenopausal women (Figure 1) [12,13].

**Figure 2:**
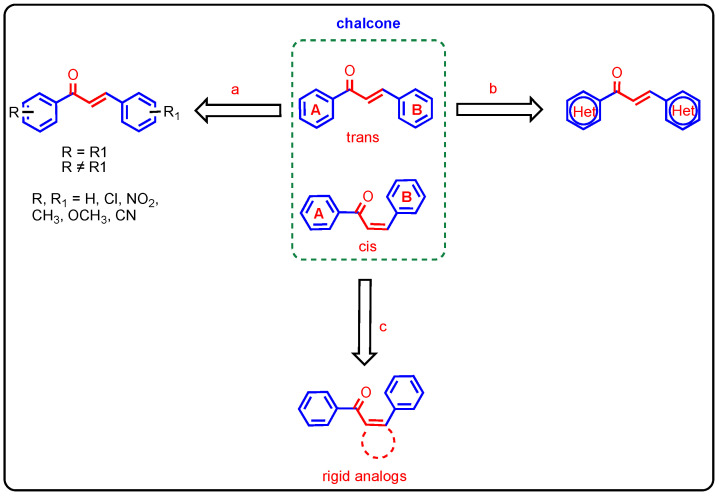
General structure of the chalcone scaffold and some of the most common structural modifications.

Chalcones are small molecules with two aryl rings in their structure, A and B, joined together by an α,β-unsaturated carbonyl group (Figure 2). In nature, they are present in vegetables, spices and fruits and can exist as both *cis* and *trans* isomers, although the *trans* is thermodynamically more stable. Chalcones and their synthetic derivatives have shown potential activities against different neurological diseases, particularly AD [14]. This simple scaffold can be widely modified, e.g., (a) substitution on the phenyl ring with different functional groups; (b) substitution of rings A and/or B with different heteroaryl rings; (c) fusion of the α,β-unsaturated carbonyl group in a rigid structure as benzofuran or benzothiophene (Figure 2).

Chalcones are highly versatile compounds, possessing a wide range of pharmacological activities, making them a valuable framework in drug discovery. Chalcones are important building blocks of various bioactive chemicals, exhibiting anti-inflammatory, antimicrobial, antituberculotic, antimalarial, anticancer, antidiabetic and antiviral properties [15]. Furthermore, different classes of chalcone derivatives exhibit cholinesterase (ChE) inhibition, which also depends on the substitution patterns of the two aromatic rings [16].

**Figure 3:**
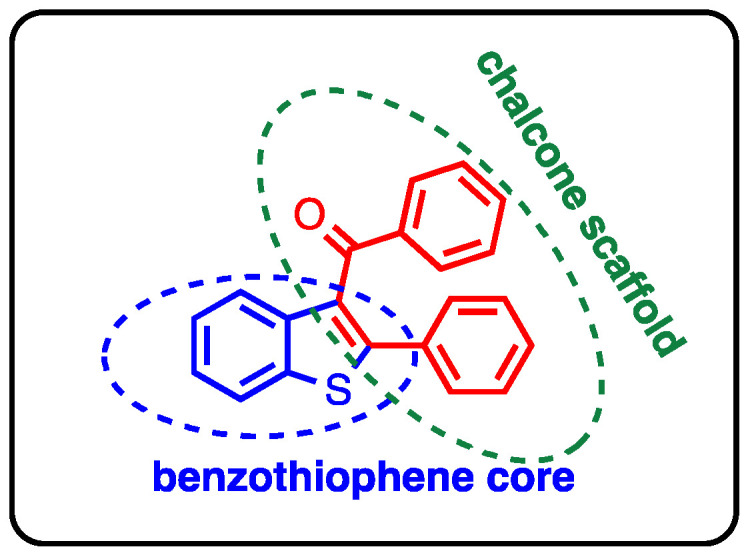
Structure of benzothiophene–chalcone hybrids.

The fusion of two or more pharmacophores in a single scaffold is a strategy for increasing biological activities and improving pharmacokinetic properties such as absorption and distribution. This approach can increase the effectiveness and allow for oral administration. For a promising drug candidate for AD, penetration ability through the blood–brain barrier (BBB) is essential. The design of hybrid compounds may increase the BBB crossing efficiency. 3-Benzoylbenzothiophenes are structurally benzothiophene–chalcones hybrids (Figure 3). In this new scaffold, the chalcone moiety is blocked into a more rigid conformation of the benzothiophene ring.

AD is a neurodegenerative disease that causes cognitive decline and memory loss. Although the exact cause of this disorder is not yet fully understood, several risk factors, including oxidative stress, τ protein aggregation, inflammation, amyloid-β deposits and low levels of acetylcholine (ACh), seem to play a crucial role in developing AD [17,18]. Some U.S. Food and Drug Administration (FDA)-approved drugs, such as donepezil and galantamine, inhibit ChE. However, their short half-lives, low bioavailability and toxicity limit their efficacy [19].

Ismail and co-workers synthesized thiophene derivatives and screened the new molecules for their acetylcholinesterase (AChE) inhibitory activity. Some of the tested compounds showed higher or similar inhibitory activity in relation to donepezil, a drug approved by the FDA for the treatment of dementia in mild, moderate and severe AD [20]. Several new heterocycles that include the benzo[*b*]thiophene motif were synthesized and tested for their ability to inhibit AChE. The results indicated that all analogues exhibited AChE activity, with IC_50_ values ranging between 20.8 and 121.7 µM [21].

**Figure 4:**
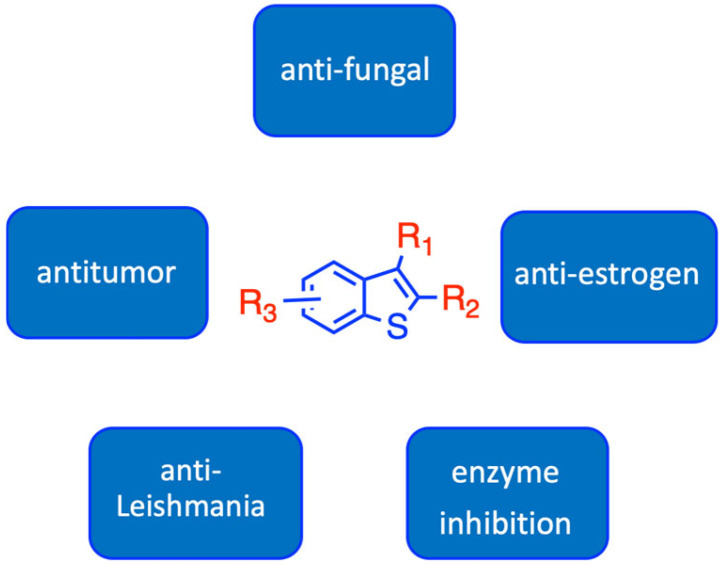
Most relevant biological activities of 2-phenylbenzothiophene derivatives.

Several studies have described different biological activities for the 2-phenylbenzothiopene scaffold. The most relevant ones are antitumor [22], anti-fungal [23], anti-estrogen [24], enzyme inhibition [25] and anti-Leishmania [26] (Figure 4).

In our efforts to develop novel compounds for treating AD, we evaluated the correlation of the structural features and AChE/BChE inhibitory activities of the synthetized phenylbenzothiophene derivatives.

All of the above considerations, and previous reports regarding the inhibition of AChE and BChE exhibited by benzothiophenes and chalcones, inspired us to synthesize and characterize 3-acylbenzothiophenes. In this work, we describe the synthesis, biological evaluation, ADME properties calculation and molecular docking of a series of 2-phenylbenzothiophene and 2-benzoyl-3-phenylbenzothiphene derivatives.

## 2. Results and Discussion

### 2.1. Chemistry

Considerable efforts have been made to develop efficient methods for the synthesis of 2-substituted benzo[*b*]thiophenes and 3-acylbenzothiophene derivatives. The classical approaches to synthesizing 2-substituted benzo[*b*]thiophenes are normally focused on (a) a radical cascade cyclization of 2-alkynylthioanisoles with α-oxocarboxylic acids with AgNO_3_ [27], (b) a coupling cyclization reaction of *o*-bromoalkynylbenzenes with different thiols upon lithium halogen exchange at 78 °C [28], (c) an annulation of alkynylbenzenes using sodium sulfide (Na_2_S) in *N*-methyl-2-pyrrolidine (NMP) at 180 °C [29] and (d) a cyclization of 2-alkylphenyl alkyl sulfoxides catalyzed by mercury chloride [30]. A typical method includes the Friedel–Crafts acylation on preformed benzothiophenes using acyl chlorides. However, this strategy presents several limitations, such as poor regioselectivity, the formation of environmentally harmful HCl, the use of excess Lewis acid, limited functional groups tolerability and poor yields when deactivated acyl chlorides are used [31].

**Scheme 1:**
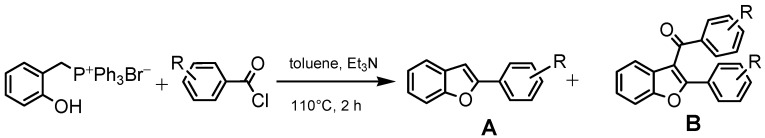
Previously synthesized 2-phenylbenzofurans (**A**) and 3-benzoyl-2-phenylbenzo[*b*]furans (**B**).

In our previous works, we have synthesized a series of 3-benzoyl-2-phenylbenzofurans under Wittig conditions. The reaction of 2-hydroxybenzyltriphenylphosphonium bromide with benzoyl chlorides and triethylamine in toluene leads to the expected 2-phenylbenzofurans **A** as well as to the 3-benzoyl-2-phenylbenzo[b]furans **B**. The simple procedure requires short reaction times and does not require the use of special equipment or reagents (Scheme 1) [32,33].

Although the Wittig reaction has been described in several articles for the preparation of 2-arylbenzofuran derivatives, the formation of secondary products was not mentioned. These findings encouraged us to investigate the formation of 3-acyl-2-phenylbenzothiophenes under these same conditions [34,35]. Also, in drug discovery, bioisosterism is used to reduce toxicity, change bioavailability or modify the activity of the lead compound and may change the metabolism of the lead.

**Scheme 2:**
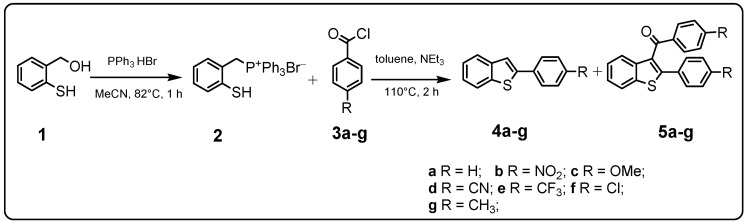
Synthetic route for obtaining 2-phenylbenzothiopenes **4a–4g** and 3-benzoyl-2-phenylbenzothiopenes **5a–5g**.

2-Phenylbenzotiophenes **4a–4g** and 3-acyl-2-phenylbenzothiophenes **5a–5g** were efficiently prepared by an intramolecular Wittig reaction (Scheme 2 and Table 1) [34,35,36,37]. 2-Mercaptobenzenemethanol **1** was taken as the starting material. The first step involved the formation of Wittig reagent 2-(sulphanylphenyl)methyltriphenylphosphonine bromide (**2**) via the coupling reaction of **1** with triphenylphosphine hydrobromide in acetonitrile at 82 °C. The formation of the benzothiophene ring was achieved by a reaction between phosphonium salts **2**, convenient acyl chlorides **3a–3g** and triethylamine in dry toluene for 2 h at 110 °C. The obtained products were easily purified by silica gel chromatography, using a mixture of petroleum ether/ethyl acetate as eluents.

2-(4-Aminophenyl)benzothiophene **4h** and 2-(4-aminophenyl)-3-(4-aminobenzoyl)benzothiophene **5h** were prepared from nitro-compounds **4b** and **5b**, respectively, by reduction with stannous chloride (SnCl_2_) in refluxing ethanol for 12 h (Table 2) [34].

The hydroxy derivatives 2-(4-hydroxyphenyl)benzothiophene **4i** and 3-(4-hydroxybenzoyl)-2-(4-hydroxyphenyl)benzothiophene **5i** were obtained from the corresponding methoxy-compounds **4c** and **5c** by heating a mixture of the methyl aryl ether and pyridine hydrochloride in a stopped round bottom flask under microwave irradiation (300 W) for 10–15 min (Table 3) [38].

The chemical structures of all the synthesized compounds were confirmed by different spectroscopic techniques such as ^1^H NMR, ^13^C NMR and mass spectrometry. Generally, this reaction afforded the 2-phenylbenzothiophenes **4a–4g** in good yields when we used acyl chlorides substituted with electron-donating groups such as methyl and methoxy. On the other hand, the best yields for the 3-benzoyl-2-phenylbenzothiophenes **5a–5g** were obtained using acyl chlorides with para-substituted electron-withdrawing groups or atoms such as nitro, cyan, trifluoromethyl and chlorine.

### 2.2. Biological Activity

The potential effects of the synthesized compounds **4a–4i** and **5a–5i** on AChE and BChE were performed using electrophorus electricus AChE and equine serum BChE due to their low cost and high degree of similarity with their respective human enzymes. For the first screenings, they are excellent and widely used in vitro models. The IC_50_ values of the most active compounds on both ChE were calculated. Table 4 shows the inhibitory effect of the studied compounds.

Eighteen benzothiophene derivatives (nine from series **4** and nine from series **5**) have been synthesized and studied for their ChE inhibition. As reported in Table 4, five 2-phenylbenzotiophenes (**4a**, **4d**, **4e**, **4f** and **4g**) are insoluble in dimethyl sulfoxide (DMSO), and the others show the low inhibition of both AChE and BChE. As a main conclusion, compounds mono-substituted at position 2 of the scaffold (series **4**) proved to display low inhibition profiles against both enzymes and poor solubility in organic solvents.

On the other hand, all 3-benzoyl-2-phenylbenzothiophenes (**5a–5i**) are soluble in DMSO, probably due to the presence of the carbonyl group in their structures. In addition, all the compounds **5a–5i** displayed inhibition against BChE, with percentages of inhibition generally higher than those of series **4** compounds. Based on the inhibition percentages observed at 50 μM, IC_50_ values for the BChE inhibition of three compounds (**5a**, **5h** and **5i**) were calculated. Even if the three of them present IC_50_ in the low micromolar range, two compounds are slightly less active than the standard inhibitor, galantamine (IC_50_ = 28.1 μM). Nevertheless, compound **5h** showed an IC_50_ value similar to that of galantamine [39].

Amino and hydroxyl substituents in the 3-benzoylbenzothiophenes **5h** and **5i**, respectively, determine a good inhibition toward BChE, showing IC_50_ values of 24.3 and 59.6, respectively. Except for the compound **5h**, all the other compounds in series **5** showed inhibition against AChE. In particular, compound **5f** has the best AChE inhibitory activity. Therefore, its IC_50_ has been calculated (IC_50_ = 62.1 μM).

Comparing both series, especially the most interesting and selective compound against BChE, compound **5h** [2-(4-aminophenyl)-3-(4-aminobenzoyl)benzothiophene], with the similarly substituted **4h** [2-(4-aminophenyl)benzothiophene], the introduction of the substitution at position 3 has significantly improved the inhibition towards BChE, maintaining the same pattern against AChE. This profile is maintained for all eight substitution patterns, from **a** to **i**.

From this preliminary screening, it seems that naked aryl rings or the presence of electron-donating groups at position 4 of both aryl rings attached to positions 2 and 3 of the benzothiophene scaffold are interesting substitution patterns for the desired activities. Chemical routes for synthesizing other substituted benzothiophenes based on these first observations must be developed in order to increase the chemical space around the scaffold.

### 2.3. Cell Viability in SH-SY5Y Cells

**Figure 5:**
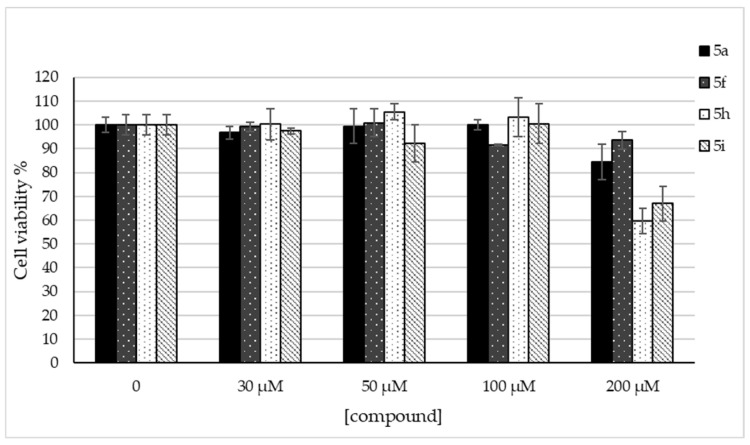
Cell viability of SH-SY5Y cells in the presence of the most active compounds (**5a**, **5f**, **5h** and **5i**).

Compounds **5a**, **5h** and **5i** have exhibited the most potent activity against BChE, and compound **5f** has exhibited the most potent activity toward AChE. Therefore, their impact on the viability of SH-SY5Y cells (neuroblastoma cell line) has been studied. For cell viability experiments, the compounds were tested at different concentrations (0, 30, 50, 100 and 200 μM) for 24 h and analyzed using the MTT test. The results of the cell viability tests are presented in Figure 5.

The results indicate that these inhibitors exhibited no cytotoxic effects on cell viability at the concentration at which they inhibited their inhibitory activity against ChE.

### 2.4. Absorption, Distribution, Metabolism and Excretion (ADME) Studies

The prediction of ADME and pharmacokinetic properties, as well as the drug-likenesses of the studied compounds, has been investigated using the SwissADME webserver [40]. Properties such as the molecular weight (MW), volume, number of rotatable bonds (RB), number of hydrogen bond acceptors (HBA) and donors (HBD), and topological polar surface area (TPSA), lipophilicity and water solubility of the compounds are essential parameters to be considered in a drug development program, even at earlier stages.

Table A1 (Appendix A) shows the physicochemical properties, solubility descriptors, ADME parameters and violations of drug-likeness rules for all the synthesized compounds and for galantamine, the reference compound. Several filters have been used to calculate the physicochemical parameters and evaluate the drug-likeness of the synthesized compounds [41,42,43,44,45]. Some of these filters generally assume that an orally active drug should not violate the above criteria more than once. For the compounds studied, MW varies between 210 and 450 g/mol, which falls within the optimum range for drug-likeness (150 ≤ MW g/mol ≤ 500).

The number of RB indicates a structure’s flexibility, which is important as a very flexible molecule that may not be selective towards the target. The number of RB in our compounds is in the range of 1–5. The numbers of HBA and HBD were 0–5 and 0–2, respectively (HBA ≤ 10; HBD ≤ 5).

TPSA is a way to estimate the overall polarity of a compound. The polarity of a compound affects the solubility and the availability of the drug. However, a high polarity may also lead to high efflux rates. TPSA values were highest for compound **5b** = 136.95 and lowest for compounds **4a**, **4f** and **4g** = 28.24 (20 < TPSA < 130 Å^2^).

The partition coefficient (logP) is an experimental measure used to evaluate the lipophilicity of a compound. It is generally computed as the logarithm of the ratio of the concentrations measured in a system comprising a water phase and an n-octanol phase. There are different methods for the prediction of logP. Table 4 displays the ilogP, XlogP3, WlogP, MlogP, SILICOS-IT and consensus logPo/w of our virtual screening’s compounds [46]. The consensus logPo/w is the arithmetic mean of the values predicted by the five proposed methods. According to Lipinski’s Rule of 5, an oral drug should have a MlogP value < 4.15 [41]. Eleven compounds in our list conform to the requirements for lipid solubility.

Table A1 also shows the ESOL (Estimated SOLubility in water) values and solubility categories for the synthesized compounds. The minimum and maximum ESOL values were −7.66 and −4.57 for compounds **5e** and **4h**, respectively. ESOL estimates the aqueous solubility of lead directly from the chemical structure [47]. Thus, compound **4h** is the most water-soluble (moderately soluble) molecule among the hits from our virtual screening (ESOL = 4.57). Six compounds (**5a**, **5b**, **5c**, **5e**, **5f** and **5g**) have values greater than −6; thus, they are in the poorly soluble class (−6 < ESOL < 0).

**Figure 6:**
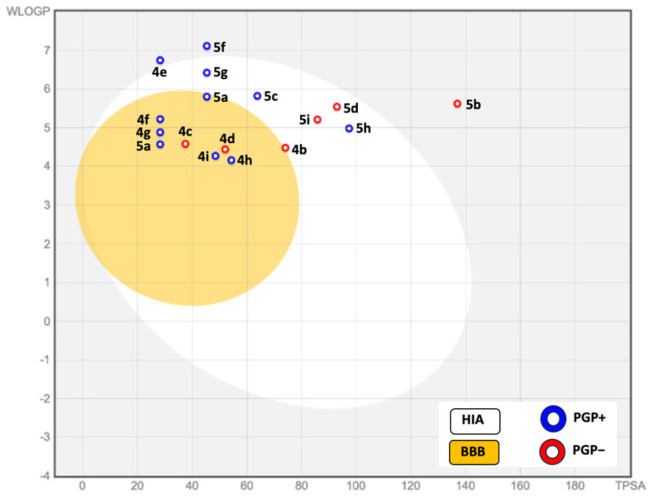
BOILED-Egg construction using two physicochemical descriptors, lipophilicity (WlogP) and TPSA, for the compounds investigated here. The white region represents the physicochemical space of gastrointestinal absorption and the yellow region represents brain penetration.

The BOILED-Egg (Brain Or IntestinaL EstimateD) is a simple model based on logP and TPSA for predicting small molecule human gastrointestinal absorption (HIA) and BBB penetration [48]. The yolk (yellow region) represents a high likelihood of brain penetration, while the white area (intestinal tract) represents a high likelihood of passive absorption by the GI tract. When we plotted WLOGP and TPSA of the virtual screening hits on the BOILED-Egg (Figure 6), fourteen ligands were inside the egg, the area representing suitable physicochemical space for oral bioavailability [48]. Eight molecules inside the yellow are predicted to be distributed in the brain tissue (**4a**, **4c**, **4d**, **4f**, **4g**, **4h**, **4i** and **5a**). Nonetheless, six of these compounds (**4a**, **4f**, **4g**, **4h**, **4i** and **5a**) seem to be P-glycoprotein (PGP+) substrates and, thus, are likely to be expelled from the central nervous system. Six molecules are distributed in the white zone and therefore absorbed in the gastrointestinal tract (**4b**, **5c**, **5d**, **5g**, **5h** and **5i**). Although three molecules are in the grey area (**4e**, **5b** and **5f**), they are still close to the egg’s white and would gain better bioavailability profiles during a drug development phase. Only benzothiophene **5e** resulted out of range.

Lipinski’s rule of five is widely used in predicting the oral bioavailability of small molecules. Eleven benzothiophenes (**4a**, **4b**, **5b**, **4c**, **5c**, **4d**, **5d**, **4h**, **5h**, **4i** and **5i**) followed Lipinski’s rules (Table 4), while seven benzothiophenes (**5a**, **4e**, **5e**, **4f**, **5f**, **4g** and **5g**) violated the criteria only one time. Most compounds also agreed with other models of drug-likeness such as Ghose, Veber and Egan [42,43,44]. Based on the results of the ADME calculations, all the investigated compounds present a bioavailability score of 55%. The bioavailability score (ABS) depends on passing or violating Lipinski’s rule of five. At biological pH, a compound is expected to have >10% bioavailability (F) in rats only if it passes Lipinski’s rule of five with ABS 0.55 (i.e., 55% chances of F > 10% in rats) [49].

### 2.5. Molecular Docking Study

**Figure 7:**
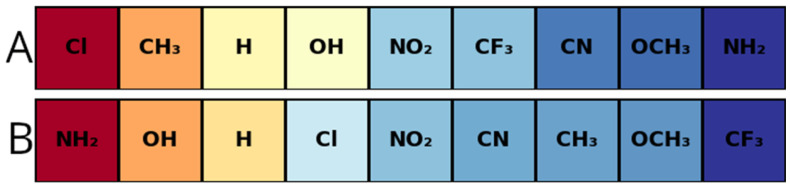
AChE (**A**) and BChE (**B**) substituents’ inhibitory trends obtained by the docking calculations.

To investigate the structure–activity relationships (SAR) and predict binding affinities, we focused this study on scaffold **5**, after unpromising experimental results from compounds in series **4**. SAR analysis has been performed, and the ligand activity trends based on substituents for both AChE and BChE are depicted in Figure 7.

For AChE, chlorine and methyl substituents exhibited the highest inhibitory potencies, suggesting the presence of a small pocket that can accommodate these groups. The hydroxyl substituent showed only modest activity, while the amino group was inactive. This trend suggests that there are no hydrogen bond interactions involving the ligand as the donor in this position. These theoretical predictions are aligned with the experimental results, being a good tool for the future design of new ligands.

In contrast, for BChE, the hydroxyl and amino substituents displayed the highest inhibitory activities, indicating the formation of hydrogen bonds at this position. Furthermore, the reasonably potent inhibition observed for the unsubstituted scaffold suggests that smaller substituents are favored, potentially due to a small available volume in the binding pocket. These theoretical predictions also corroborate the experimental trends observed.

From the docking analysis, the binding poses of all molecules assume an extremely conserved binding pose both in both AChE and BChE. As such, the different substituents do not appear to significantly influence the binding mode. Studying protein interactions could help understanding the binding mode of the most promising candidates within the study, as well as guide us in the design of more potent and selective inhibitors. Therefore, compounds **5f** and **5h** have been selected for the theoretical study to better understand their binding profiles against AChE and BChE, respectively.

**Figure 8:**
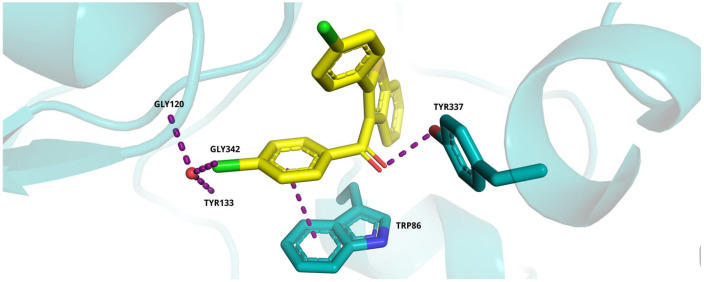
Best pose found for compound **5f** (in yellow) in the binding pocket of AChE. Interactions with different residues are highlighted with purple discontinue lines.

For the AChE, intermolecular interactions analysis shows a hydrogen bond between the carbonyl oxygen and TYR337 and π-stacking between the aromatic ring of benzoyl and TRP86 (Figure 8). The benzoyl substituent is obstructed by a structural water molecule stabilized by the protein side chains, explaining the preference for small groups. The phenyl substituent has no significant interactions.

We compared the pose we obtained for compound **5f** with the crystallographic pose of galantamine binding AChE. The crystallographic structure (PDB ID 4EY6) was selected for analysis among other available crystallographic structures, as all displayed the same binding mode for this reference compound.

**Figure 9:**
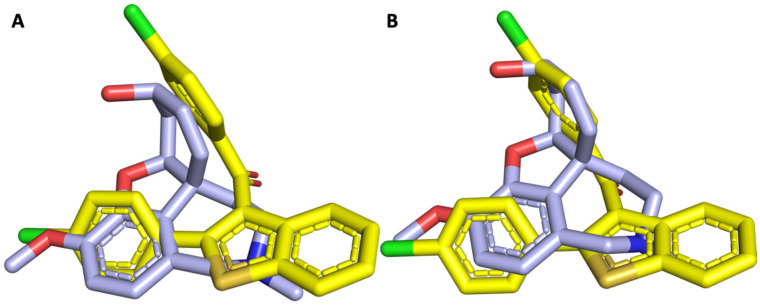
Comparison of the conformation of galantamine (blue) and ligand **5f** (yellow). (**A**) Relative position of the binding poses in the pocket. (**B**) Superimposition obtained when aligning the ShaEP field graph.

To compare the structural and conformational similarity of galantamine and the ligand **5f** in their binding modes with AChE, we utilized the SHaEP algorithm [50]. This algorithm compares the electrostatic potential at selected points around the molecules and aligns the resulting field graphs. By aligning the ligand **5f** with galantamine, we obtained a position highly similar to the computed binding pose, with a heavy atom RMSD of 1.1 Å. The superimposed binding poses and the field graph-aligned conformations are shown in Figure 9. The structural shape similarity was 76%, while the electrostatic potential (ESP) similarity of the field graph was 68%. These values indicate that the two molecules have very similar three-dimensional conformations and spatial occupancy, as well as similar electrostatic potentials. Their binding modes to the AChE protein occur in the same pocket with very similar configurations.

**Figure 10:**
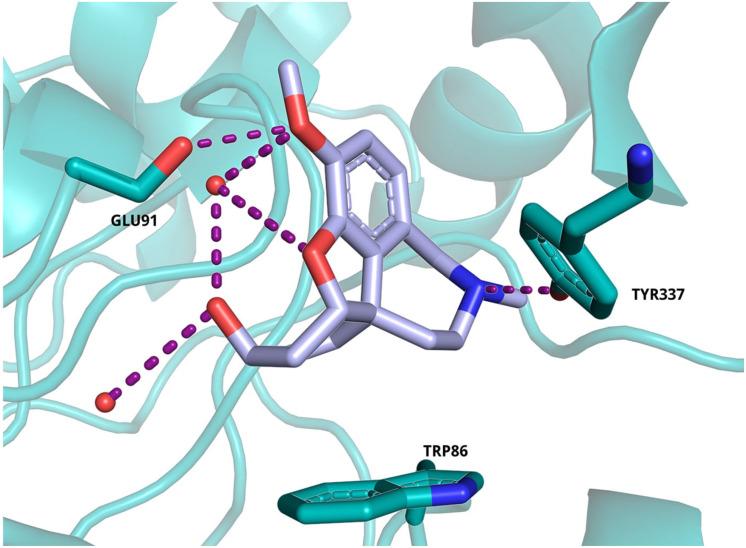
Crystallographic binding pose of galantamine (light purple) from PDB 4EY6. Interactions with different residues are highlighted with purple discontinue lines.

The binding pose of galantamine, shown in Figure 10, revealed intermolecular interactions comparable to those predicted for ligand **5f**. Galantamine forms a hydrogen bond with TYR337, and its position in the pocket is constrained by TRP86. Additionally, galantamine forms a hydrogen bond interaction with GLU91. However, its interaction with the solvent network differs: galantamine does not interact with the water molecule that constrains ligand **5f** but instead forms strong hydrogen bonds with two water molecules, which are also stabilized by interactions with protein side chains. This results in a very stable and rigid conformation of galantamine. Given its inherent rigidity, the entropic contributions of this stabilization may be minimal compared to the formation of strong hydrogen bonds.

**Figure 11:**
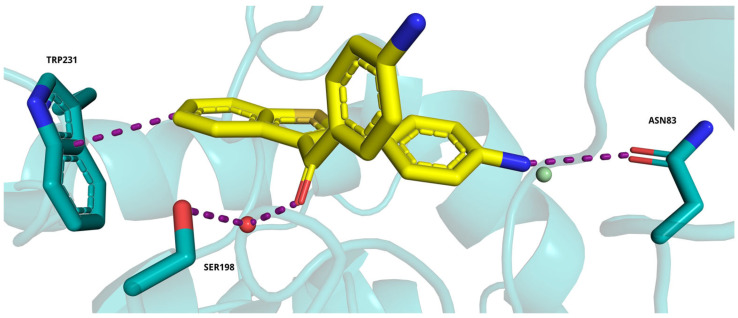
Best pose found for compound **5h** (in yellow) in the binding pocket of BChE. The position of the displaced crystal water molecule is highlighted in green. Interactions with different residues are highlighted with purple discontinue lines.

For BChE, the binding pose is stabilized by a T-shaped π-stacking interaction with TRP231 and a hydrogen bond between the carbonyl oxygen and a structural water molecule, which is further stabilized by interactions with SER198, GLY116 and GLY117 (Figure 11). The benzoyl substituent occupies an unobstructed region of the binding pocket, while the other substituent is constrained by a less tightly bound structural water molecule.

The analysis of the temperature factors across multiple BChE crystal structures (average of 0.088 when normalized with the Wang method [51], compared to 0.063 for this water in 7QHD) suggests this particular water molecule exhibits higher mobility and is not as firmly anchored as other structural waters. Ligands bearing a primary amine or hydroxyl substituents are likely able to displace this water molecule to form a more favorable hydrogen bonding interaction with ASN83, explaining their enhanced potency.

**Figure 12:**
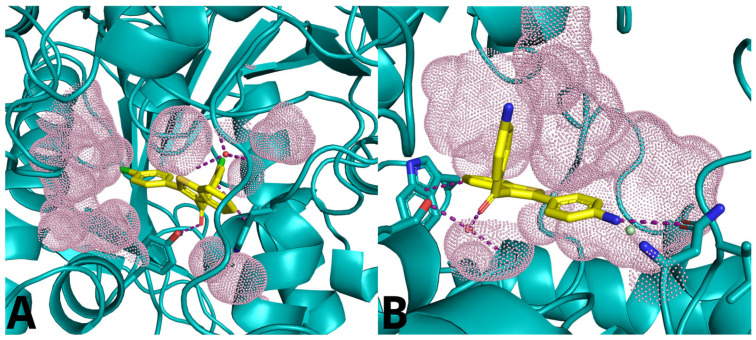
Analysis of solvent accessible surface areas for AChE (**A**) and BChE (**B**) in the presence of compounds **5f** and **5h**, respectively. Interactions with different residues are highlighted with purple discontinue lines.

The analysis of solvent accessible surface areas revealed that a large empty sub-pocket is available on the side of the phenyl substituent in the binding mode with AChE, while in BChE, a wide amount of available space is available on the side of the ligand but not extending the substituents. These concepts are depicted in Figure 12.

While the inhibitors obtained lack potency and AChE selectivity, the SAR analysis and computational insights may guide in the design of more active and selective candidates. In AChE, ligand expansion is feasible on the phenyl substituent, whereas in BChE, space allows growth on the side.

To enhance AChE selectivity and activity, extending the scaffold asymmetrically could improve AChE binding while sterically impeding BChE. Retaining the chlorine on the benzoyl side and replacing the substituent on the phenyl ring with a bulky, inflexible moiety such as a cyclohexane or an aromatic ring may achieve this. Moreover, given the presence of polar amino acids in the available AChE sub-pocket, it may be advantageous to provide some hydrogen bond acceptors and donors to explore interactions. Moreover, substituents on the aromatic ring of the scaffold could disrupt the T-shaped π-stacking interaction with BChE without hindering AChE binding. Combined with growth towards the sub-pocket, this could yield potent, selective AChE inhibitors. Overall, the different sub-pocket topologies in AChE and BChE provide opportunities for rationally designing inhibitors optimized for selectivity and potency towards AChE.

A major difference between galantamine and our ligands is the rigidity of the molecules. The number of rotatable bonds (NRot) in galantamine is only 1, while **5f** has a value of 3. However, NRot does not account for the fact that in galantamine, the only freely rotatable moiety is a small terminal ether, whereas in **5f**, the rotatable bonds cause the aromatic rings to rotate, leading to greater conformational differences. Another significant difference is the presence of several oxygen atoms in the structure of galantamine, which allows for the formation of a strong network of hydrogen bonds; as such, it may be beneficial to explore groups that reproduce the same hydrogen bonds as galantamine to increase the binding interaction with AChE.

## 3. Materials and Methods

### 3.1. Chemistry

The starting materials, solvent and reagents were obtained from commercial suppliers (Sigma–Aldrich, Darmstadt, Germany) and were used without further purification. All reactions were performed under an N_2_ atmosphere. Analytical thin layer chromatography (TLC) was carried out on silica gel 60 F254 plates (0.25 mm), visualized by exposure to UV light (254 nm). Column chromatography purifications were performed using an Aldrich silica gel (60–120) mesh size. Melting points were determined on a Stuart Scientific SMP 11 melting point apparatus and are uncorrected. The concentration and evaporation of the solvent after the reaction or extraction were carried out on a rotary evaporator (Büchi Rotavapor, Cornaredo (MI), Italy) operating at reduced pressure. ^1^H NMR and ^13^C NMR spectra were recorded with a spectrometer (Varian INOVA, Milano, Italy) operating at a field of 14.4 T (600 MHz for ^1^H, 150.8 MHz for ^13^C) and using CDCl_3_ as a solvent. Chemical shifts are reported in ppm (δ) relative to TMS (tetramethylsilane) as an internal standard. The 150.8 MHz ^13^C spectra were acquired under proton decoupling conditions with a 36,000 Hz spectral width, 5.5 µs (60° tip angle) pulse width, 1 s acquisition time and 4 s delay time. The long relaxation time was needed to observe some quaternary carbons. Coupling constants J are expressed in hertz (Hz). Spin multiplicities are given as s (singlet), d (doublet), dd (doublet of doublets), m (multiplet) and apparent triplet (app t). GC-MS low-resolution mass spectrometric experiments were carried out on a Saturn 2000 ion-trap coupled with a Varian 3800 gas chromatograph (Varian, Walnut Creek, CA, USA) operating under EI conditions (electron energy 70 eV, emission current 20 mA, ion-trap temperature 200 °C, manifold temperature 80 °C, automatic gain control (AGC) target 21.000), with the ion trap operating in scan mode (scan range from *m*/*z* 40 to 600 at a scan rate of 1 scan/s). Aliquots of 1 µL of solutions 1.0 × 10–5 M in dichloromethane (DCM) have been introduced into the gas chromatographer inlet. An Agilent J&W VF-5ms Low-bleed/MS GC capillary column (30 m, 0.25 mm i.d., 0.25 mm film thickness) (Agilent Technologies Inc., Wilmington, DE, USA) was used. The oven temperature was programmed from 100 °C (held for 2 min) to 325 °C at 30 °C/min (held for 10 min). The temperature was then ramped to 350 at 20 °C/min. The transfer line was maintained at 250 °C and the injector port (30:1 split) was maintained at at 290 °C. HRMS-positive ESI-MS spectra were recorded with a high-resolution LTQ Orbitrap Elite™ mass spectrometer (Thermo Fisher Scientific, Bremen, Germany). The solutions were infused at a flow rate of 5.00 μL/min into the ESI source. Spectra were recorded in the range of *m*/*z* 100–1500 with a resolution of 240,000. The instrumental conditions were as follows. Spray voltage 3500 V, capillary temperature 275 °C, sheath gas 5–10 (arbitrary units), auxiliary gas 3 (arbitrary units), sweep gas 0 (arbitrary units) and probe heater temperature 50 °C.

### 3.2. Biological Activity

#### 3.2.1. Determination of Cholinesterases Inhibition

AChE (EC EC 3.1.1.7) from electrophorus electricus and equine serum BChE (EC 3.1.1.8) were used for the inhibitory assays. ChE inhibitory activity was evaluated using Ellman’s method [52], as previously described, with slight modifications [53]. The reaction mixture was prepared by dissolving acetylthiocholine iodide (1.5 mM), 5,5’-dithiobis-2-nitrobenzoate (DTNB) (1.5 mM) and the inhibitor (at the desired concentrations) or DMSO alone (control) in 0.1 M phosphate buffer (pH 8.0) to a final volume of 200 µL. The enzyme was added to the reaction mixture and the absorbance was monitored at 405 nm as soon as it was added. This test is based on the AChE enzyme hydrolyzing acetylthiocholine to produce acetate and thiocholine. Thiocholine reacts with DTNB to produce a yellow-colored anion. For the BChE assay, we followed the same procedures as above but used BChE as the enzyme and S-butyrylthiocholine chloride (BTCI) as the substrate. To determine the IC_50_ values, which are the concentration that results in 50% inhibition of the ChE activity, we analyzed and fitted the dose–response curves (see Supplementary Materials). The IC_50_ values displayed represent the mean ± standard deviation for two/three independent assays. Galantamine is considered as a positive control under the same experimental conditions.

#### 3.2.2. Cell Viability

Human neuroblastoma cell line SH-SY5Y was obtained from ICLC Cell bank (cat. # HTL95013). The cells were cultured in high-glucose DMEM supplemented with 10% fetal bovine serum, 100 units/mL of penicillin and 100 µg/mL of streptomycin (all from Gibco, Life sciences; Thermo Fisher Scientific, Waltham, MA, USA) and maintained at 37 °C in a humidified atmosphere of 5% CO_2_ and 95% air. Cell viability was detected by the colorimetric 3-(4,5 dimethylthiazol-2-yl)-2,5-diphenyltetrazolium bromide (MTT) assay, as previously described, with minor modifications [39]. Briefly, 1.5 × 10^4^ cells/well were seeded in 96-well plates and incubated for 24 h with different concentrations (30–200 μM) of compounds. After incubation, 100 μL of the MTT reagent (0.5 mg/mL in DMEM) was added, and the cells were incubated for 3 h at 37 °C. The resulting violet formazan precipitate was dissolved in DMSO and quantified by spectrophotometry at 570 nm using a microplate reader (VANTAstar_BMG LABTECH GmbH, Offenburg, Germany). Viability data were reported in the percentage of control for each compound.

### 3.3. Computational Methods

#### 3.3.1. Calculation of ADME Properties

The SwissADME web tool (http://www.swissadme.ch, accessed on 25 August 2023) gives free access to a pool of fast yet robust predictive models for physicochemical properties, pharmacokinetics, drug-likeness and medicinal chemistry friendliness parameters through the input of the molecular structure [40].

#### 3.3.2. Molecular Docking

Molecular docking with AutoDock Vina [54] and molecular dynamics (MD) simulations with openMM [55] were employed to analyze ligand interactions with AChE (PDB ID: 8AEN) and BChE (PDB ID: 7QHD). Docking predicted the binding poses of ligands within the active sites. MD simulations using the AMBER14 [56] force field refined these poses and evaluated complex stability. The complexes were solvated in TIP3P water [57], neutralized with appropriate counterions, energy-minimized and equilibrated in NVT and NPT ensembles before production runs of 50 ns at 295K. MM/PBSA binding energy calculations were used as a scoring function to select the ligand poses [58].

## 4. Conclusions

Benzo[*b*]thiophene **4a–i** and **5a–i** were synthesized through an intramolecular Wittig reaction involving the coupling of triphenylphosphine hydrobromide **2** with acyl chloride **3a–g**. The effects of the synthesized compounds **4a–4i** and **5a–5i** on AChE and BChE were performed on electrophorus electricus AChE and equine serum BChE. Benzothiophene-chalcone hybrids from series **5** proved to be better inhibitors of both enzymes, compound **5f** being the best AChE inhibitor and compound **5h** being the best BChE inhibitor. Compound **5h**’s IC_50_ value against BChE proved comparable to that of the standard inhibitor galantamine. Compounds **5a**, **5h** and **5i**, with the most potent activity against BChE, and compound **5f**, with the most potent activity against AChE, were studied for their impact on the viability of SH-SY5Y cells. At their IC_50_ concentrations, all these benzothiophenes exhibited no cytotoxic effects. Comparative physicochemical and pharmacokinetic properties of the compounds with the commonly prescribed drugs revealed a high probability of all the compounds reaching the brain. Finally, molecular docking simulations assisted in explaining the structure–activity relationships of the families of compounds. The different sub-pocket topologies in AChE and BChE and the interaction with the synthetized molecules suggest the rational design of optimized inhibitors to achieve more selectivity and potency towards ChE. In conclusion, these results may inspire the design of a new series of benzothiophene-containing derivatives for Alzheimer’s disease.

## Figures and Tables

**Table 1:** Structures of 2-phenylbenzothiophenes **4a–g** and 3-benzoyl-2-phenylbenzothiophens **5a–g**. Effect of different benzoylchlorides on the product ratio of **4** and **5**.

Product 4a–g		Product 5a–g	Yield 4/5 (%)
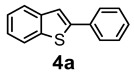	+	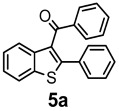	56/28
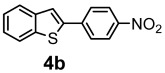	+	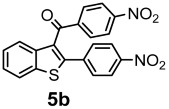	12/63
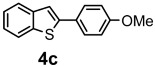	+	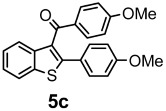	21/9
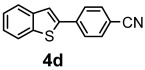	+	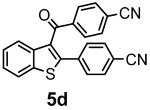	25/50
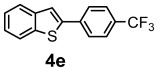	+	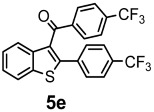	12/38
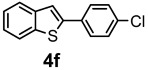	+	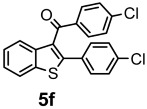	31/47
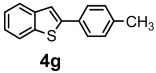	+	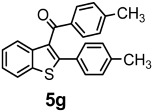	19/9

**Table 2:** Synthetic methodology and structures of 2-phenylbenzothiophene **4h** and 3-benzoyl-2-phenylbenzothiophene **5h** and related yields.

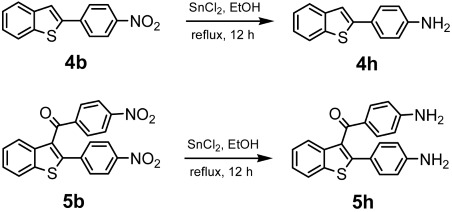			
Product	Yield	Product	Yield
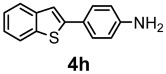	78%	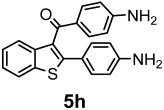	80%

**Table 3:** Synthetic methodology and structures of 2-phenylbenzothiophene **4i** and 3-benzoyl-2-phenylbenzothiophene **5i** and related yields.

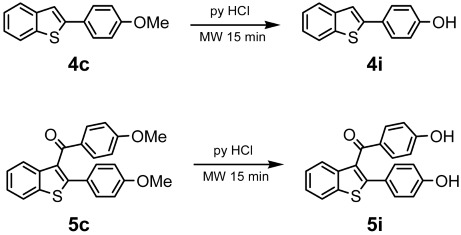			
Product	Yield	Product	Yield
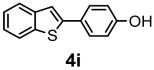	90%	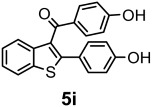	85%

**Table 4:** Percentage of inhibition (I %) at 50 µM and IC_50_ values of phenylbenzothiophene derivatives against electrophorus electricus AChE and equine serum BChE.

Compounds	AChE (I %)	BChE (I %)	Compounds	AChE IC_50_ (µM) (I %)	BChE IC_50_ (µM) (I %)
**4a**	Ins.	Ins.	**5a**	(20.80 ± 4.20)	83.40 ± 1.20 (35.70 ± 0.60)
**4b**	(8.20 ± 2.40)	(2.29 ± 3.23)	**5b**	(11.00 ± 0.04)	(16.80 ± 0.40)
**4c**	(9.60 ± 1.50)	(1.20 ± 0.01)	**5c**	(3.50 ± 0.10)	(11.90 ± 0.20)
**4d**	Ins.	Ins.	**5d**	(4.50 ± 3.90)	(14.00 ± 3.00)
**4e**	Ins.	Ins.	**5e**	(10.00 ± 1.10)	(3.10 ± 0.90)
**4f**	Ins.	Ins.	**5f**	62.10 ± 1.20 (39.90 ± 0.10)	(23.10 ± 0.70)
**4g**	Ins.	Ins.	**5g**	(28.30 ± 8.40)	(13.10 ± 1.04)
**4h**	NI	(10.20 ± 0.42)	**5h**	NI	24.30 ± 0.50 (58.30 ± 1.40)
**4i**	(8.19 ± 1.55)	(13.57 ± 1.81)	**5i**	(19.30 ± 0.90)	59.60 ± 11.60 (42.20 ± 4.70)

Ins. = insoluble; NI = none inhibition. I % and IC_50_ values represent the mean ± standard deviation for three independent assays.

## Data Availability

Data is available in the Supplementary Materials. Extra data information is available under request to the authors.

## Supplementary Materials

The following supporting information can be downloaded at: https://www.mdpi.com/article/10.3390/molecules29163748/s1. General Experimental Information, General procedure for the preparation of 2-mercaptobenzyltriphenylphosphonium bromide (2), General procedure for the preparation of 2-phenylbenzothiophenes 4a-g and 3-benzoyl-2-phenylbenzothiophenes 5a-g, 2-Phenylbenzothiophene (4a), 3-Benzoyl-2-phenylbenzothiophene (5a), 2-(4-Nitrophenyl)benzothiophene (4b), 3-(4-Nitrobenzoyl)-2-(4-nitrophenyl)benzothiophene (5b), 2-(4-Methoxyphenyl)benzothiophene (4c), 3-(4-Methoxybenzoyl)-2-(4-methoxyphenyl)benzothiophene (5c), 4-(Benzothiophene-2-yl)benzonitrile (4d), 4-Cianophenyl-[2-(4-cianophenyl)-benzothiophen-3-yl]-methanone (5d), 2-(4-Trifluoromethylphenyl)benzothiophene (4e), 3-(4-Trifluoromethylbenzoyl)-2-(4-trifluoromethylphenil)benzothiophene (5e), 2-(4-Chlorophenyl)benzothiophene (4f), 3-(4-Chlorobenzoyl)-2-(4-chlorophenyl)benzothiophene (5f), 2-(4-Methylphenyl)benzothiophene (4g), 3-(4-Methylbenzoyl)-2-(4-methylphenyl)benzothiophene (5g), Procedure for the preparation of 2-(4-aminophenyl)benzothiophene (4h), Procedure for the preparation of 3-(4-aminobenzoyl)-2-(4-aminophenyl)benzothiophene (5h), Procedure for the preparation of 2-(4-hydroxyphenyl)benzothiophene (4i), Procedure for the preparation of 3-(4-hydroxybenzoyl)-2-(4-hydroxyphenyl)benzothiophene (5i); Figures S1–S38: NMR spectra; Figure S39: The dose-response curves to determine the IC_50_ values of the compounds 5a, 5h, 5i against BChE (A, B and C) and 5f against AChE (D). References [26,34,35,36,37,38,59,60,61] are cited in the Supplementary Materials.

## Appendix A

**Table A1:** Physicochemical and pharmacokinetic properties of benzothiophenes 4a-i and 5a-i and galantamine, the reference compound.

Comp.	Physicochemical Properties							Lipophilicity						Drug-Likeness					Water Solubility		Pharmacokinetics	
	MW (g/mol)	Fsp^3^	RB	HBA	HBD	MR	tPSA	ilogP	XlogP3	WlogP	MlogP	SILICOS-IT	Consensus logP	Lipinski	Ghose	Veber	Egan	Muegge	ESOL	Class	logKp (cm/s)	F
**4a**	210.29	0.00	1	0	0	67.26	28.24	2.74	4.97	4.57	4.13	5.27	4.34	0	0	0	0	1	−4.95	moderately	−4.05	0.55
**5a**	314.40	0.00	3	1	0	97.14	45.31	3.19	5.94	5.80	4.43	6.66	5.20	1	1	0	0	1	−6.01	poorly	−4.00	0.55
**4b**	255.29	0.00	2	2	0	76.08	74.06	2.45	4.80	4.48	3.54	2.99	3.65	0	0	0	0	0	−4.93	moderately	−4.45	0.55
**5b**	404.40	0.00	5	5	0	114.78	136.95	1.67	5.60	5.62	2.30	2.27	3.49	0	1	0	1	1	−6.08	poorly	−4.79	0.55
**4c**	240.32	0.07	2	1	0	73.75	37.47	3.06	4.49	4.58	3.58	5.23	4.28	0	0	0	0	0	−4.96	moderately	−4.26	0.55
**5c**	374.45	0.09	5	3	0	110.12	63.77	3.79	5.89	5.82	3.60	6.73	5.17	0	1	0	0	1	−6.12	poorly	−4.40	0.55
**4d**	235.30	0.00	1	1	0	71.98	52.03	2.70	4.69	4.44	3.24	5.21	4.06	0	0	0	0	0	−4.84	moderately	−4.41	0.55
**5d**	364.42	0.00	3	3	0	106.57	92.89	3.04	5.38	5.54	2.92	6.69	4.71	0	0	0	0	1	−5.87	moderately	−4.70	0.55
**4e**	278.29	0.07	2	3	0	72.26	28.24	3.01	5.86	6.74	5.03	6.19	5.36	1	1	0	1	2	−5.71	moderately	−3.84	0.55
**5e**	450.40	0.09	5	7	0	107.14	45.31	3.65	7.71	10.14	6.03	8.74	7.25	1	1	0	1	1	−7.66	poorly	−3.57	0.55
**4f**	244.74	0.07	1	0	0	72.27	28.24	3.02	5.60	5.22	4.65	5.86	4.87	1	0	0	0	2	−5.51	moderately	−3.82	0.55
**5f**	383.29	0.00	3	1	0	107.16	45.31	3.64	7.20	7.11	5.40	7.91	6.25	1	1	0	1	1	−7.18	poorly	−3.53	0.55
**4g**	224.34	0.07	1	0	0	72.23	28.24	3.01	5.34	4.88	4.39	5.74	4.67	1	0	0	0	2	−5.22	moderately	−3.88	0.55
**5g**	342.45	0.09	3	1	0	107.07	45.31	3.75	6.67	6.42	4.87	7.68	5.88	1	1	0	1	1	−6.59	poorly	−3.65	0.55
**4h**	225.31	0.00	1	0	1	71.67	54.26	2.38	4.29	4.16	3.33	4.50	3.73	0	0	0	0	0	−4.57	moderately	−4.63	0.55
**5h**	344.43	0.00	3	1	2	105.94	97.35	2.53	4.58	4.98	3.16	5.19	4.09	0	0	0	0	0	−5.28	moderately	−5.15	0.55
**4i**	226.29	0.00	1	1	1	69.28	48.47	2.38	4.62	4.27	3.33	4.74	3.87	0	0	0	0	0	−4.78	moderately	−4.40	0.55
**5i**	346.40	0.00	3	3	2	101.18	85.77	2.53	5.23	5.21	3.16	5.67	4.36	0	0	0	0	1	−5.71	moderately	−4.70	0.55
**Galantamine**	287.35	0.53	1	4	1	84.05	41.93	2.66	1.84	1.32	1.74	2.03	1.92	0	0	0	0	0	−2.93	soluble	−6.75	0.55

Molecular weight: MW, topological polar surface area: tPSA, Molar Refractivity: MR, fraction of sp3 carbon atoms: Fsp3, HBD: hydrogen bonds donor, HBA: hydrogen bond acceptor, RB: rotatable bonds, LogP values: indicator of Lipophilicity, ESOL: aqueous solubility parameter, Log Kp: skin permeation, F: Bioavailability Score.
