# Supplementary material for: A New Class of Benzo[b]thiophene-chalcones as Cholinesterase Inhibitors: Synthesis, Biological Evaluation, Molecular Docking and ADME Studies
Source: Molecules. 2024 Aug 7;29(16):3748. doi: 10.3390/molecules29163748 (PMC11356821; doi:10.3390/molecules29163748)
Supplement: Supplementary file 1 [file molecules-29-03748-s001.zip › molecules-3137749-supplementary.pdf]

Supplementary Material

Table of Contents

1. **General Experimental Information** S3

1.1 General procedure for the preparation of 2-mercaptobenzyltriphenylphosphonium bromide **2** S4

1.2 General procedure for the preparation of 2-phenylbenzothiophenes **4a-g** and 3-benzoyl-2-phenylbenzothiophenes **5a-g** S4

1.3 2-Phenylbenzothiophene **4a** S4

1.4 3-Benzoyl-2-phenylbenzothiophene **5a** S5

1.5 2-(4-Nitrophenyl)benzothiophene **4b** S5

1.6 3-(4-Nitrobenzoyl)-2-(4-nitrophenyl)benzothiophene **5b** S6

1.7 2-(4-Methoxyphenyl)benzothiophene **4c** S6

1.8 3-(4-Methoxybenzoyl)-2-(4-methoxyphenyl)benzothiophene **5c** S6

1.9 4-(Benzothiophene-2-yl)benzonitrile **4d** S7

1.10 4-Cianophenyl-[2-(4-cianophenyl-benzothiophen-3-yl)-methanone **5d** S7

1.11 2-(4-Trifluoromethylphenyl)benzothiophene **4e** S8

1.12 3-(4-Trifluoromethylbenzoyl)-2-(4-trifluoromethylphenyl)benzothiophene **5e** S8

1.13 2-(4-Chlorophenyl)benzothiophene **4f** S8

1.14 3-(4-Chlorobenzoyl)-2-(4-chlorophenyl)benzothiophene **5f** S9

1.15 2-(4-Methylphenyl)benzothiophene **4g** S9

1.16 3-(4-Methylbenzoyl)-2-(4-methylphenyl)benzothiophene **5g** S10

1.17 Procedure for the preparation of 2-(4-aminophenyl)benzothiophene **4h** S10

1.18 Procedure for the preparation of 3-(4-aminobenzoyl)-2-(4-aminophenyl)benzothiophene **5h** S11

1.19 Procedure for the preparation of 2-(4-hydroxyphenyl)benzothiophene **4i** S11

1.20 Procedure for the preparation of 3-(4-hydroxybenzoyl)-2-(4-hydroxyphenyl)benzothiophene **5i** S12

2 **NMR Spectra** S14

3 **Biological activity** S34

4 **References** S36

1. General Experimental Information

Starting materials, solvent and reagents were obtained from commercial suppliers (Sigma-Aldrich) and were used without further purification. All reactions were performed under N_2 atmosphere. Analytical thin layer chromatography (TLC) was carried out on silica gel 60 F254 plates (0.25 mm), visualized by exposure to UV light (254 nm). Column chromatography purifications were performed using. Aldrich silica gel (60-120) mesh size. Melting points were determined on a Stuart Scientific SMP 11 melting point apparatus and are uncorrected. Concentration and evaporation of the solvent after reaction or extraction were carried out on a rotary evaporator (Büchi Rotavapor) operating at reduced pressure. ^{1}H NMR and ^{13}C NMR spectra were recorded with a spectrometer (Varian INOVA) operating at a field of 14.4 T (600 MHz for ^{1}H, 150.8 MHz for ^{13}C) and using $CDCl_3$ as solvent. Chemical shifts are reported in ppm (δ) relative to TMS (tetramethylsilane) as an internal standard. The 150.8 MHz ^{13}C spectra were acquired under proton decoupling conditions with a 36000 Hz spectral width, 5.5 µs (60° tip angle) pulse width, 1 s acquisition time and 4 s delay time. The long relaxation time was needed to observe some quaternary carbons. Coupling constants *J* are expressed in hertz (Hz). Spin multiplicities are given as s (singlet), d (doublet), dd (doublet of doublets), m (multiplet) and apparent triplet (app t). GC-MS: low resolution mass spectrometric experiments were carried out on a Saturn 2000 ion-trap coupled with a Varian 3800 gas chromatograph (Varian, Walnut Creek, CA) operating under EI conditions (electron energy 70 eV, emission current 20 mA, ion-trap temperature 200 °C, manifold temperature 80 °C, automatic gain control (AGC) target 21.000) with the ion trap operating in scan mode (scan range from m/z 40-600 at a scan rate of 1 scan/s). Aliquots of 1 µL of solutions 1.0 x 10^{-5} M in dichloromethane (DCM) have been introduced into the gas chromatographer inlet. An Agilent J&W VF-5ms Low-bleed/MS GC capillary column (30 m, 0.25 mm i.d., 0.25 mm film thickness) (Agilent Technologies Inc., Wilmington, DE, USA), was used. The oven temperature was programmed from 100°C (held for 2 min) to 325 °C at 30 °C/min (held for 10 min). The temperature was then ramped to 350 at 20 °C/min. The transfer line was maintained at 250 °C and the injector port (30:1 split) at 290 °C. HRMS: positive ESI-MS spectra were recorded with

a high-resolution LTQ Orbitrap Elite™ mass spectrometer (Thermo Fisher Scientific). The solutions were infused at a flow rate of 5.00 µL/min into the ESI source. Spectra were recorded in the range of m/z 100-1500 with a resolution of 240000. The instrumental conditions were as follows. Spray voltage 3500 V, capillary temperature 275 °C, sheath gas 5-10 (arbitrary units), auxiliary gas 3 (arbitrary units), sweep gas 0 (arbitrary units), probe heater temperature 50 °C.

1.1 ***General procedure for the preparation of 2-mercaptobenzyltriphenylphosphonium bromide (2)***

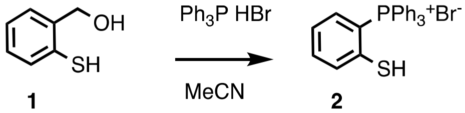

A mixture of 2-mercaptobenzyl alcohol **1** (1.0 equiv.) and triphenylphosphonium bromide ($PPh_3 \cdot HBr$) (1.0 equiv.) in acetonitrile anhydrous (MeCN) (50 mL) was stirred under reflux for 2 h. The solid formed was filtered and washed with CH_3CN to give the desired compounds **2** as white solid. Yield: 98 %; mp: 210-212 °C.[1,2]

1.2 ***General procedure for the preparation of 2-phenylbenzothiophenes 4a-g and 3-benzoyl-2-phenylbenzothiophenes 5a-g***

A mixture of 2-mercaptobenzyltriphenylphosphonium bromide (**2**) (1.0 equiv.) and the proper benzoyl chloride **3a-g** (3.0 equiv.) were dissolved in toluene (40 mL). Triethylamine (Et_3N) (3 equiv.) was added dropwise and the reaction mixture stirred under reflux for 2 h. The precipitate was removed by filtration. The filtrate was concentrated, and the mixture containing the two reaction products was purified by silica gel chromatography (petroleum ether/ethyl acetate) to give the pure compounds **4a-g**[3-5] and **5a-g**.[6,7]

1.3 ***2-Phenylbenzothiophene (4a)***

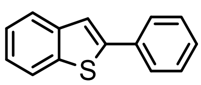

Accordingly to procedure, using phosphonium salt **2** (0.51 g, 1.1 mmol) and benzoyl chloride **3a** (0.61 g, 3.3 mmol), Et_3N (0.45 mL) and toluene (40 mL). Purification by column chromatography on silica gel in petroleum ether/ethyl acetate 10:0.5 for **4a** and 10:1 for **5a**.

4a was obtained as a white solid: (0.13 g, 56% yield); mp: 171-173 °C; ^{1}H NMR (600 MHz, $CDCl_3$): δ = 7.84 – 7.77 (m, 2H), 7.75 – 7.71 (m, 2H), 7.56 (s, 1H), 7.46 – 7.42 (m, 2H), 7.38 – 7.30 (m, 3H) ppm; ^{13}C NMR (151 MHz, $CDCl_3$): δ = 144.3, 140.7, 139.5, 134.3, 129.3, 128.9, 128.7, 128.3, 126.5, 124.5, 124.3, 123.6, 122.3, 119.5 ppm; MS (EI, 70eV) *m/z* (%): 210 (100) [$M^{+\cdot}$], 165 (10); HRMS (ESI): calcd. for $C_{14}H_{11}S$ [M+H]+ = 211.0501; found 211.0504.[3-5]

1.4 ***3-Benzoyl-2-phenylbenzothiophene (5a)***

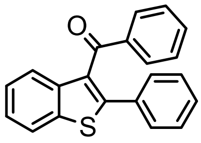

5a was obtained as a white solid: (0.09 g, 28% yield); mp: 106-108 °C; ^{1}HNMR (600 MHz, $CDCl_3$): δ = 7.95 – 7.89 (m, 1H), 7.86 – 7.76 (m, 3H), 7.58 – 7.37 (m, 5H), 7.35 – 7.19 (m, 5H) ppm; ^{13}C NMR (151 MHz, $CDCl_3$): δ = 194.4, 146.6, 139.8, 139.1, 137.6, 133.3, 131.6, 130.1, 129.5, 128.9, 128.7, 128.4, 125.3, 125.2, 123.8, 122.1 ppm; MS (EI, 70eV) *m/z* (%): 314 (100) [$M^{+\cdot}$], 313 (45), 237 (60), 208 (12), 165 (16), 105 (12), 77 (22); HRMS (ESI): calcd. for $C_{21}H_{15}OS$ [M+H]+ = 315.0844; found 315.0847.[6]

1.5 ***2-(4-Nitrophenyl)benzothiophene (4b)***

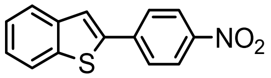

Accordingly to procedure, using phosphonium salt **2** (0.03 g, 1.1 mmol) and benzoyl chloride **3b** (0.61 g, 3.3 mmol), Et_3N (0.45 mL) and toluene (40 mL). Purification by column chromatography on silica gel in petroleum ether/ethyl acetate 10:0.5 for **4b** and 10:1 for **5b**.

4b was obtained as a yellow solid: (0.03 g, 12% yield); mp: 201-202 °C; ^{1}HNMR (600 MHz, $CDCl_3$): δ = 8.28 (d, 2H, *J* = 8.8 Hz), 7.89 – 7.82 (m, 4H), 7.72 (s, 1H), 7.43 – 7.36 (m, 2H) ppm; ^{13}C NMR (151 MHz, $CDCl_3$): δ = 147.3, 141.3, 140.7, 140.4, 140.3, 130.0, 126.9, 125.7, 125.2, 124.5, 124.4, 124.0 ppm; MS (EI, 70eV) *m/z* (%): 255 (100) [$M^{+\cdot}$], 239 (8), 225 (32), 208 (24), 197 (12), 165 (21); HRMS (ESI): calcd. for $C_{14}H_{10}NO_2S$ [M+H]+ = 256.0432; found 256.0435.[3,5]

1.6 ***3-(4-Nitrobenzoyl)-2-(4-nitrophenyl)benzothiophene (5b)***

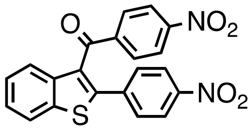

5b was obtained as a yellow solid: (0.27 g, 63% yield); mp: 214-216 °C; ^{1}H NMR (600 MHz, $CDCl_3$): δ = 8.35 – 8.23 (m, 3H), 8.00 – 7.88 (m, 1H), 7.88 – 7.79 (m, 5H), 7.70 (s, 1H), 7.43 – 7.36 (m, 2H) ppm; ^{13}C NMR (151 MHz, $CDCl_3$): δ 191.8, 150.6, 148.0, 144.5, 141.8, 139.5, 139.3, 139.0, 132.6, 130.8, 130.2, 126.5, 126.2, 124.2, 124.0, 123.9, 122.5 ppm; MS (EI, 70eV) *m/z* (%): 404 (100) [$M^{+\cdot}$], 358 (19), 282 862), 236 821, 208 (11), 104 (6); HRMS (ESI): calcd. for $C_{21}H_{13}N_2O_5S$ [M+H]+ = 405.0545; found 405.0548.

1.7 ***2-(4-Methoxyphenyl)benzothiophene (4c)***

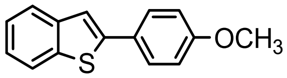

Accordingly to procedure, using phosphonium salt **2** (0.055 g, 1.1 mmol) and benzoyl chloride **3c** (0.56 g, 3.3 mmol), Et_3N (0.45 mL) and toluene (40 mL). Purification by column chromatography on silica gel in petroleum ether/ethyl acetate 10:1 for **4c** and 10:2 for **5c**.

4c was obtained as a white solid: (0.06 g, 21% yield); mp: 186-188 °C; ^{1}HNMR (600 MHz, $CDCl_3$): δ = 7.84 (d, 1H, *J* = 8.4 Hz), 7.80 (t, 1H, *J* = 7.8, 1.5, Hz), 7.73 (d, 2H, *J* = 8.8 Hz), 7.64 (m, 1H), 7.49 – 7.36 (m, 2H), 6.96 (d, 2H, *J* = 8.8 Hz), 3.87 (s, 3H, OCH_3) ppm; ^{13}C NMR (151 MHz, $CDCl_3$): δ = 160.1, 141.1, 138.1, 136.4, 130.7, 127.9, 125.4, 125.1, 122.2, 118.4, 114.3, 55.5 ppm; MS (EI,

70eV): *m/z* (%): 240 (100) [$M^{+\cdot}$], 225 (45), 197 (28), 165 (9); HRMS (ESI): calcd. for $C_{15}H_{13}OS$ [M+H]+ = 241.0687; found 241.0691.[3-5]

1.8 ***3-(4-Methoxybenzoyl)-2-(4-methoxyphenyl)benzothiophene (5c)***

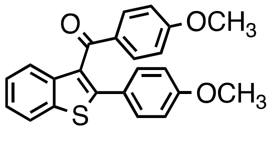

5c was obtained as a white solid: (0.04 g, 9% yield); mp: 124-126 °C; ^{1}H NMR (600 MHz, $CDCl_3$): δ = 7.79 – 7.75 (m, 1H), 7.70 (d, 2H, *J* = 8.8 Hz), 7.59 – 7.52 (m, 1H), 7.31 (d, 2H, *J* = 8.7 Hz), 7.28 – 7.22 (m, 2H), 6.69 (dd, 4H, *J* = 8.8, 2.1 Hz), 3.70 (s, 3H, OCH_3), 3.66 (s, 3H, OCH_3) ppm; ^{13}C NMR (151 MHz, $CDCl_3$): δ = 193.4, 163.9, 160.1, 145.0, 140.1, 138.8, 134.1, 132.5, 131.1, 130.7, 130.5, 125.9, 125.1, 124.8, 123.4, 122.0, 114.3, 113.8, 55.5, 55.4 ppm; MS (EI, 70eV) *m/z* (%): 374 (100) [$M^{+\cdot}$], 267 (8), 225 (15), 195 (12), 135 (31); HRMS (ESI): calcd. for $C_{23}H_{19}O_3S$ [M+H]+ = 375.1055; found 375.1059.[7]

1.9 ***4-(Benzothiophene-2-yl)benzonitrile (4d)***

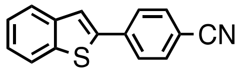

Accordingly to procedure, using phosphonium salt **2** (0.055 g, 1.1 mmol) and benzoyl chloride **3d** (0.55 g, 3.3 mmol), Et_3N (0.45 mL) and toluene (40 mL). Purification by column chromatography on silica gel in petroleum ether/ethyl acetate 9:1 for **4d** and 8:2 for **5d**.

4d was obtained as a white solid: (0.07 g, 25% yield); mp: 184-186 °C; ^{1}H NMR (600 MHz, $CDCl_3$): δ = 7.85 (d, 1H, *J* = 8.5 Hz), 7.81 – 7.75 (m, 3H), 7.70 (d, 2H, *J* = 8.1 Hz), 7.66 (s, 1H), 7.40 – 7.35 (m, 2H,) ppm; ^{13}C NMR (151 MHz, $CDCl_3$): δ = 140.4, 138.8, 132.9, 132.6, 129.9, 126.9, 126.5, 125.5, 125.1, 124.3, 122.6, 121.9, 118.8, 111.6 ppm; MS (EI, 70eV) *m/z* (%): 235 (100) [$M^{+\cdot}$], 203, 190 (5); HRMS (ESI): calcd. for $C_{15}H_{10}NS$ [M+H]+ = 236.0534; found 236.0538.[4,5]

1.10 ***4-Cianophenyl-[2-(4-cianophenyl)-benzothiophen-3-yl]-methanone (5d)***

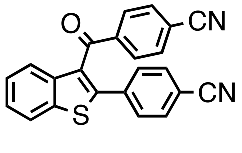

5d was obtained as a white solid: (0.2 g, 50% yield); mp: 198-200 °C; ^{1}H NMR (600 MHz, $CDCl_3$): δ = 7.93 (d, 1H, *J* = 7.2 Hz), 7.83 (d, 2H, *J* = 8.4 Hz), 7.71 (d, 1H, *J* = 8.1 Hz), 7.60 (d, 2H, *J* = 8.4 Hz), 7.54 (d, 2H, *J* = 8.2 Hz), 7.52 – 7.40 (m, 4H) ppm; ^{13}C NMR (151 MHz, $CDCl_3$): δ = 192.1, 144.9, 140.4, 139.4, 139.1, 137.5, 132.6, 132.5, 130.2, 130.0, 126.4, 126.1, 123.9, 122.4, 118.1, 117.7, 117.0, 113.0 ppm; MS (EI, 70eV) *m/z* (%) 364 (100) $[M^{+\cdot}]$, 262 (58), 130 (6), 102 (10); HRMS (ESI): calcd. for $C_{23}H_{13}N_2OS$ [M+H]+ = 365.0748; found 365.0753.

1.11 ***2-(4-Trifluoromethylphenyl)benzothiophene*** (*4e)*

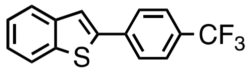

Accordingly to procedure, using phosphonium salt **2** (0.055 g, 1.1 mmol) and benzoyl chloride **3e** (0.55 g, 3.3 mmol), Et_3N (0.45 mL) and toluene (40 mL). Purification by column chromatography on silica gel in petroleum ether/ethyl acetate 9.8:0.2 for **4e** and 9:1 for **5e**.

4e was obtained as a white solid: (0.04 g, 12% yield); mp: 216-218 °C; ^{1}HNMR (600 MHz, $CDCl_3$): δ = 7.95 – 7.88 (m, 1H), 7.87 – 7.78 (m, 3H), 7.77 – 7.66 (m, 2H~~, *J* = 8.5 Hz~~), 7.63 (s, 1H), 7.55 – 7.35 (m, 2H) ppm; ^{13}C NMR (151 MHz, $CDCl_3$): δ = 142.4, 140.6, 139.9, 129.7 (Cq, q, $J_{Cq\text{-}F}$ = 32.5 Hz), 126.7, 126.1, 125.5 (CH, q, $J_{CH\text{-}F}$ = 3.8 Hz), 125.1, 124.9, 124.1(CF_3, q, $J_{C\text{-}F}$ = 270.3 Hz), 122.7, 122.5, 121.2 ppm; MS (EI, 70eV) *m/z* (%): 278 (100) $[M^{+\cdot}]$, 260 (4), 130 (6), 102 (10); ^{19}F NMR (565 MHz, $CDCl_3$): δ = -62.62(s) ppm; HRMS (ESI): calcd. for $C_{15}H_{10}F_3S$ [M+H]+ = 279.0455; found 279.0459.[4]

1.12 ***3-(4-Trifluoromethylbenzoyl)-2-(4-trifluoromethylphenil)benzothiophene (5e)***

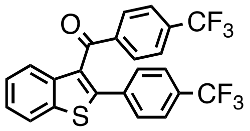

5e was obtained as a white solid: (0.2 g, 38% yield); mp: 92-95 °C; ^{1}HNMR (600 MHz, $CDCl_3$): δ = 7.92 (t, 1H, *J* = 7.7 Hz), 7.85 (d, 1H, *J* = 7.8 Hz), 7.84 – 7.79 (m, 3H), 7.76 – 7.71 (m, 1H), 7.67 (t, 2H, *J* = 8.2 Hz), 7.63 (s, 1H), 7.52 – 7.43 (m, 1H), 7.42 – 7.33 (m, 2H) ppm; ^{13}C NMR (151 MHz, $CDCl_3$): δ = 192.7 (CO), 145.8, 140.3, 139.3, 139.2, 136.7, 134.8, 134.6, 132.2 (Cq, q, $J_{Cq\text{-}F}$ = 33.2 Hz),, 131.2 (Cq, q, $J_{Cq\text{-}F}$ = 33.1 Hz),, 131.02 130.2, 129.9 (CH, q, $J_{CH\text{-}F}$ = 3.0 Hz), 126.03, 125.9, 125.8, 125.6 (CH, q, $J_{CH\text{-}F}$ = 3.0 Hz), 124.0 (CF_3, q, $J_{C\text{-}F}$ = 273.3 Hz), 122.3 (CF_3, q, $J_{C\text{-}F}$ = 271.8 Hz) ppm; MS (EI, 70eV) *m/z* (%): 450 (100) [$M^{+\cdot}$], 381 (19), 305 (93), 173 (18); ^{19}F NMR (565 MHz, $CDCl_3$): δ = -63.36 (s), 63.06 (s) ppm; HRMS (ESI): calcd. for $C_{23}H_{13}F_6OS$ [M+H]+ = 451.0591; found 451.0583.

1.13 ***2-(4-Chlorophenyl)benzothiophene*** (***4f***)

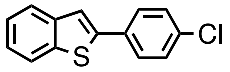

Accordingly to procedure, using phosphonium salt **2** (0.055 g, 1.1 mmol) and benzoyl chloride **3f** (0.55 g, 3.3 mmol), Et_3N (0.45 mL) and toluene (40 mL). Purification by column chromatography on silica gel in petroleum ether/ethyl acetate 9.8:0.2 for **4f** and 9:1 for **5f**.

4f was obtained as a white solid: (0.08 g, 31% yield); mp: 191-193 °C; ^{1}HNMR (600 MHz, $CDCl_3$): δ = 7.80 (d, 1H, *J* =7.6 Hz), 7.65 – 7.57 (m, 2H), 7.36 – 7.23 (m, 3H), 7.20 – 7.12 (m, 3H) ppm; ^{13}C NMR (151 MHz, $CDCl_3$): δ = 142.9, 140.7, 139.7, 133.0, 130.6, 125.9, 125.4, 124.8, 124.7, 123.8, 122.4, 120.0, 110.5 ppm; MS (EI, 70eV):*m/z* (%): 244 (100) [$M^{+\cdot}$(^{35}Cl)], 246 (40) [M+2 (^{37}Cl)], 208 (10), 165 (11); HRMS (ESI): calcd. for $C_{14}H_{10}ClS$ [M+H]+ = 245.0191; found 245.0195.[3,4]

1.14 ***3-(4-Chlorobenzoyl)-2-(4-chlorophenyl)benzothiophene (5f)***

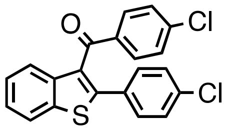

5f was obtained as a white solid: (0.2 g, 47% yield); mp: 87-89 °C; ^{1}H NMR (600 MHz, $CDCl_3$): δ = 7.83 (d, 1H, *J* = 7.9 Hz), 7.77 (d, 1H, *J* = 7.5 Hz), 7.75 – 7.71 (m, 1H), 7.66 – 7.62 (m, 2H), 7.52 (s, 1H), 7.50 – 7.42 (m, 2H), 7.42 – 7.38 (m, 2H), 7.35 (m, 2H) ppm; ^{13}C NMR (151 MHz, $CDCl_3$): δ = 192.9, 145.0, 140.2, 139.5, 139.0, 135.8, 135.4, 131.7, 131.5, 131.3, 130.8, 130.5, 129.1, 129.0, 125.6, 125.5, 123.7, 122.2 ppm; MS (EI, 70eV) *m/z* (%):382 (100) [$M^{+\cdot}$ (^{35}Cl)], 384 (70) [M+2 (^{37}Cl)], 347 (14), 271 (50), 236 (18), 139 (17); HRMS (ESI): calcd. for $C_{21}H_{13}Cl_2OS$ [M+H]+ = 383.0064; found 383.0060.

1.15 ***2-(4-Methylphenyl)benzothiophene*** (***4g***)

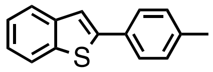

Accordingly to procedure, using phosphonium salt **2** (0.04 g, 1.1 mmol) and benzoyl chloride **3g** (0.55 g, 3.3 mmol), Et_3N (0.45 mL) and toluene (40 mL). Purification by column chromatography on silica gel in petroleum ether/ethyl acetate 9.5:0.5 for **4g** and 9:1 for **5g**.

4g was obtained as a white solid: (0.04 g, 19% yield); mp: 166-168 °C; ^{1}HNMR (600 MHz, $CDCl_3$): δ = 7.73 (d, 1H, *J* = 8.0 Hz), 7.67 (d, 1H, *J* = 7.5 Hz), 7.52 (d, 2H, *J* = 7.5 Hz), 7.41 (s, 1H), 7.23 (d, 2H, *J* = 7.5 Hz), 7.15 (m, 2H), 2.30 (s, 3H) ppm; ^{13}C NMR (151 MHz, $CDCl_3$): δ = 144.6, 140.9, 139.5, 138.4, 131.7, 129.8, 129.5, 129.3, 126.5, 124.6, 124.3, 123.5, 122.4, 118.9, 21.4 ppm; MS (EI, 70eV) *m/z* (%): 224 (100) [$M^{+\cdot}$], 193 (8); HRMS (ESI): calcd. for $C_{15}H_{13}S$ [M+H]+ = 225.0738; found 225.0741.[3,4]

1.16 ***3-(4-Methylbenzoyl)-2-(4-methylphenyl)benzothiophene (5g)***

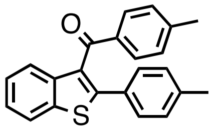

5g was obtained as a white solid: (0.03 g, 9% yield); mp: 156-158 °C; ^{1}H NMR (600 MHz, $CDCl_3$): δ = 7.86 (d, 1H, *J* = 12.0 Hz), 7.71 (d, 2H, *J* = 8.2 Hz), 7.66 – 7.62 (m, 1H), 7.38 – 7.32 (m, 4H), 7.10 (d, 2H, *J* = 8.0 Hz), 7.05 (d, 2H, *J* = 8.0 Hz), 2.33 (s, 3H, CH_3), 2.28 (s, 3H, CH_3) ppm; ^{13}C NMR

(151 MHz, $CDCl_3$): δ = 194.4, 145.7, 144.4, 139.9, 138.9, 135.1, 131.7, 131.5, 130.6, 130.2, 129.8, 129.5, 129.2, 125.4, 125.1, 124.9, 123.6, 122.1, 21.8, 21.3 ppm; MS (EI, 70eV): *m/z* (%): 342 (100) [$M^{+\cdot}$], 327 (18), 251 (34), 119 (15), 91(20); HRMS (ESI): calcd. for $C_{23}H_{19}OS$ [M+H]+ = 343.1156; found 343.1160.

1.17 ***Procedure for the preparation of 2-(4-aminophenyl)benzothiophene (4h)***

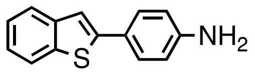

A mixture of 2-(4-nitrophenyl)benzothiophene **4b** (5 mmol), $SnCl_2$ x H_2O (25 mmol) and ethanol (20 mL) was stirred under reflux for 12 h. After solvent removal, the resultant solid was extracted with CH_2Cl_2. The organic phase was dried, filtered and the solvent remove by evaporation. The residue was purified by silica gel chromatography in petroleum ether/ethyl acetate 7:3 to afford the pure compound **4h**: yellow solid; yield: 78 %; mp: 144-145 °C; ^{1}H NMR (600 MHz, $CDCl_3$): δ = 7.79 (d, 1H, *J* = 7.8 Hz), 7.71 (d, 1H, *J* = 7.8 Hz), 7.56 – 7.50 (m, 2H), 7.37 (s, 1H), 7.35 – 7.29 (m, 1H), 6.76 – 6.69 (m, 2H), 3.89 (s, 2H, NH_2) ppm; ^{13}C NMR (151 MHz, $CDCl_3$): δ = 146.9, 145.0, 141.2, 139.1, 127.8, 124.9, 124.5, 123.8, 123.2, 122.3, 117.3, 115.4 ppm; HRMS (ESI): calcd. for $C_{14}H_{12}NS$ $[M+H]^+$ = 226.0690; found 226.0679.[5]

1.18 ***Procedure for the preparation of 3-(4-aminobenzoyl)-2-(4-aminophenyl)benzothiophene (5h)***

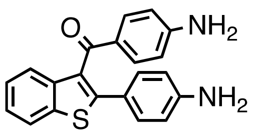

A mixture of 3-(4-nitrobenzoyl)-2-(4-nitrophenyl)benzothiophene **5b** (5 mmol), $SnCl_2$ x H_2O (50 mmol) and ethanol (40 mL) was stirred under reflux for 15 h. After solvent removal, the resultant solid was extracted with CH_2Cl_2. The organic phase was dried, filtered and the solvent remove by evaporation. The residue was purified by silica gel chromatography in petroleum ether/ethyl acetate 3:1, to afford the pure compound **5h**: yellow solid; yield: 80 %; mp: 191-193 °C; ^{1}H NMR (600 MHz,

$CDCl_3$): δ = 8.32 (s,1H), 7-95 – 7.90 (m, 2H), 7.85 (m, 1H), 7.75 – 7.72 (m, 2H), 7.61-7.83 (m, 2H), 6-98 – 6.85 (m, 4H), 4.80 (s, 2H), 4.65 (s, 2H) ppm; ^{13}C NMR (151 MHz, $CDCl_3$): δ = 192.3, 145.1, 140.6, 139.6, 137.7, 132.8, 130.4, 130.2, 126.6, 126.3, 124.2, 122.6, 118.3, 117.4, 117.2, 113.2 ppm; HRMS (ESI): calcd. for $C_{21}H_{17}N_2OS$ $[M+H]^+ = 345.1061$; found 345.1049.

*1.19 **Procedure for the preparation of 2-(4-hydroxyphenyl)benzothiophene (4i)***

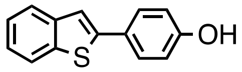

A mixture of 2-(4-methoxyphenyl) benzothiophene **4c** (100 mmol) and pyridine hydrochloride (500 mmol) were placed in a stoppered round bottom flask and subjected to microwave irradiation at 300 W for various time intervals. Reaction progress was monitored by TLC. Microwave irradiation was carried out using a commercial microwave oven operating at variable frequency. After complete conversion the reaction mixture was decomposed using ice-water and extracted with diethyl ether. The ether extract was washed with water, dried over anhydrous sodium sulfate and the solvent removed under reduced pressure. The residue was purified by by silica gel chromatography (petroleum ether/ethyl acetate 7:3) to afford the pure compound **4i**: white solid; yield: 90 %; mp: 160-162 °C; ^1H NMR (600 MHz, $CDCl_3$): δ = 7.81 (d, 1H, J = 8.2 Hz), 7.74 (d, 1H, J = 7.9 Hz), 7.62 – 7.59 (m, 2H), 7.42 (s, 1H), 7.33 (m, 1H), 7.29 (m, 1H), 6.91 – 6.88 (m, 2H) ppm;^{13}C NMR (151 MHz, $CDCl_3$): δ = 155.9, 141.0, 139.3, 128.1, 127.5, 124.6, 124.1, 123.4, 122.3, 118.4, 115.9 ppm; HRMS (ESI): calc. for $C_{14}H_{11}OS$ $[M+H]^+ = 227.053062$; found 227.045434.[8]

*1.20 **Procedure for the preparation of 3-(4-hydroxybenzoyl)-2-(4-hydroxyphenyl)benzothiophene (5i)***

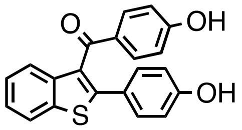

A mixture of 2-(4-methoxyphenyl) benzothiophene **5c** (100 mmol) and pyridine hydrochloride (1000 mmol) were placed in a stoppered round bottom flask and subjected to microwave irradiation at 300

W for various time intervals. Reaction progress was monitored by TLC. Microwave irradiation was carried out using a commercial microwave oven operating at variable frequency. After complete conversion the reaction mixture was decomposed using ice-water and extracted with diethyl ether. The ether extract was washed with water, dried over anhydrous sodium sulfate and the solvent removed under reduced pressure. The residue was purified by by silica gel chromatography (petroleum ether/ethyl acetate 3:2) to afford the pure compound **5i**: white solid; yield: 85 %; mp: 214-216 °C; ^{1}H NMR (600 MHz, $CDCl_3$): δ = 7.87 – 7.77 (m, 3H), 7.69 (d, 2H, *J* = 8.7 Hz), 7.60 (d, *J* = 8.7 Hz, 1H), 7.46 (t, 1H, *J* = 7.6 Hz,), 7.40 (m, 2H), 6.94 (d, 2H, *J* = 8.7 Hz), 6.89 (d,1H, *J* = 8.7 Hz) ppm; ^{13}C NMR (151 MHz, $CDCl_3$): δ = 195.1, 167.7, 156.2, 130.9, 128.15, 125.4, 125.2, 124.6, 124.1, 123.4, 122.6, 122.4, 122.3, 118.4, 115.9, 115.8 ppm; HRMS (ESI): calculated for $C_{21}H_{15}SO_3$ $[M + H]^+$ = 346.0664; found 346.0672.[9]

2. NMR Spectra

Figure S1. ^{1}H NMR spectra of **2-phenylbenzothiophene (4a)**

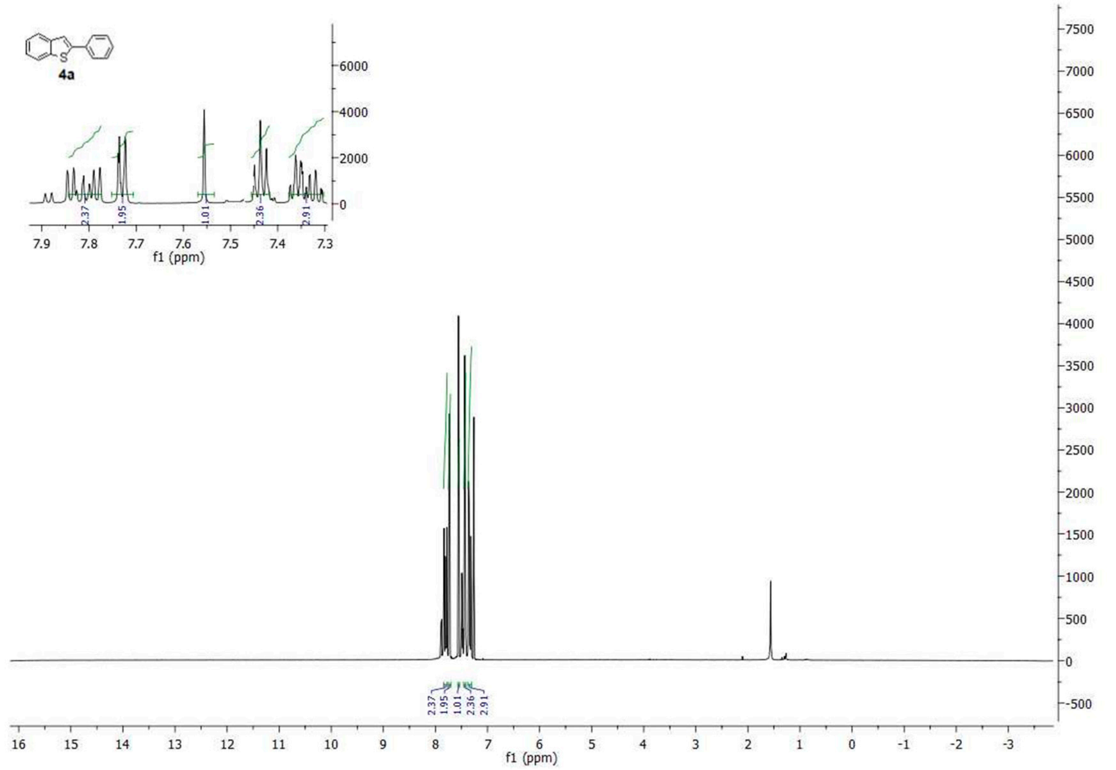

Figure S2. ^{13}C NMR spectra of **2-phenylbenzothiophene (4a)**

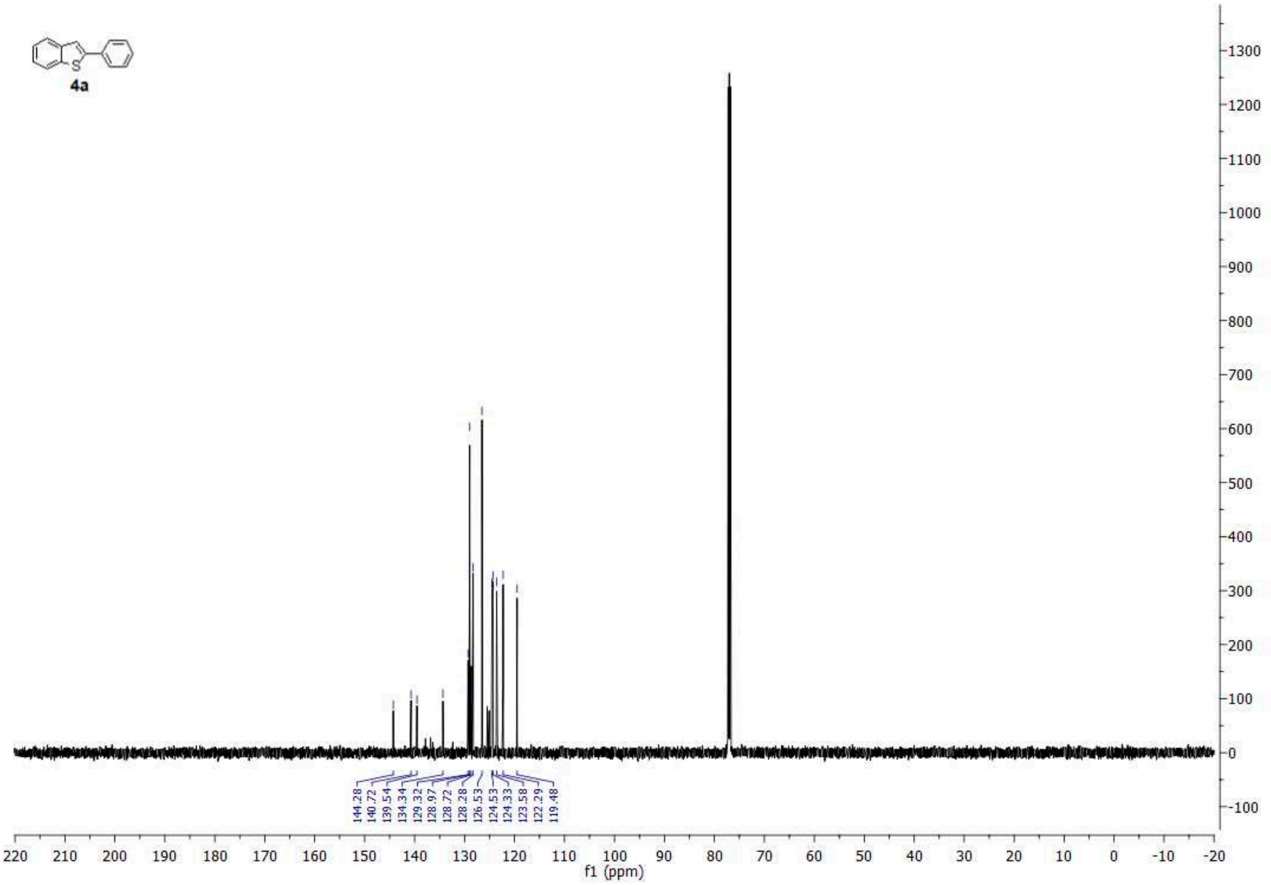

Figure S3.^{1}H NMR spectra of **3-benzoyl-2-phenylbenzothiophene (5a)**

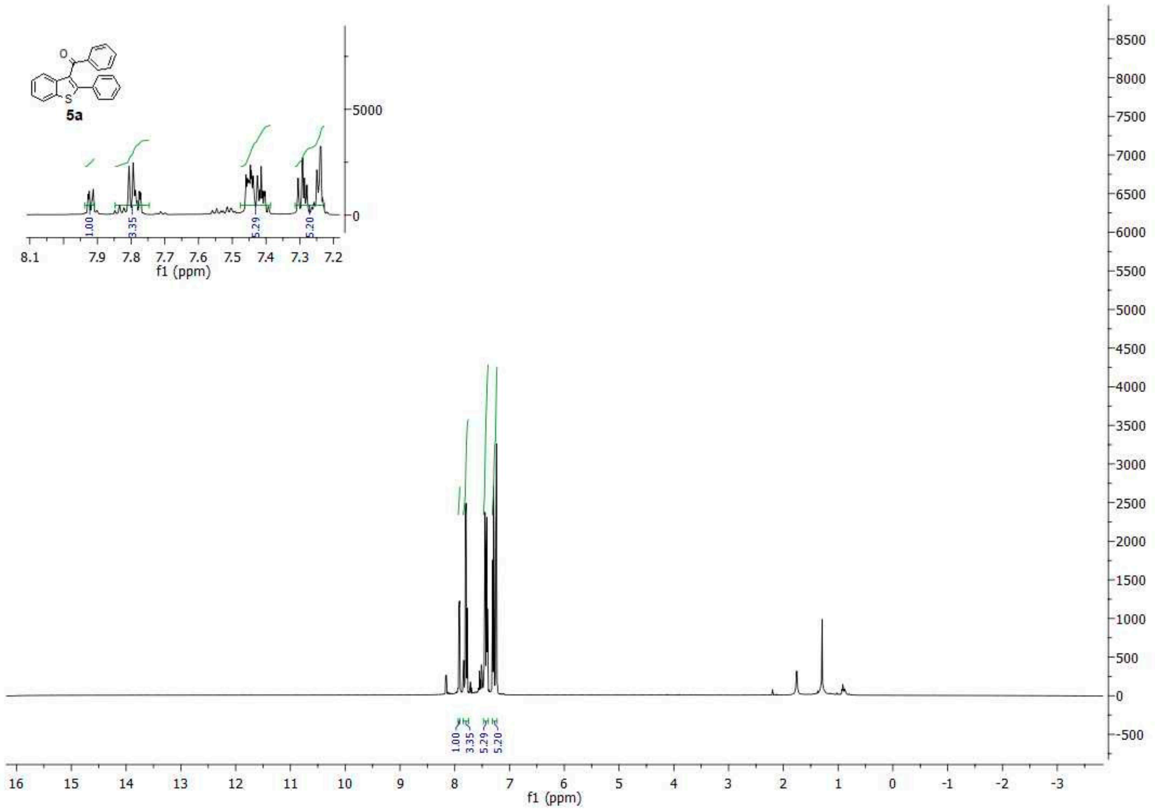

Figure S4.^{13}C NMR spectra of **3-benzoyl-2-phenylbenzothiophene (5a)**

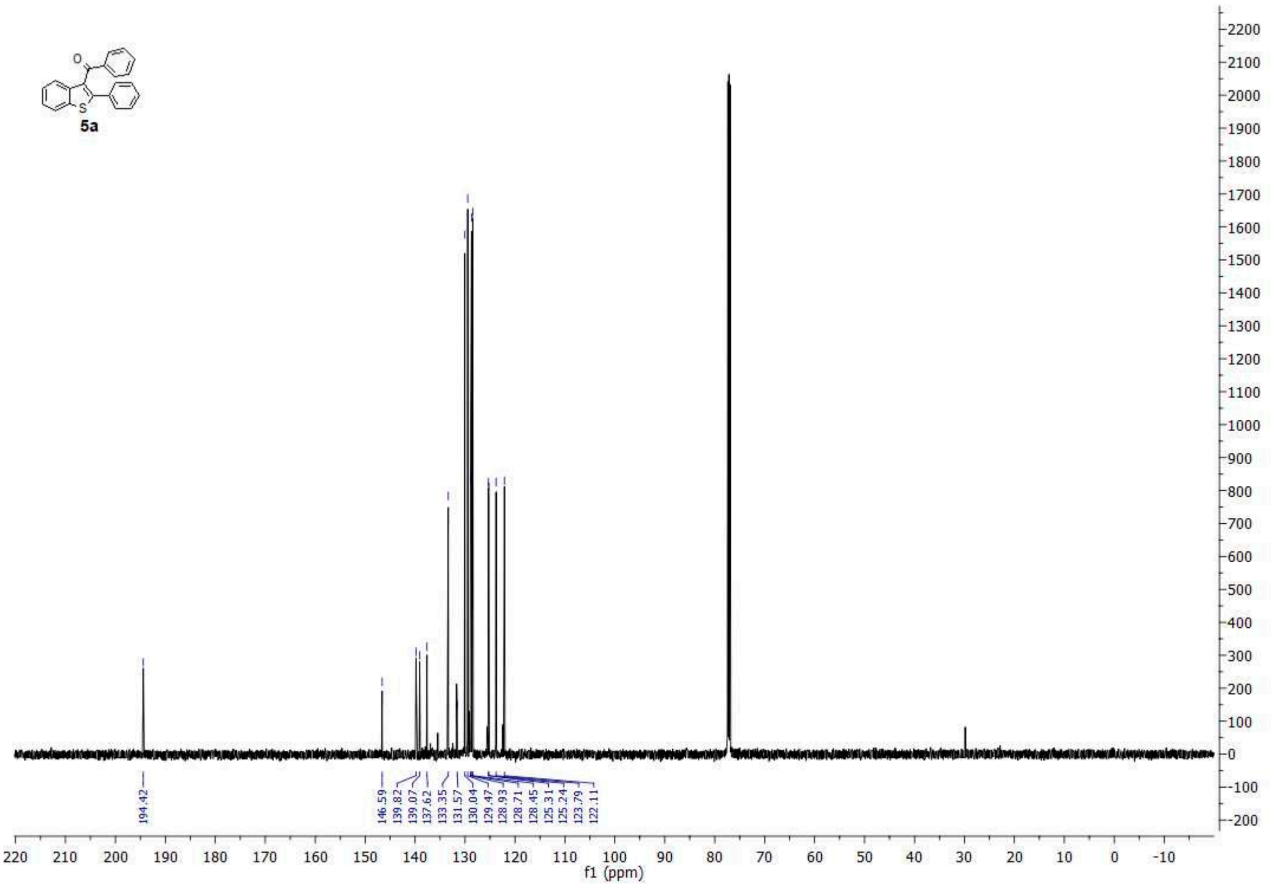

Figure S5.^{1}H NMR spectra of **2-(4-nitrophenyl)benzothiophene (4b)**

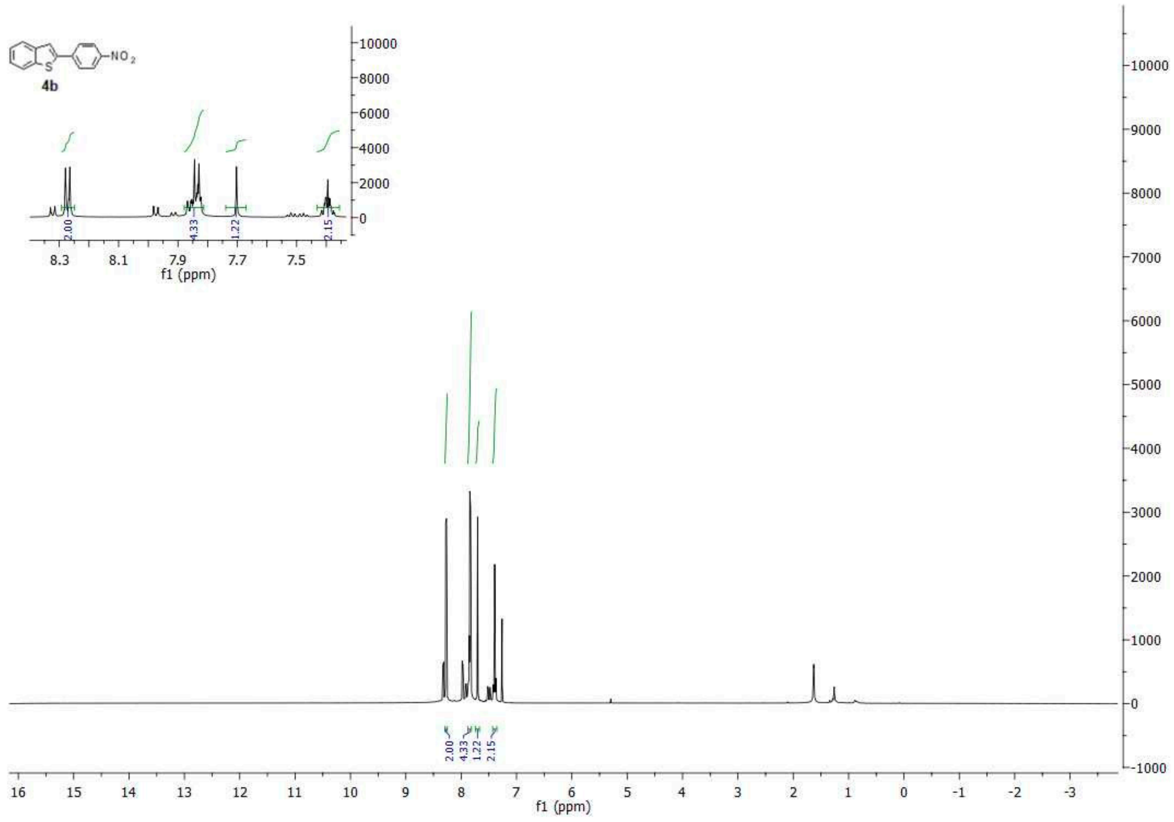

Figure S6.^{13}C NMR spectra of **2-(4-nitrophenyl)benzothiophene (4b)**

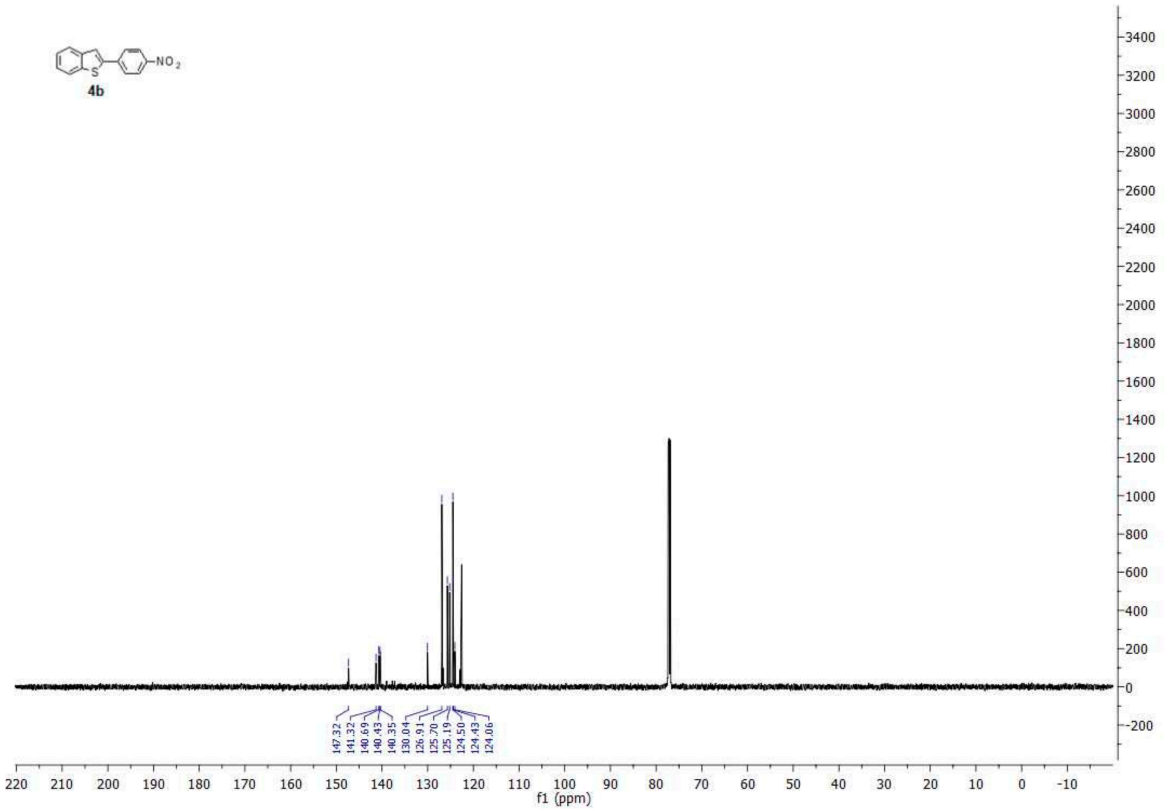

Figure S7.^{1}H NMR spectra of **3-(4-nitrobenzoyl)-2-(4-nitrophenyl)benzothiophene (5b)**

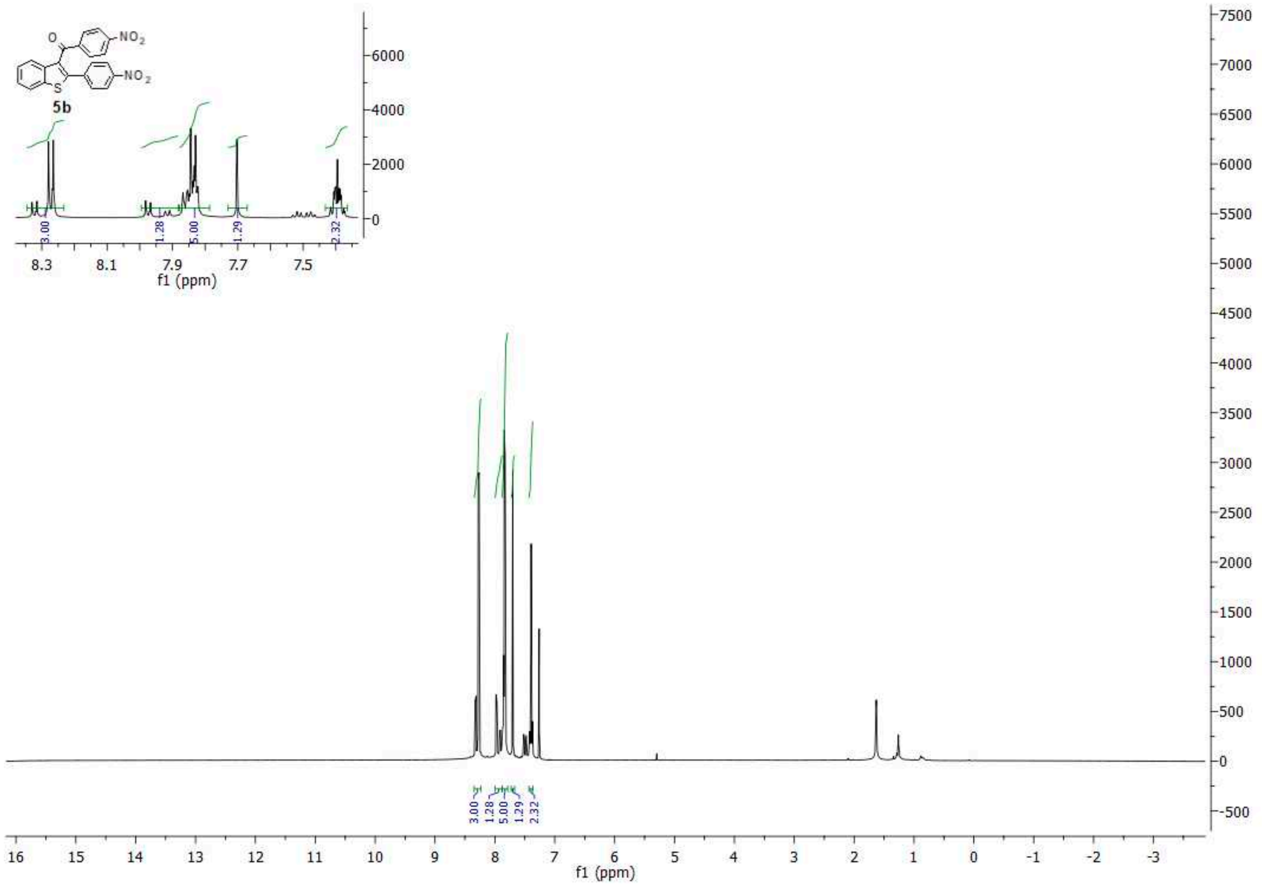

Figure S8.^{13}C NMR spectra of **3-(4-nitrobenzoyl)-2-(4-nitrophenyl)benzothiophene (5b)**

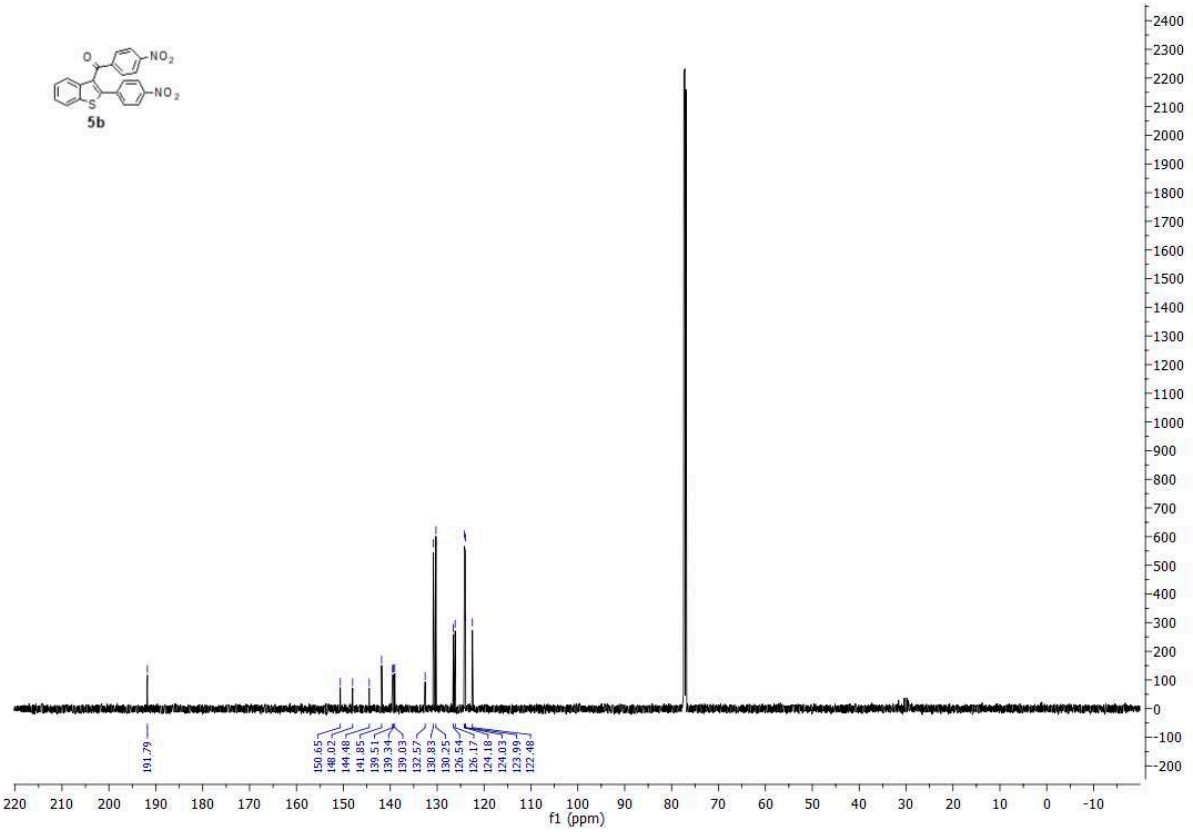

Figure S9.^{1}H NMR spectra of **2-(4-methoxyphenyl)benzothiophene (4c)**

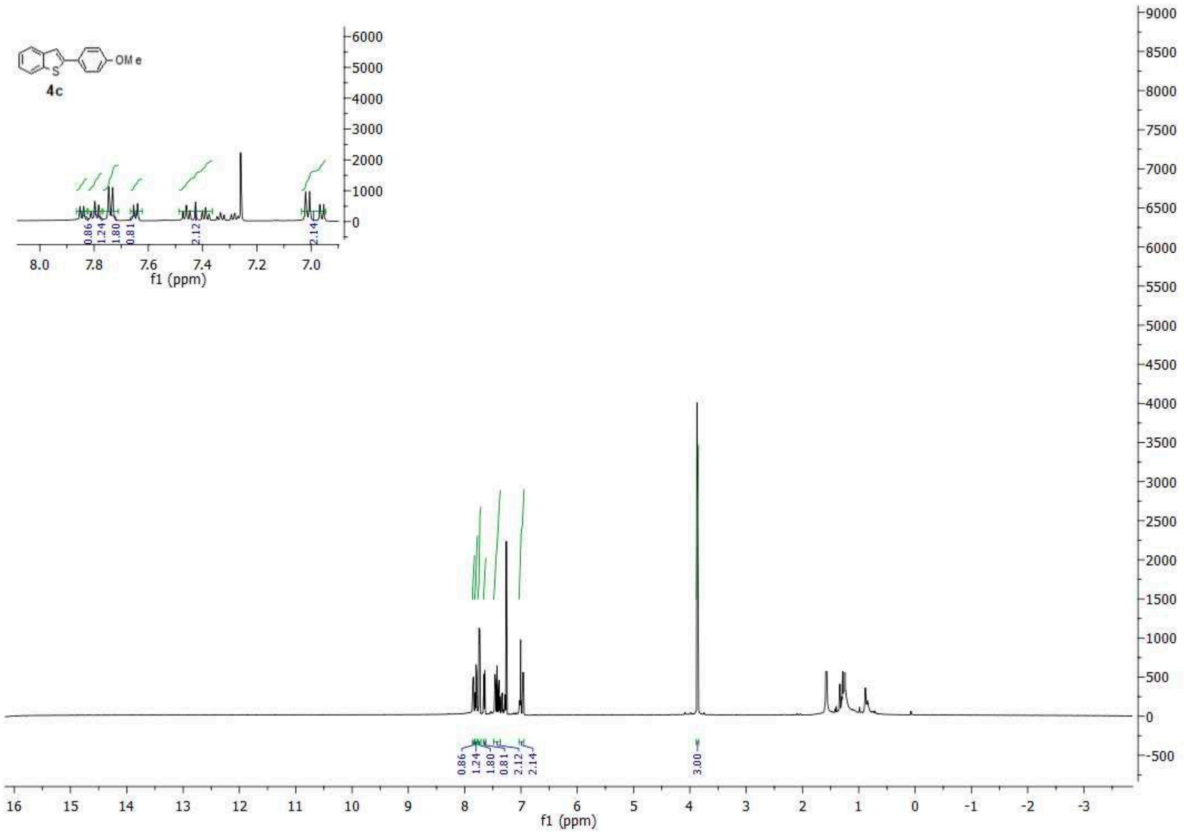

Figure S10.^{13}C NMR spectra of **2-(4-methoxyphenyl)benzothiophene (4c)**

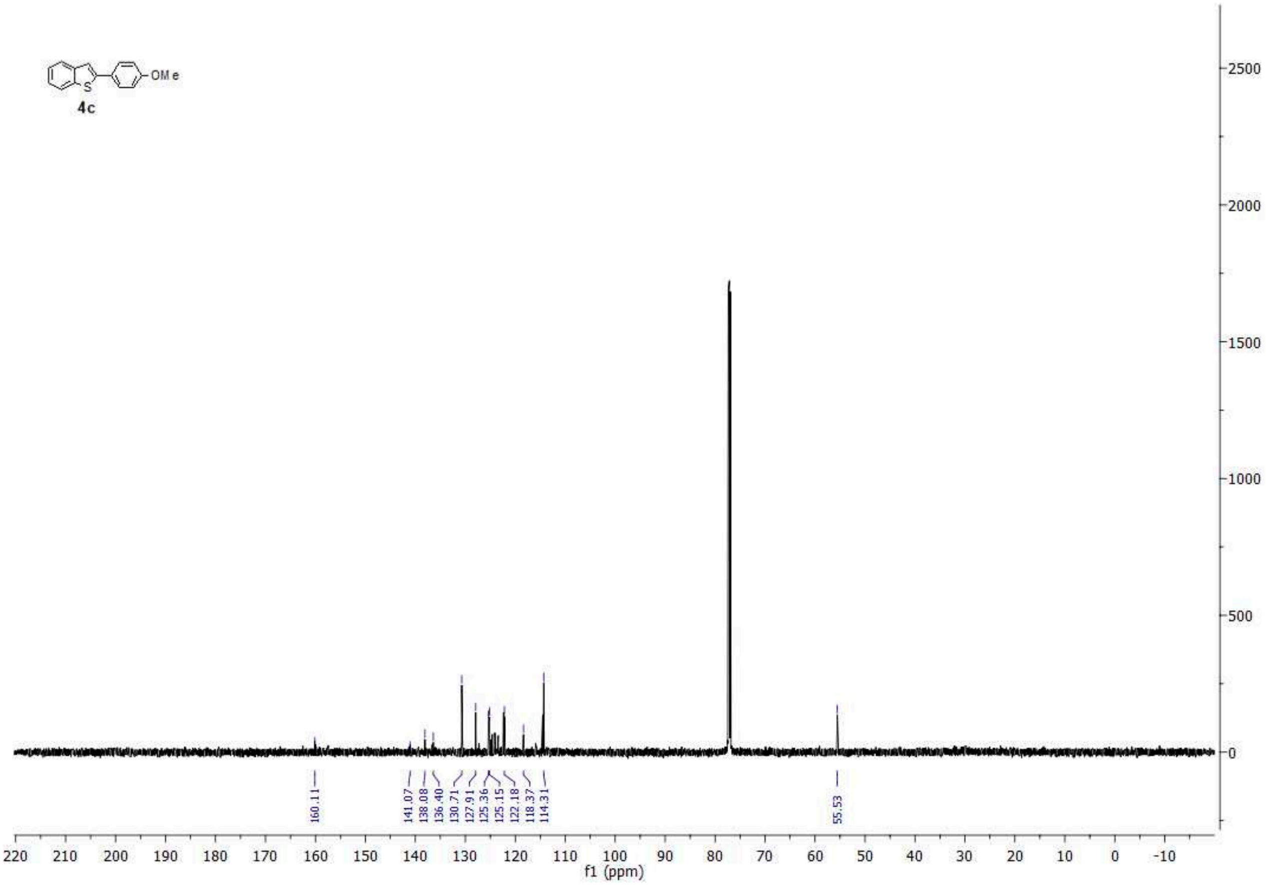

Figure S11.^{1}H NMR spectra of **3-(4-methoxybenzoyl)-2-(4-methoxyphenyl)benzothiophene (5c)**

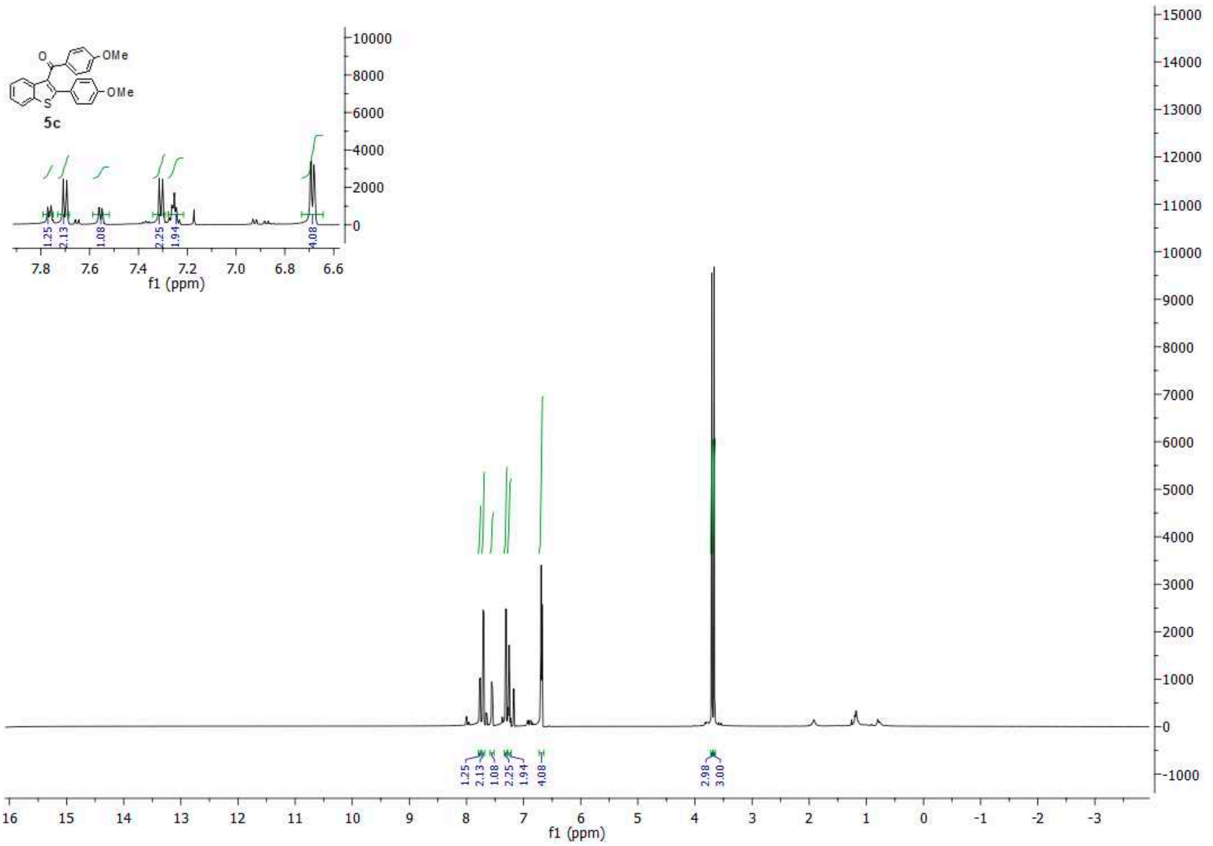

Figure S12.^{13}C NMR spectra of **3-(4-methoxybenzoyl)-2-(4-methoxyphenyl)benzothiophene (5c)**

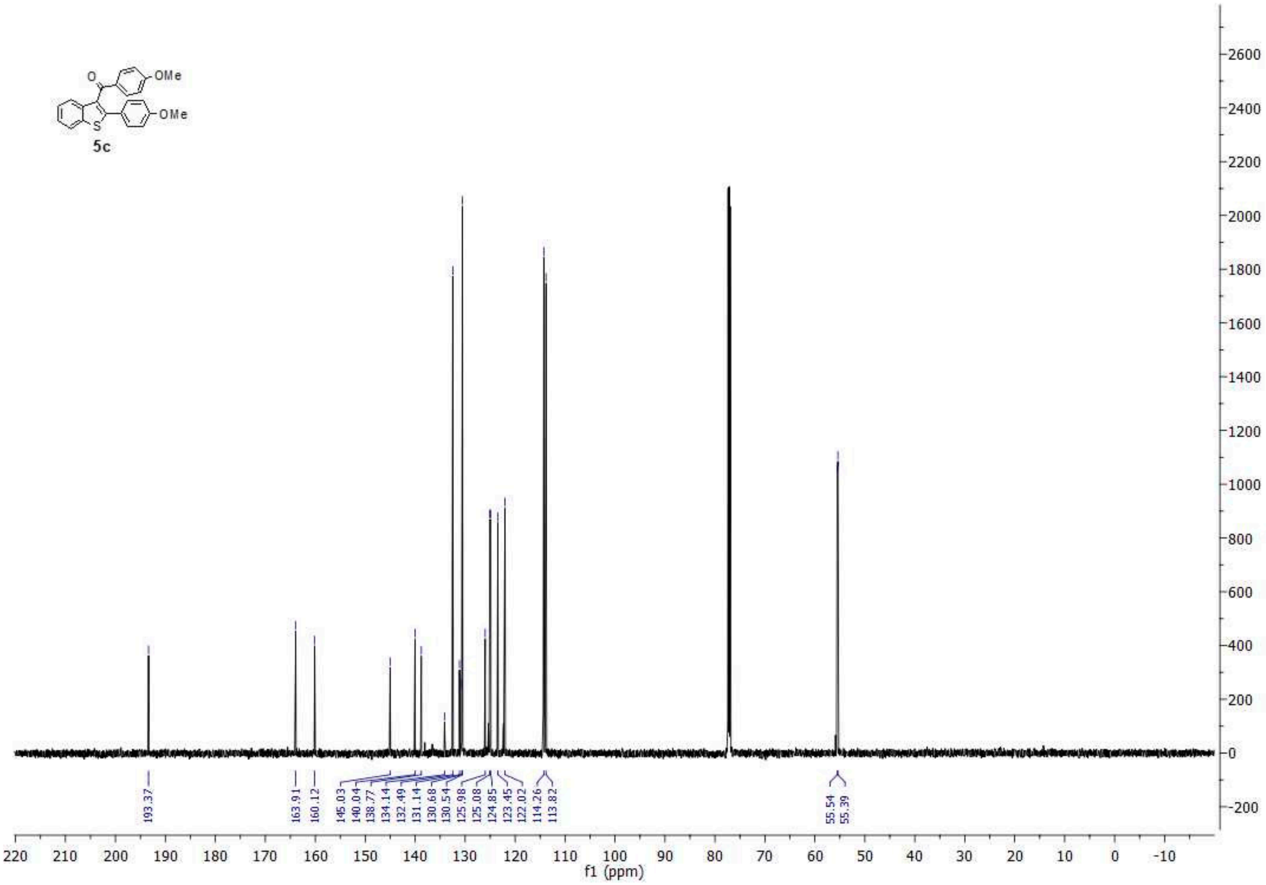

Figure S13.^{1}H NMR spectra of **4-benzothiophene-2-yl)benzonitrile (4d)**

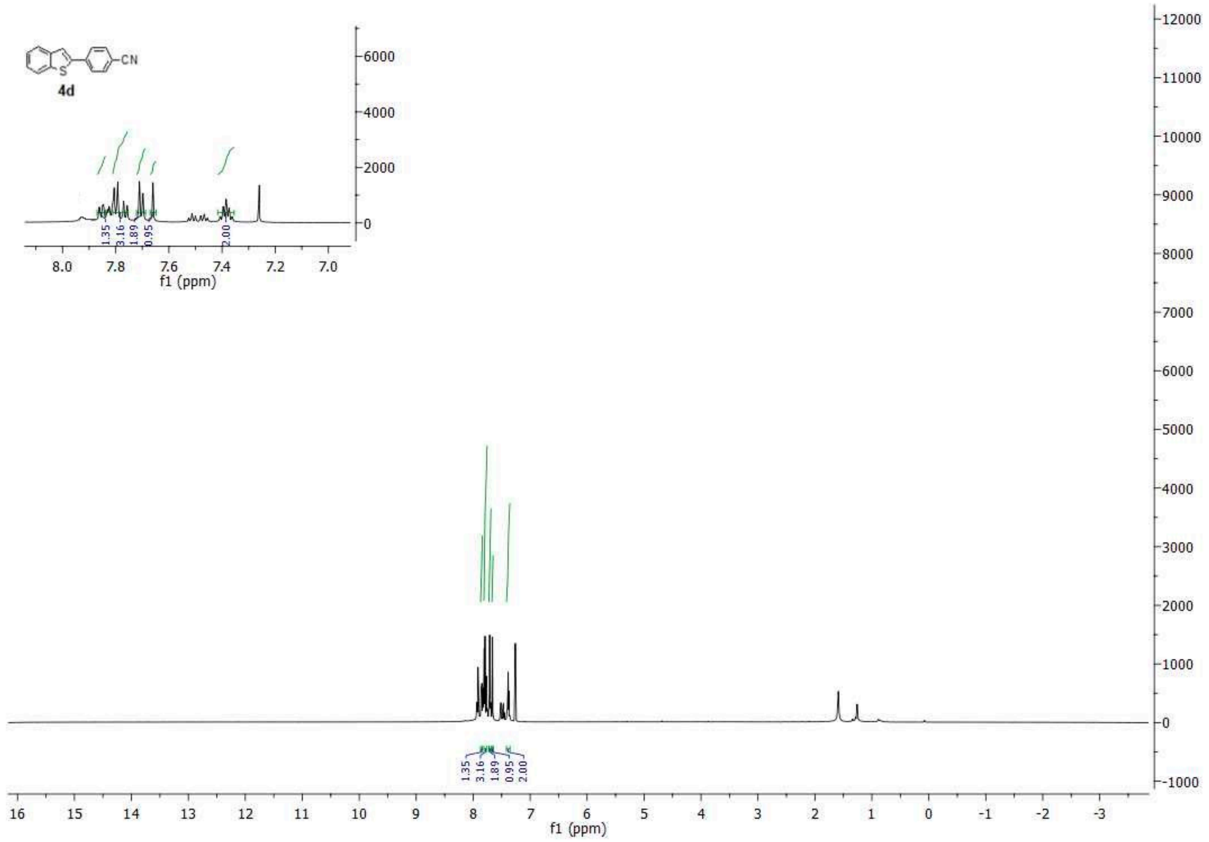

Figure S14.^{13}C NMR spectra of **4-(benzothiophene-2-yl)benzonitrile (4d)**

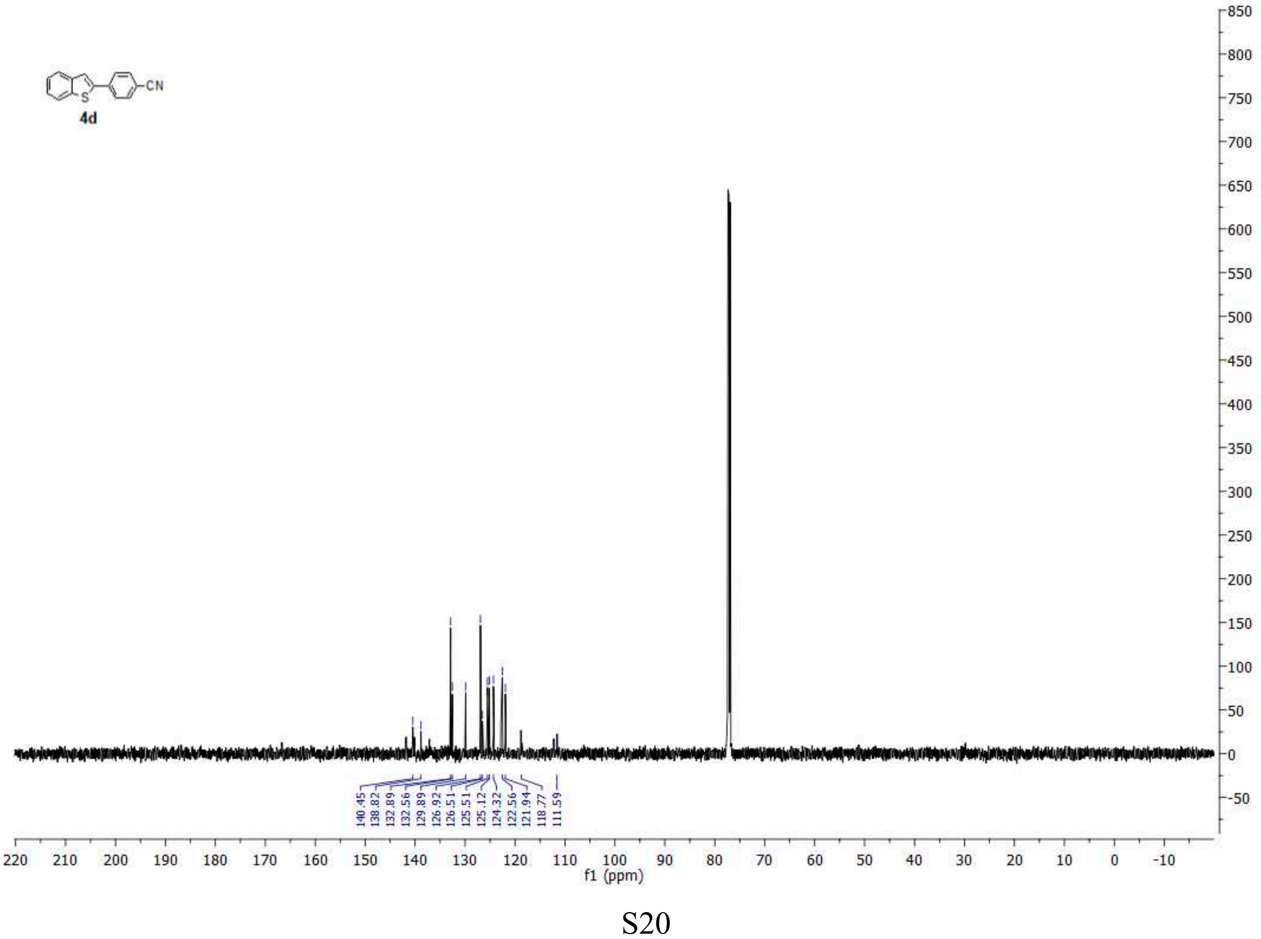

Figure S15.^{1}H NMR spectra of **4-cianophenyl-[2-(4-cianophenyl)-benzothiophen-3-yl)]-methanone (5d)**

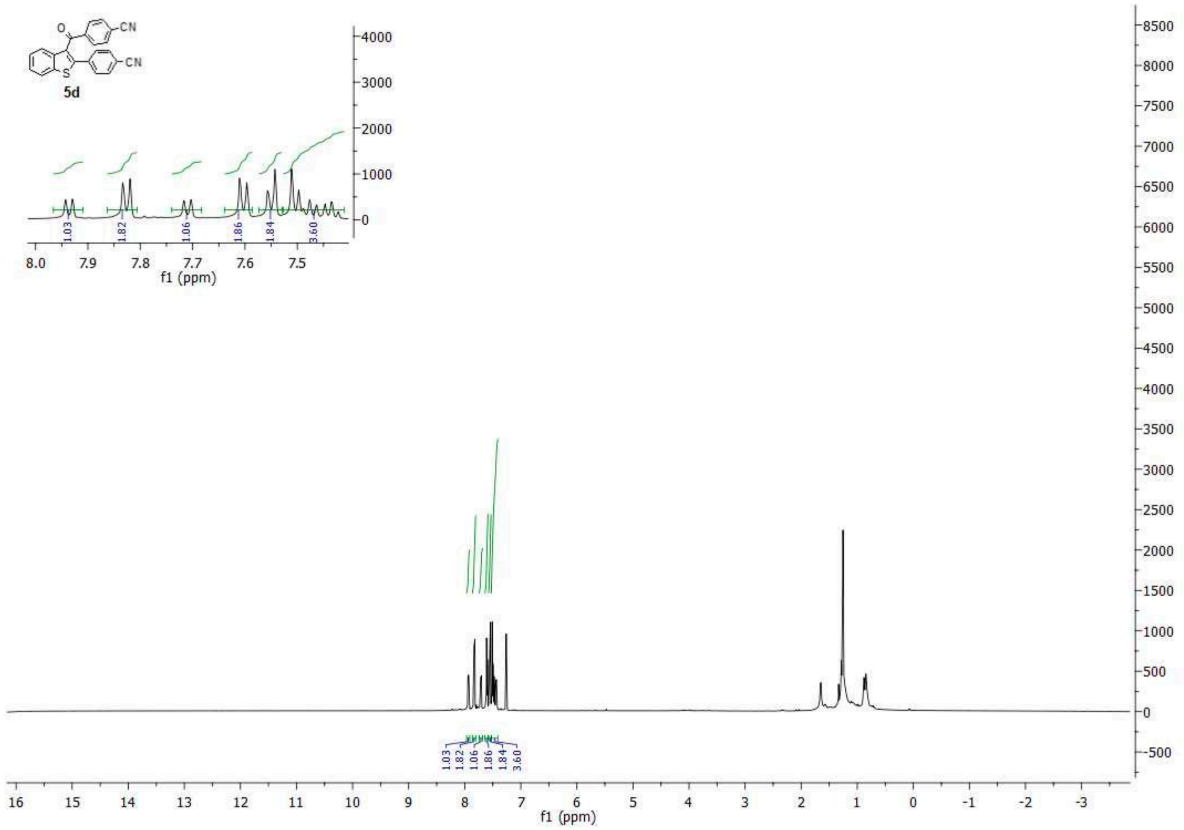

Figure S16.^{13}C NMR spectra of **4-cianophenyl-[2-(4-cianophenyl)-benzothiophen-3-yl)]-methanone (5d)**

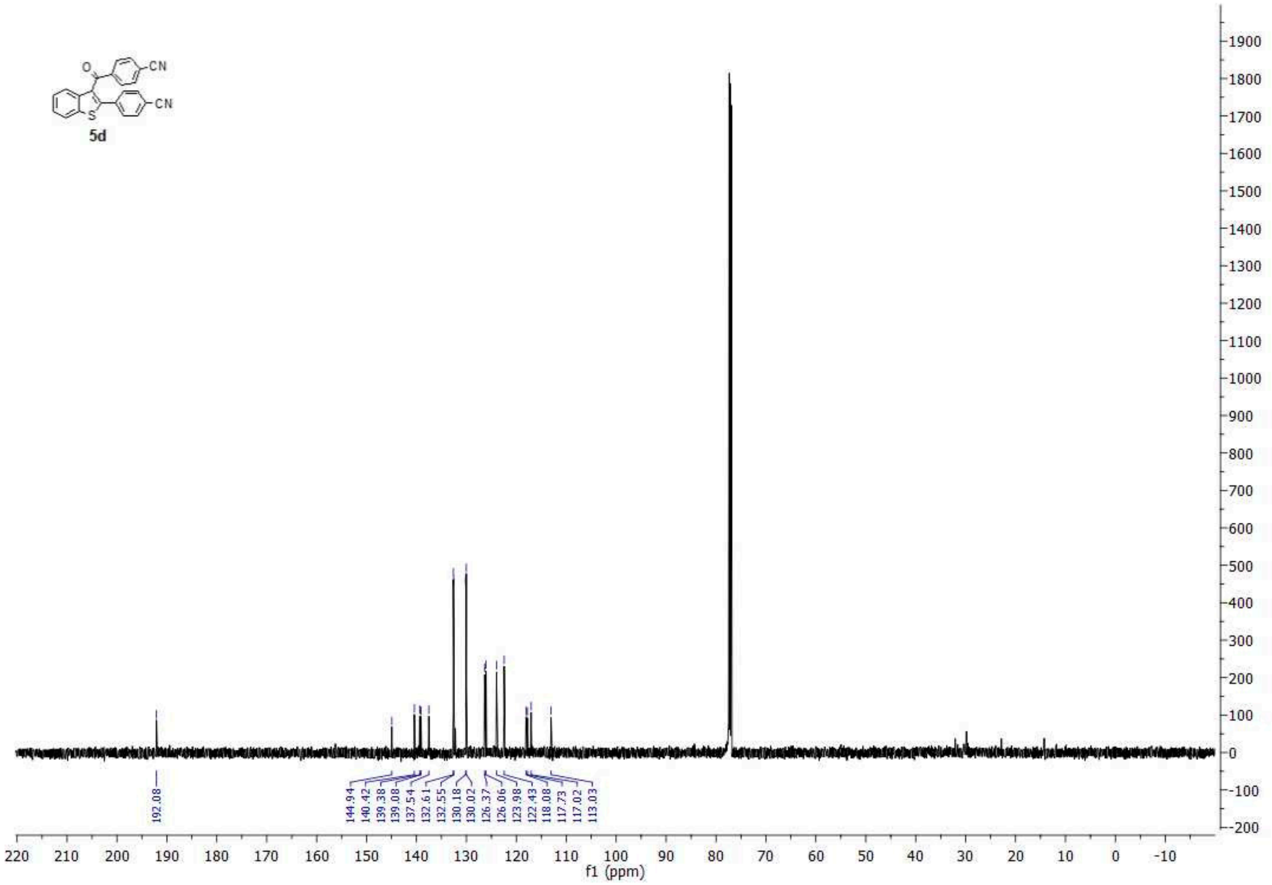

Figure S17.^{1}H NMR spectra of **2-(4-trifluoromethylfenil)benzotiophene (4e)**

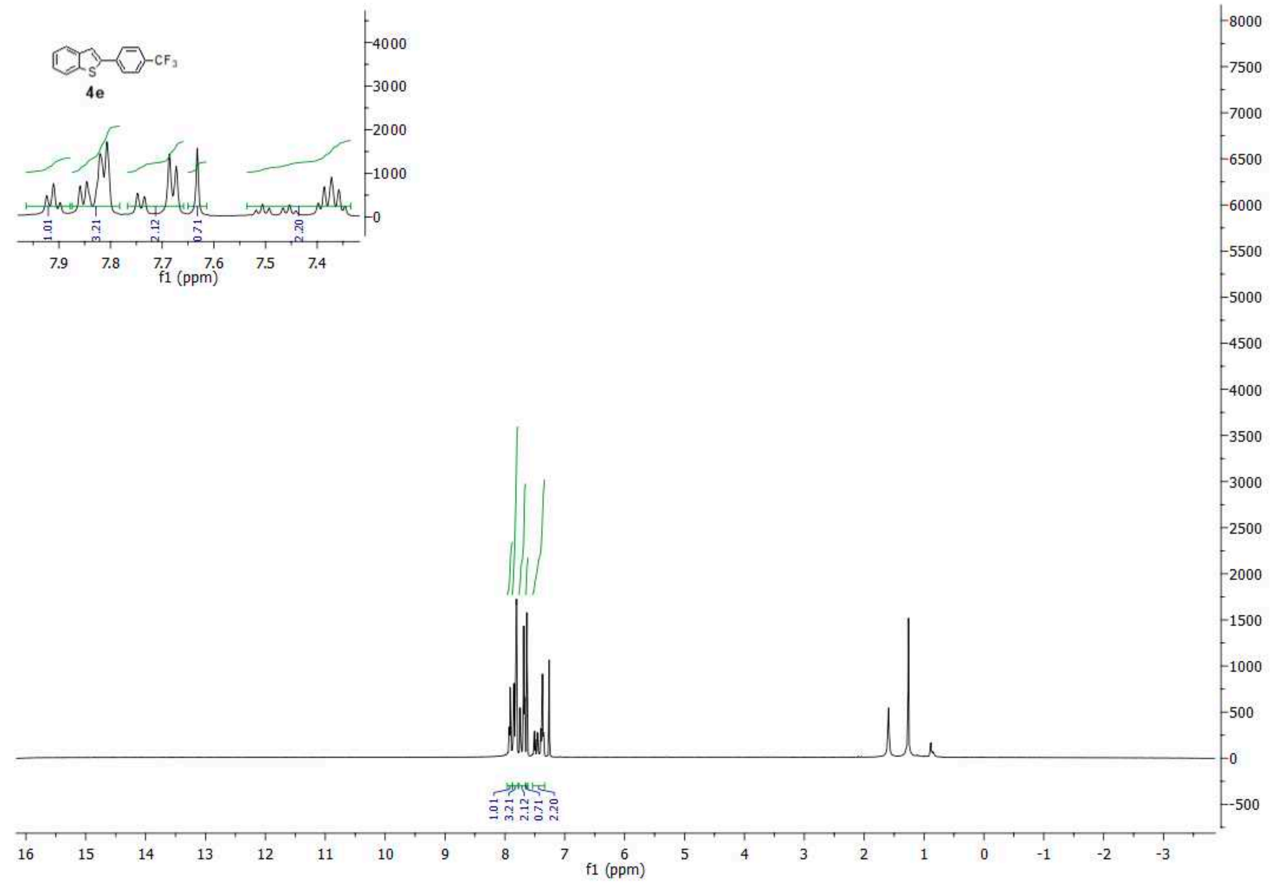

Figure S18.^{13}C NMR spectra of **2-(4-trifluoromethylfenil)benzotiophene (4e)**

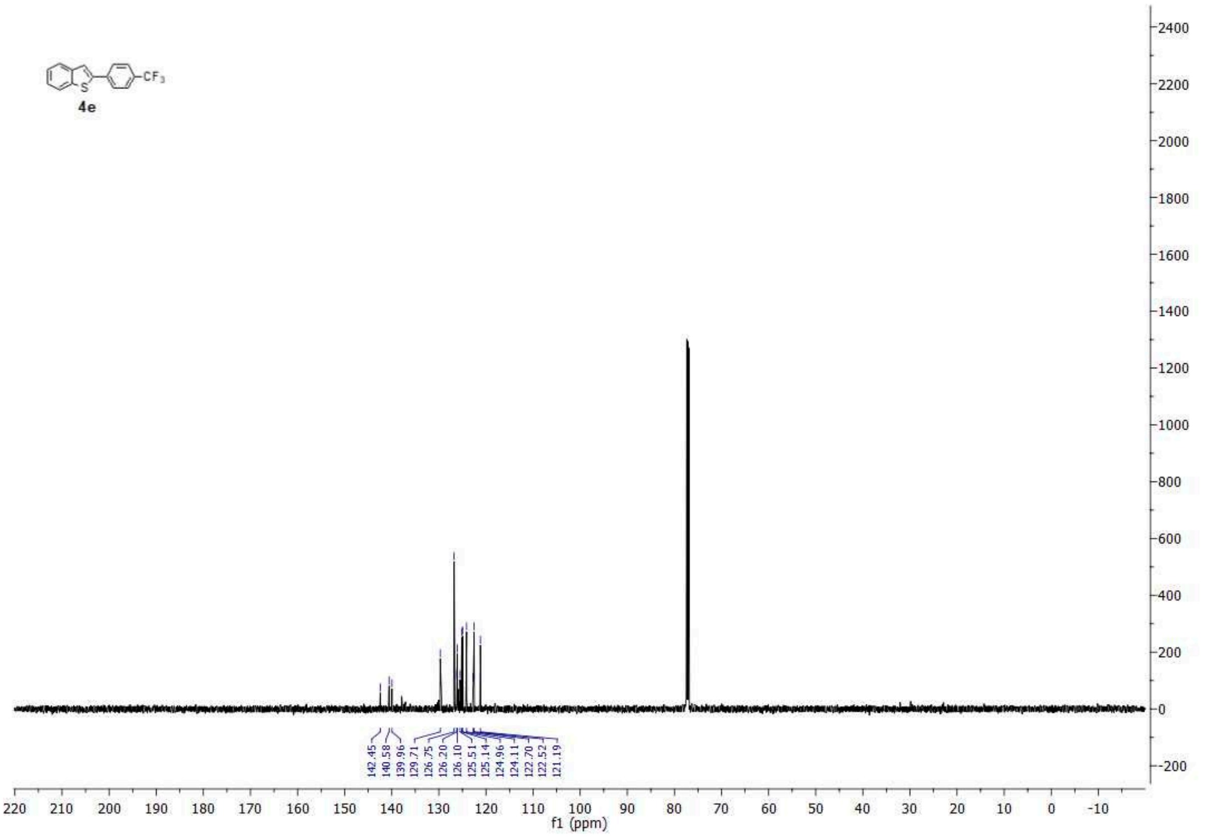

Figure S19.^{19}F NMR spectra of **2-(4-trifluoromethylfenil)benzotiophene (4e)**

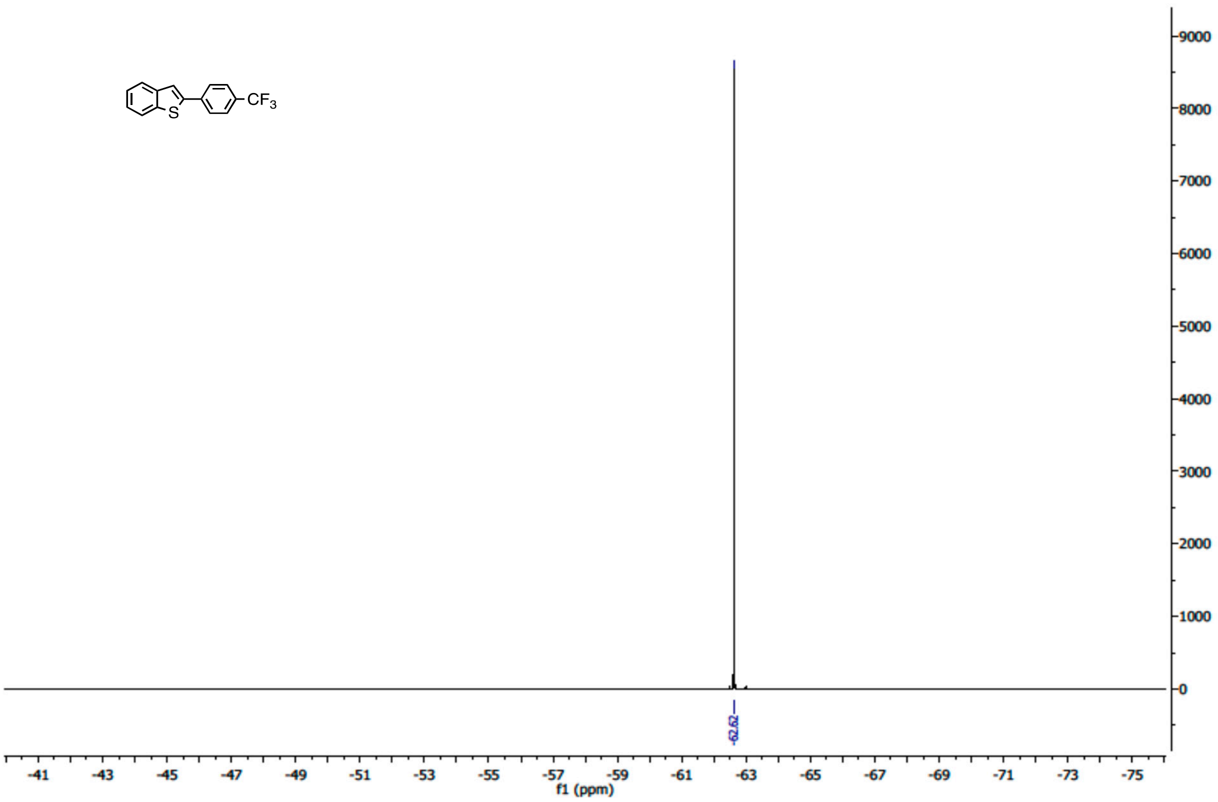

Figure S20.^{1}H NMR spectra of **3-(4-trifluorometilbenzoyl)-2-(4-trifluorometilfenil)benzotiofene (5e)**

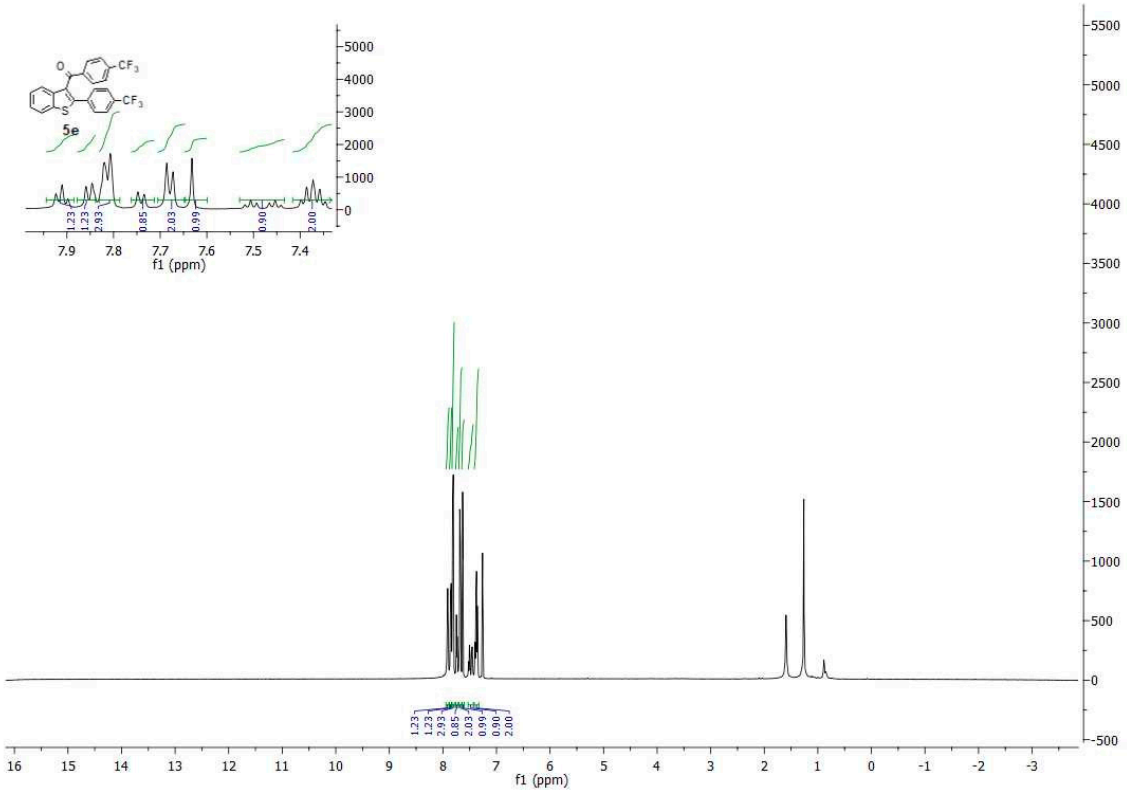

Figure S21.^{13}C NMR spectra of **3-(4-trifluorometilbenzoyl)-2-(4-trifluorometilfenil)benzotiofene (5e)**

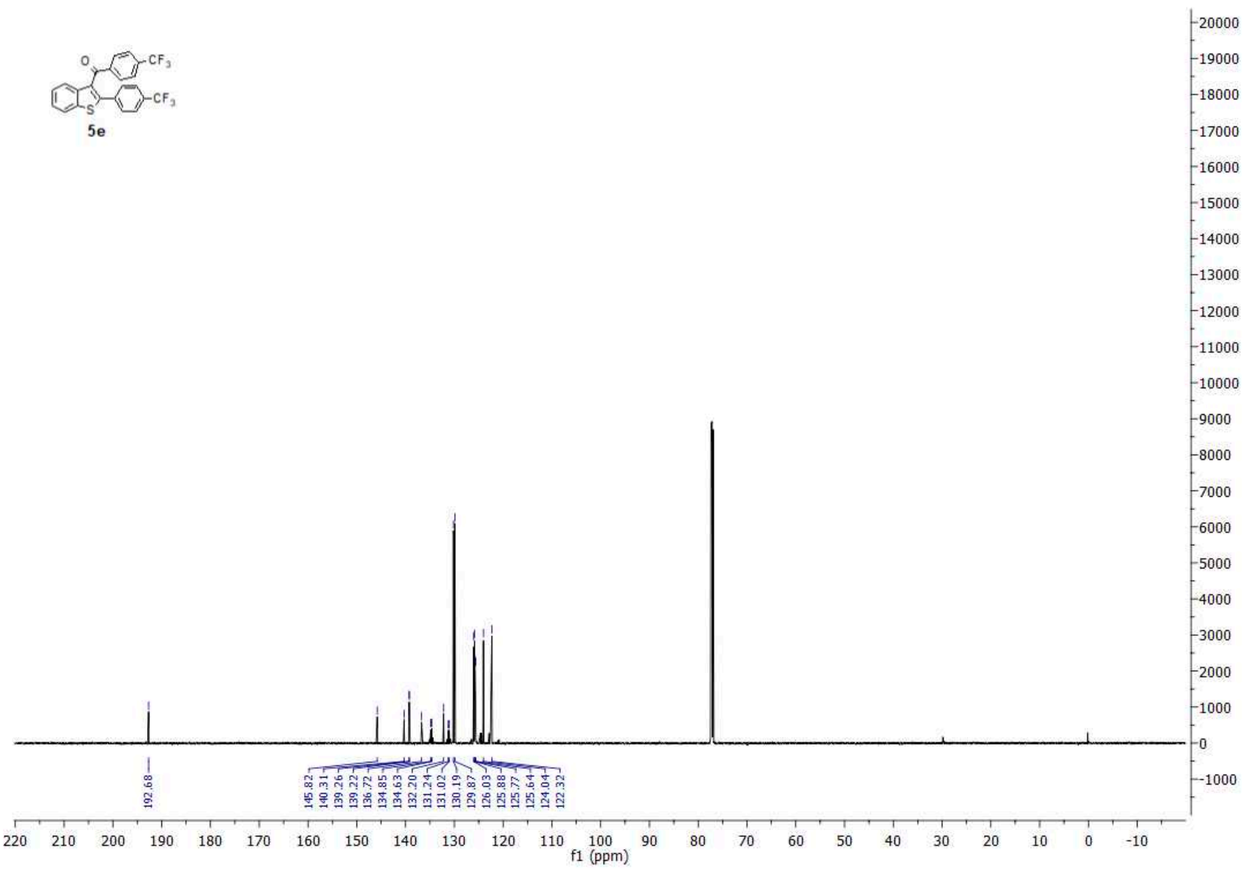

Figure S22.^{1}H NMR spectra of **3-(4-trifluorometilbenzoyl)-2-(4-trifluorometilfenil)benzotiofene (5e)**

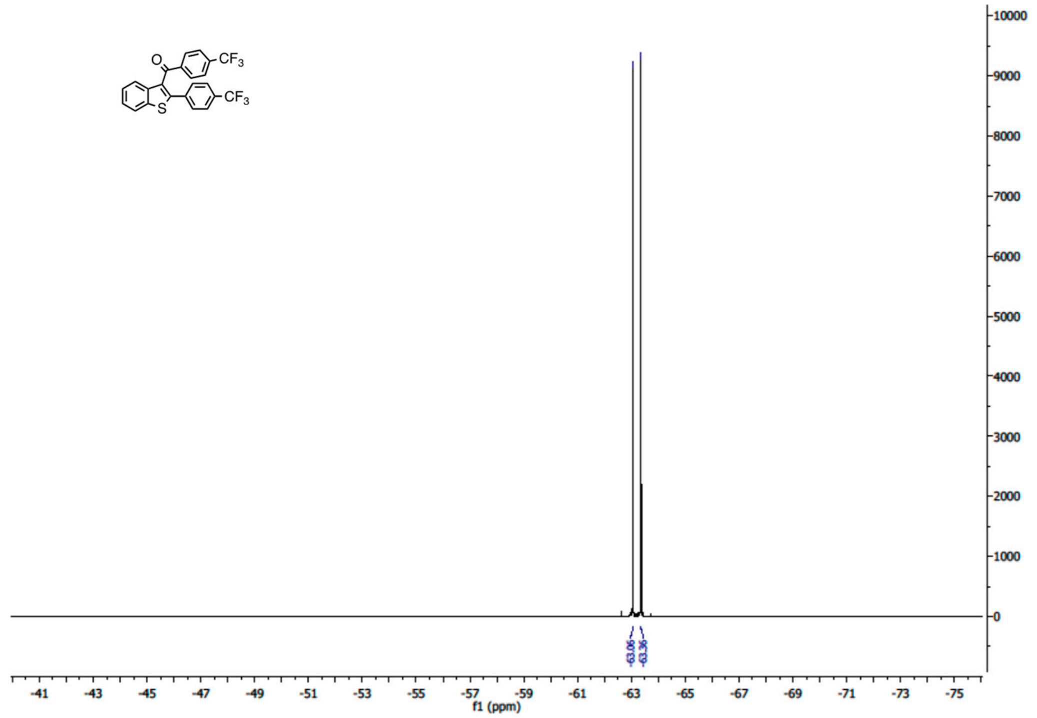

Figure S23.^{1}H NMR spectra of **2-(4-chlorophenyl)benzothiophene (4f)**

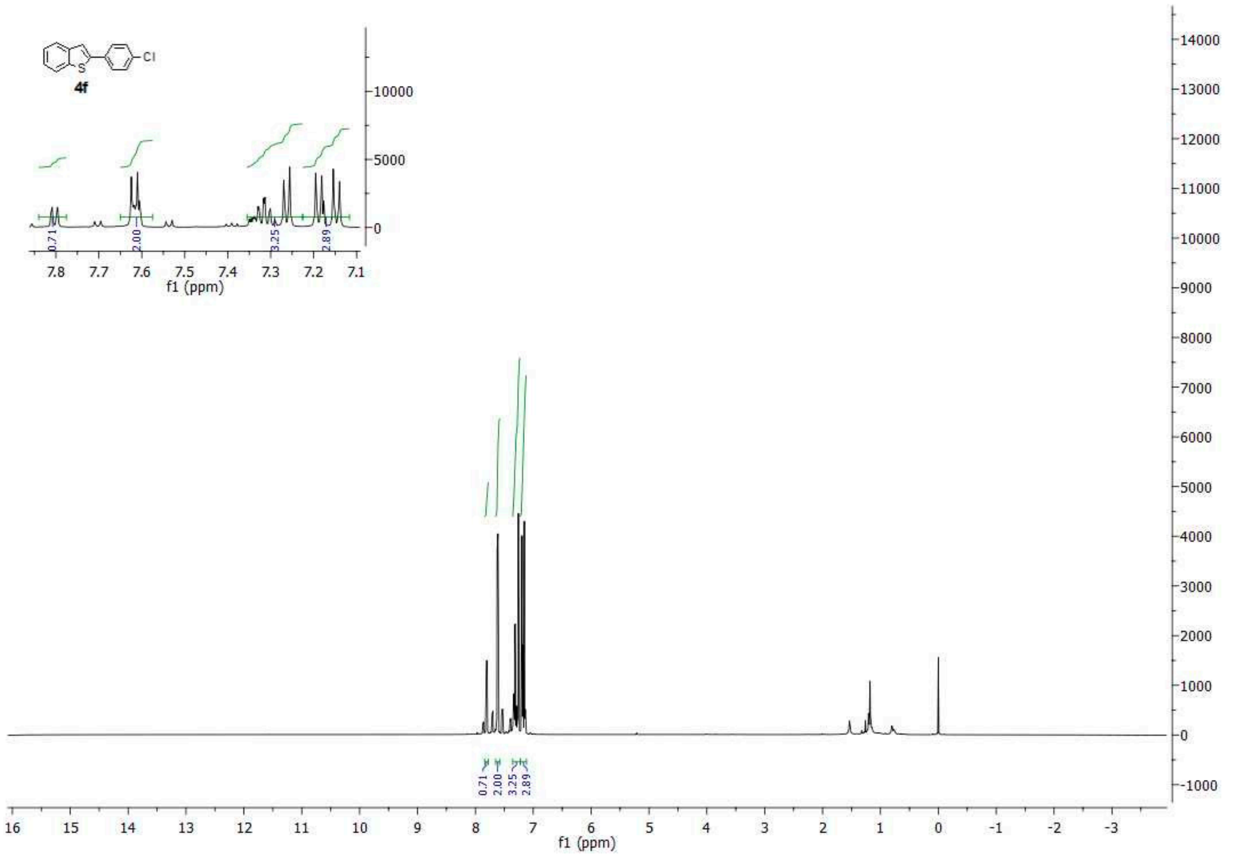

Figure S24.^{13}C NMR spectra of **2-(4-chlorophenyl)benzothiophene (4f)**

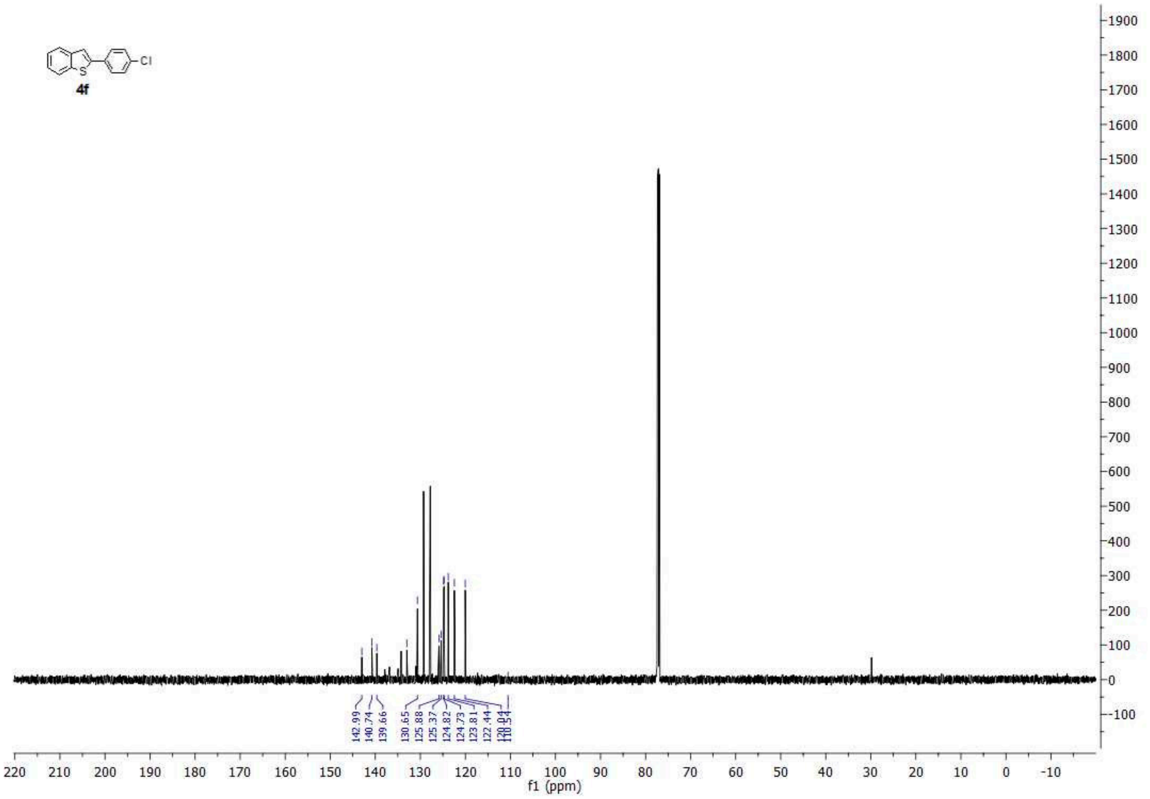

Figure S25.^{1}H NMR spectra of **3-(4-chlorobenzoyl)-2-(4-chlorophenyl)benzothiophene (5f)**

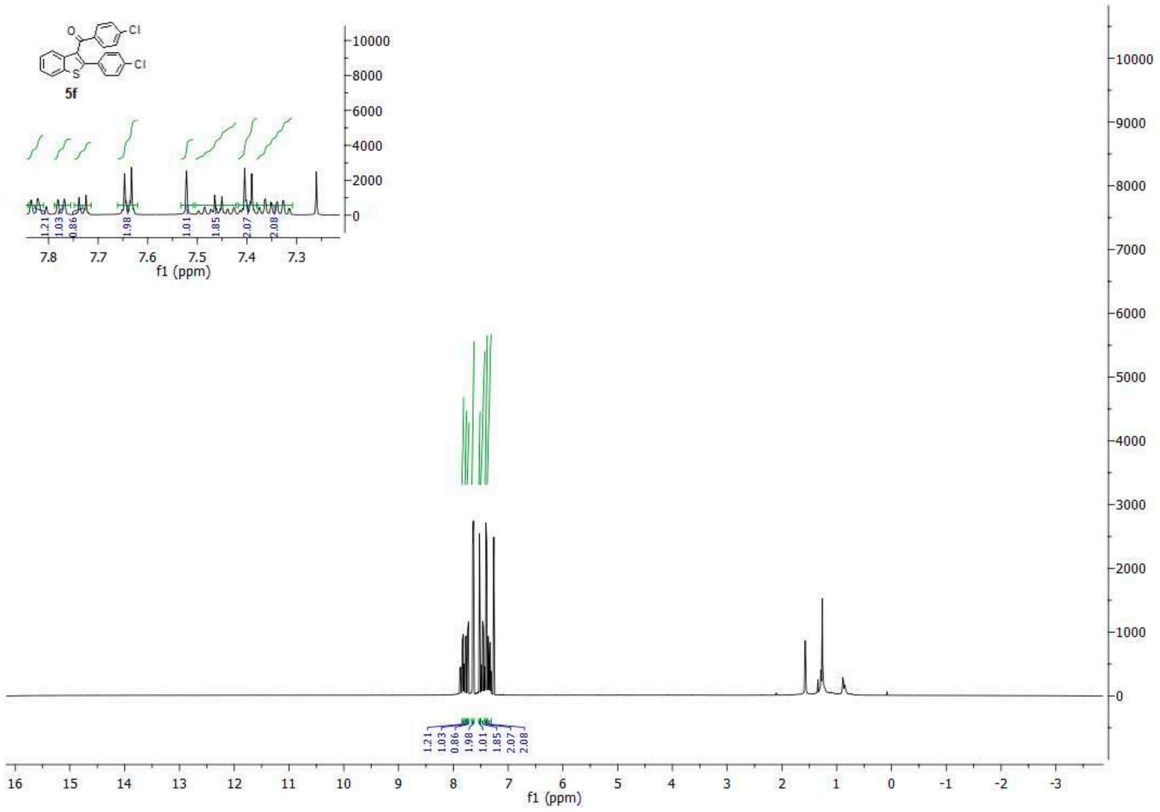

Figure S26.^{13}C NMR spectra of **3-(4-chlorobenzoyl)-2-(4-chlorophenyl)benzothiophene (5f)**

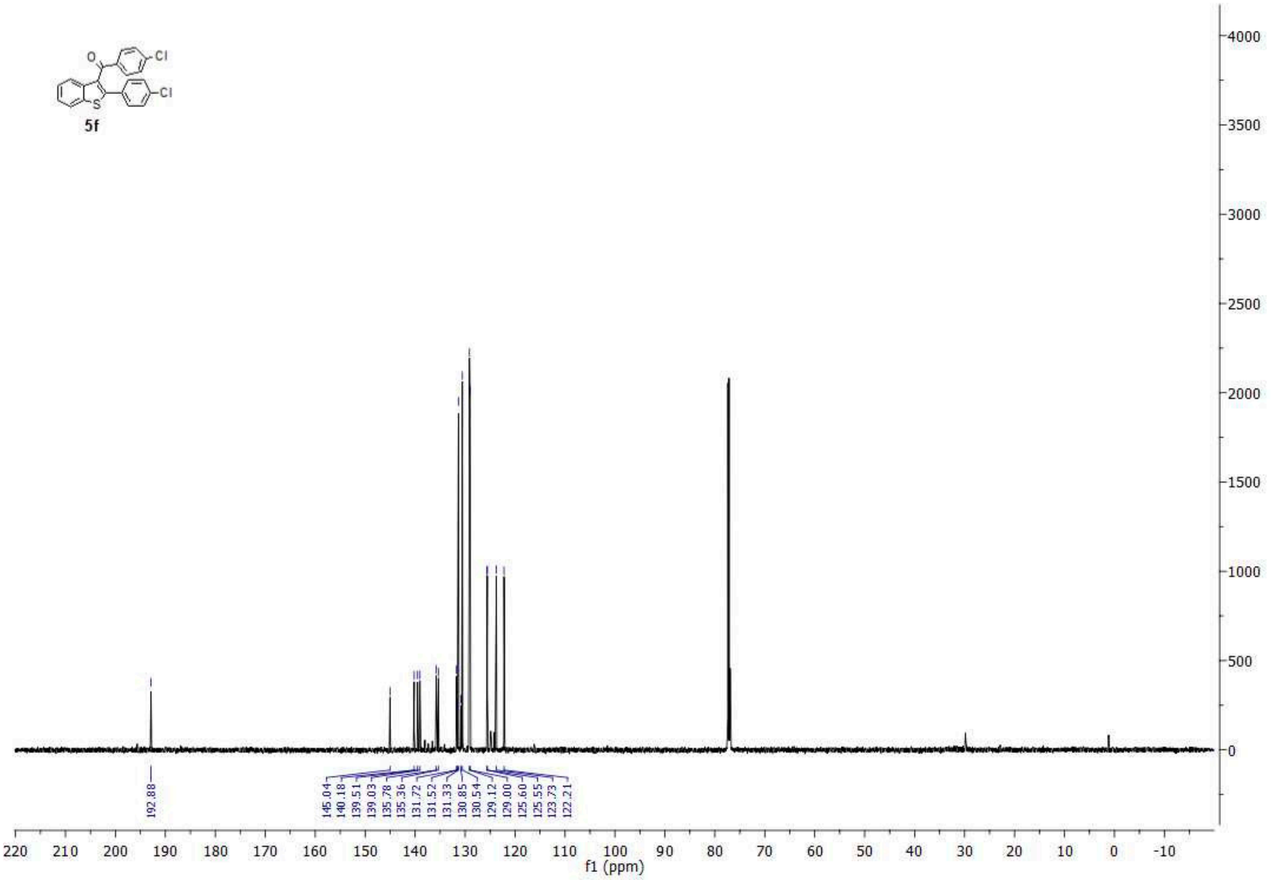

Figure S27.^{1}H NMR spectra of **2-(4-methylphenyl)benzothiophene (4g)**

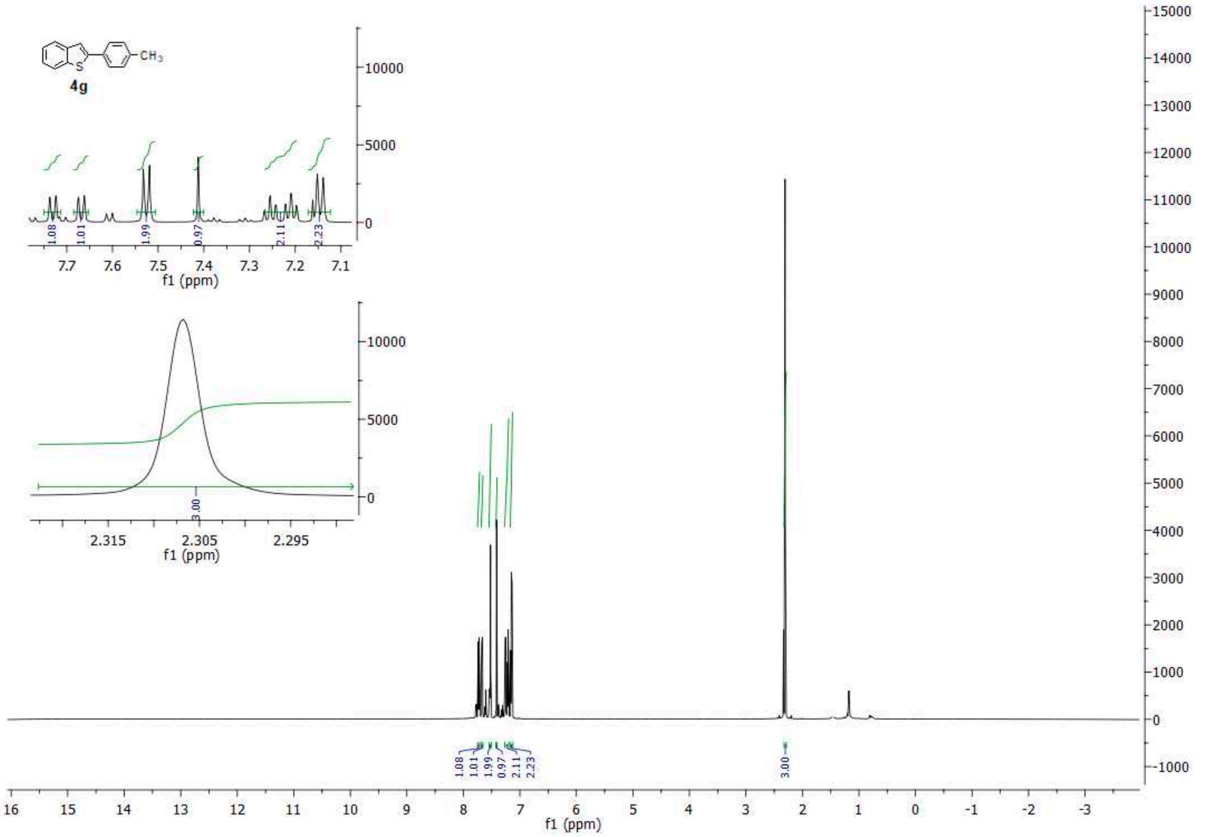

Figure S28.^{13}C NMR spectra of **2-(4-methylphenyl)benzothiophene (4g)**

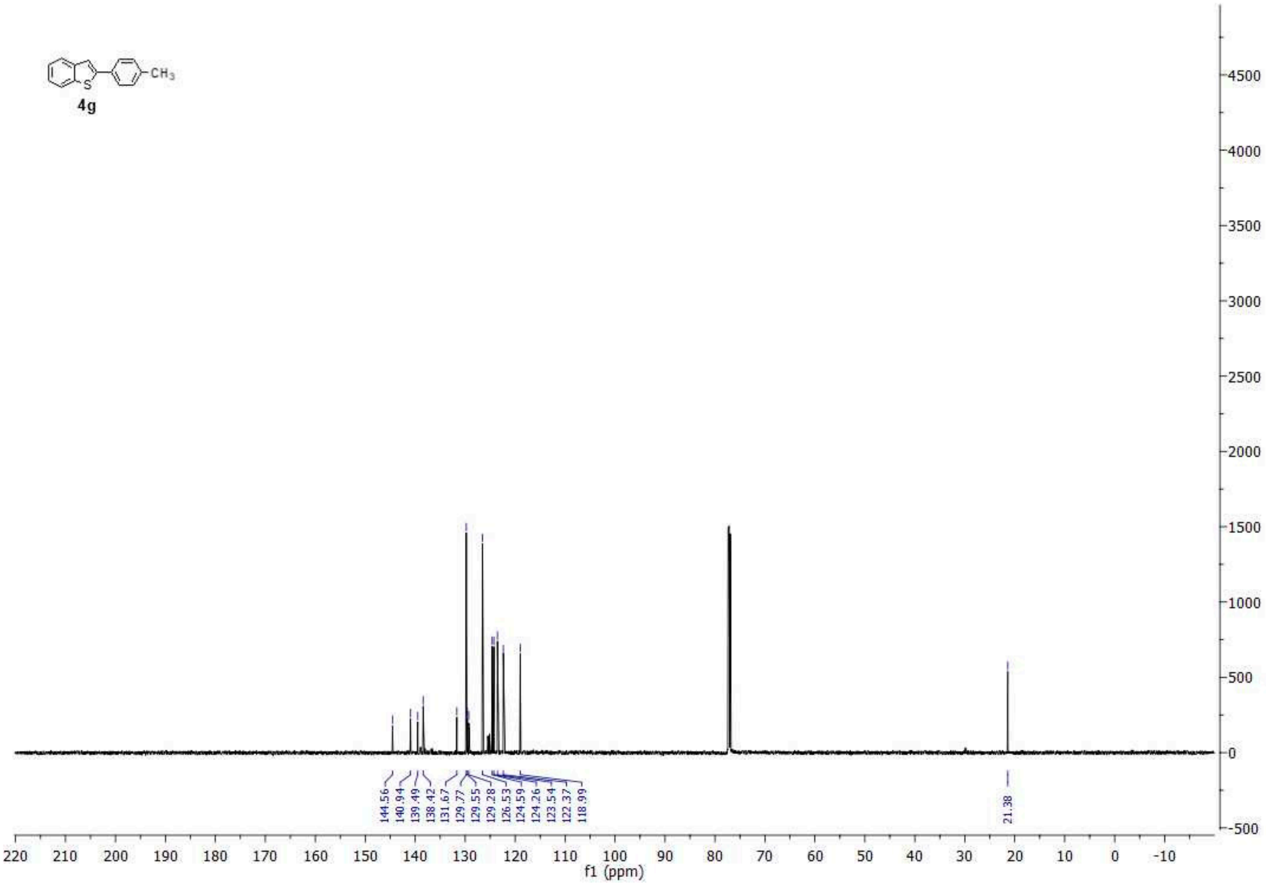

Figure S29.^{1}H NMR spectra of **3-(4-methylbenzoyl)-2-(4-methylphenyl)benzothiophene (5g)**

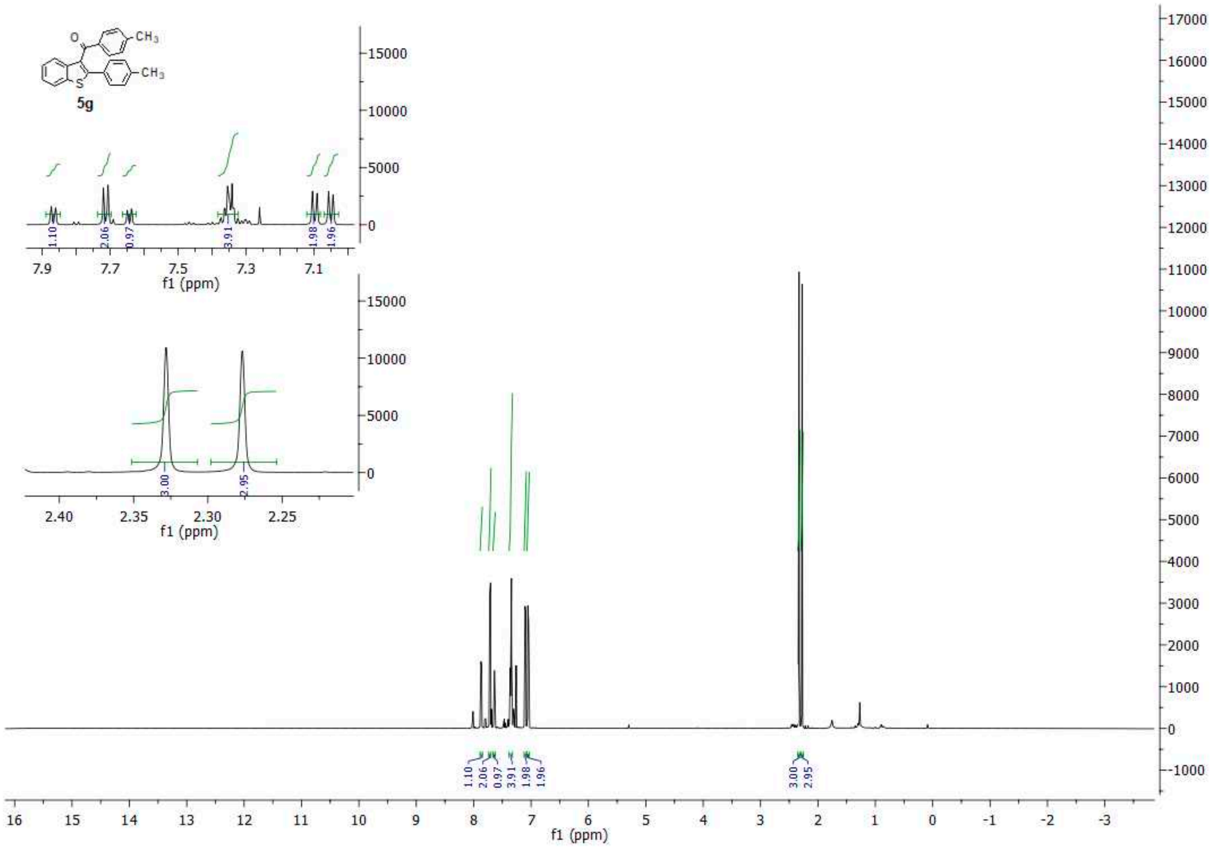

Figure S30.^{13}C NMR spectra of **3-(4-Methylbenzoyl)-2-(4-methylphenyl)benzothiophene (5g)**

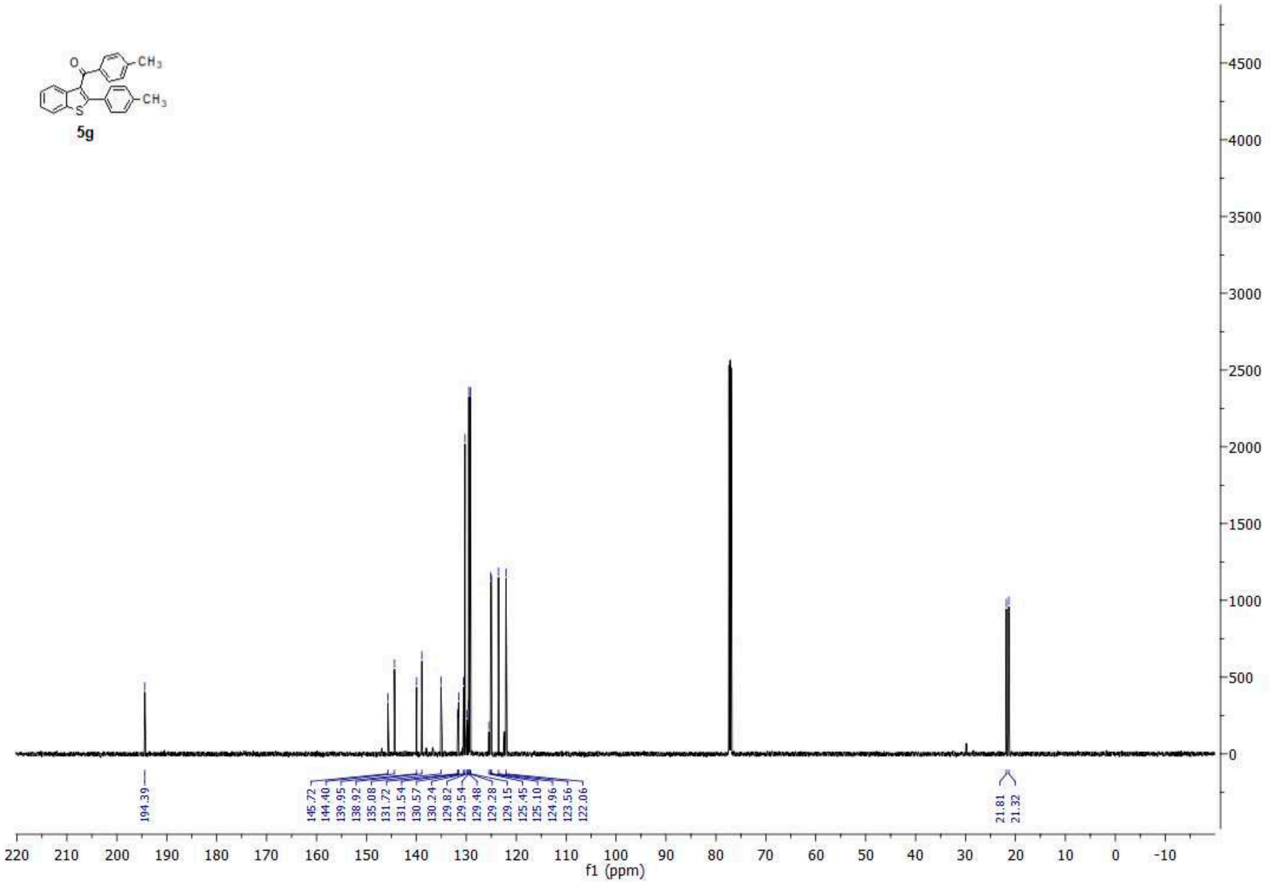

Figure S31.^{1}H NMR spectra of **2-(4-aminophenyl)benzothiophene (4h)**

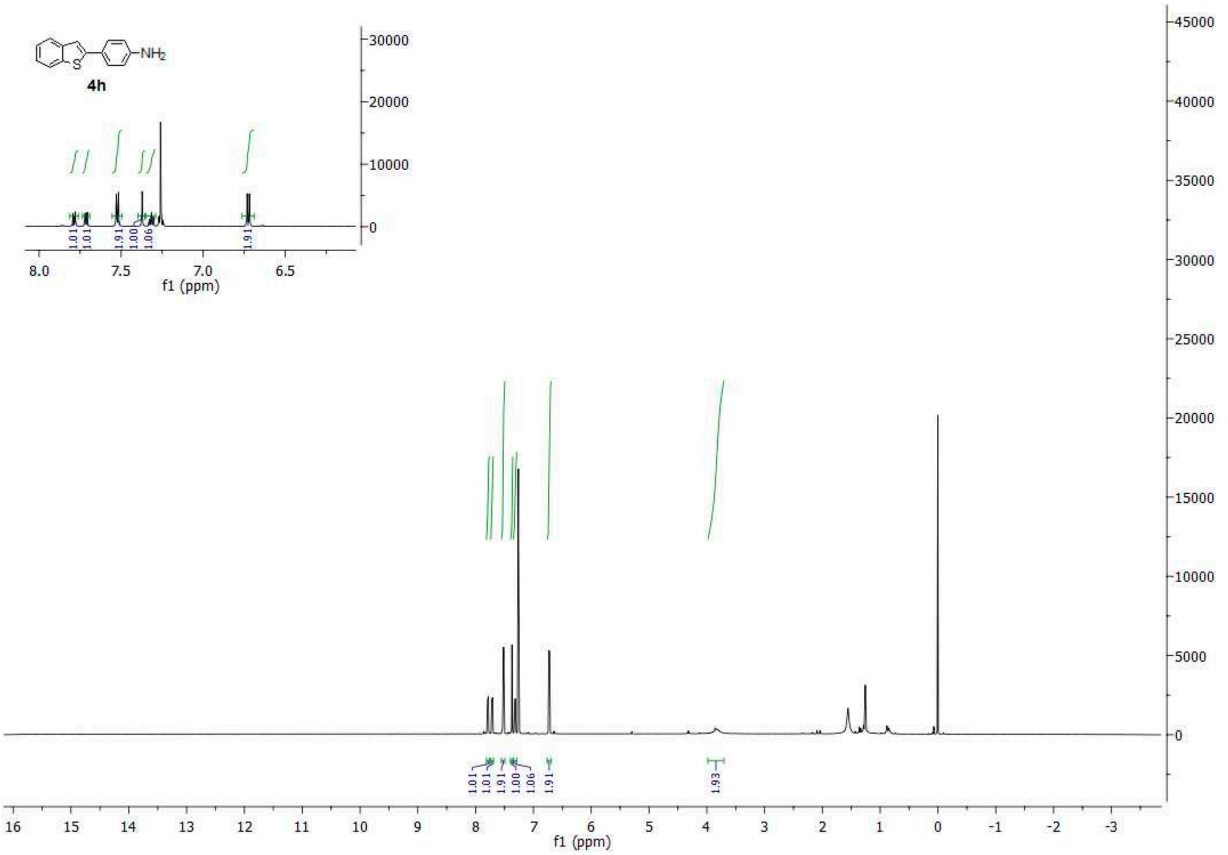

Figure S32.^{13}C NMR spectra of **2-(4-aminophenyl)benzothiophene (4h)**

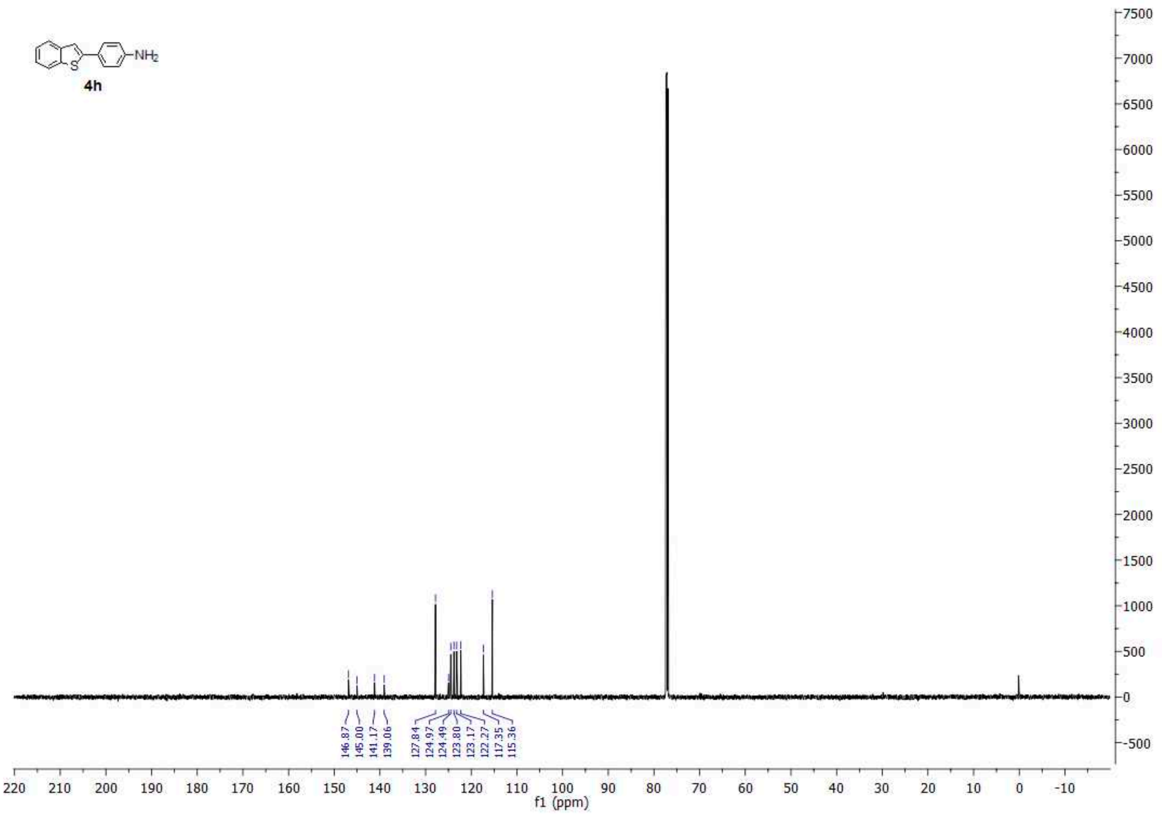

Figure S33.^{1}H NMR spectra of **3-(4-aminobenzoyl)-2-(4-aminophenyl)benzothiophene (5h)**

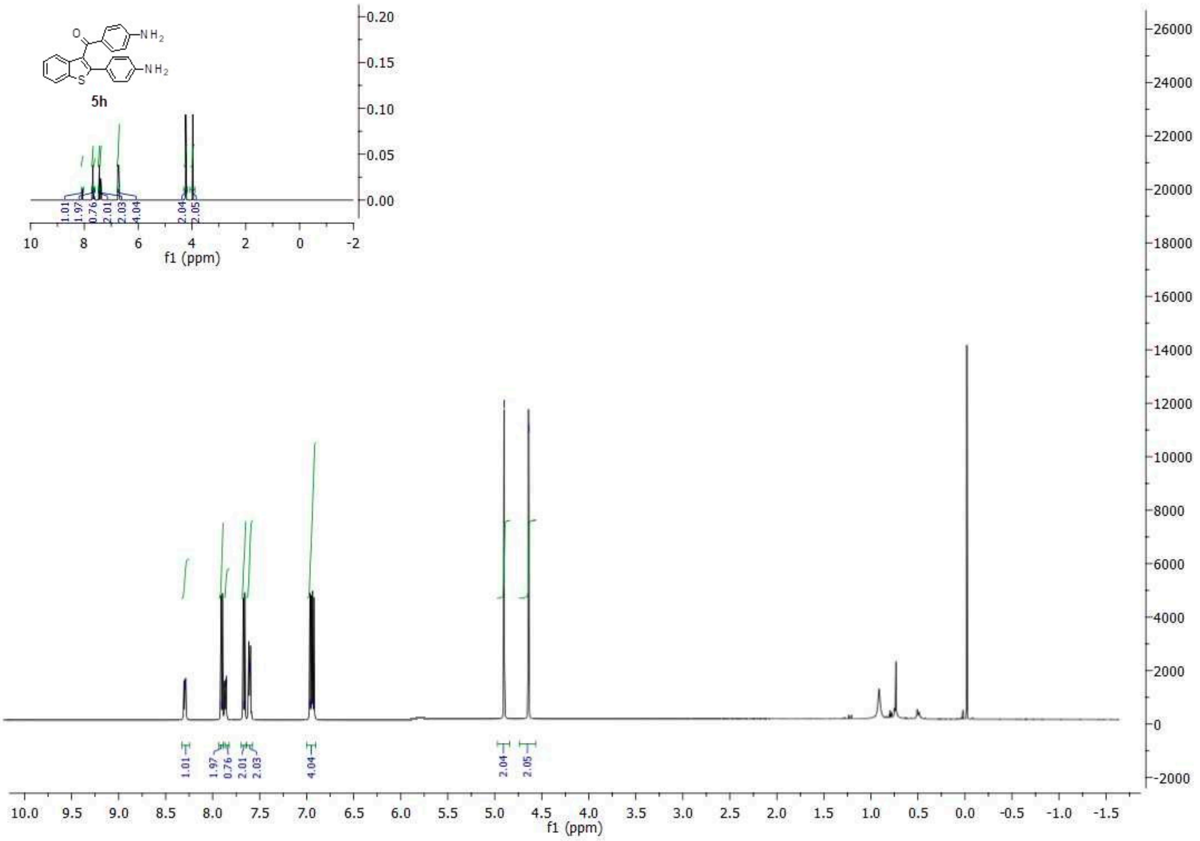

Figure S34.^{13}C NMR spectra of **3-(4-aminobenzoyl)-2-(4-aminophenyl)benzothiophene (5h)**

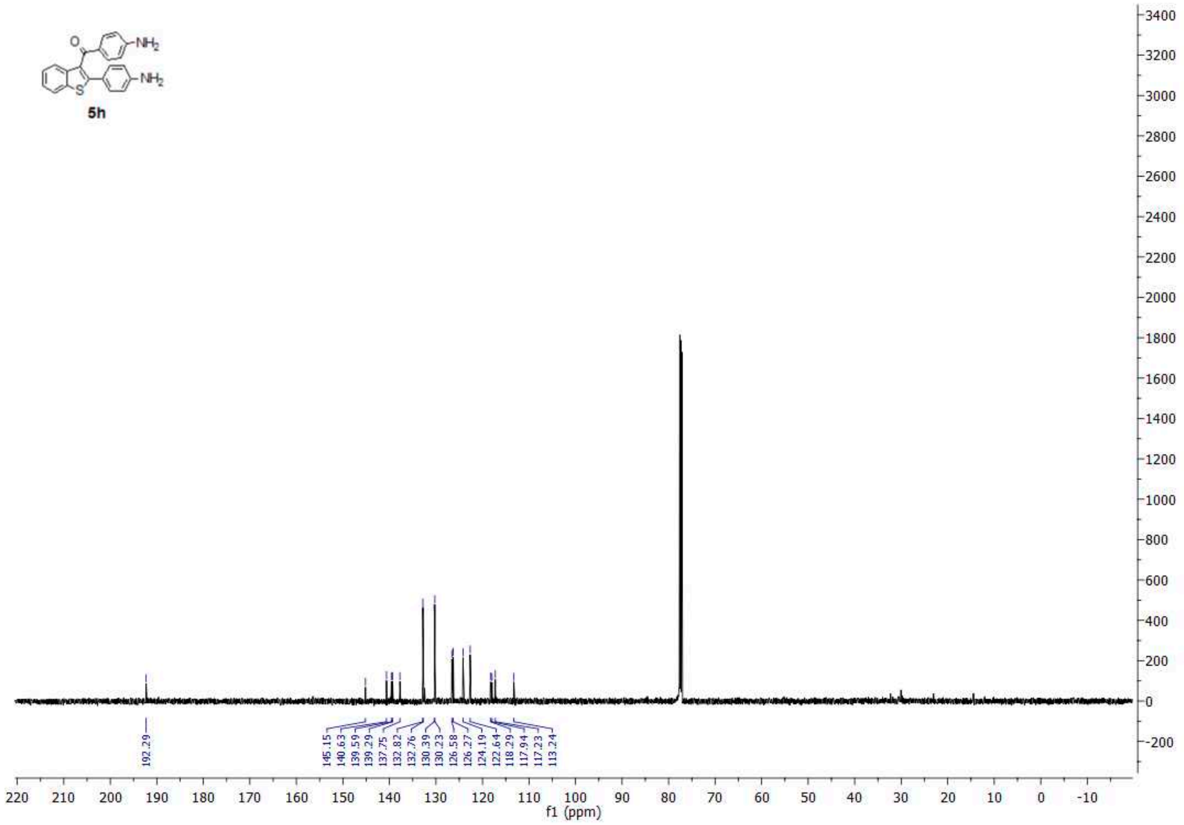

Figure S35.^{1}H NMR spectra of **2-(4-hydroxyphenyl)benzothiophene (4i)**

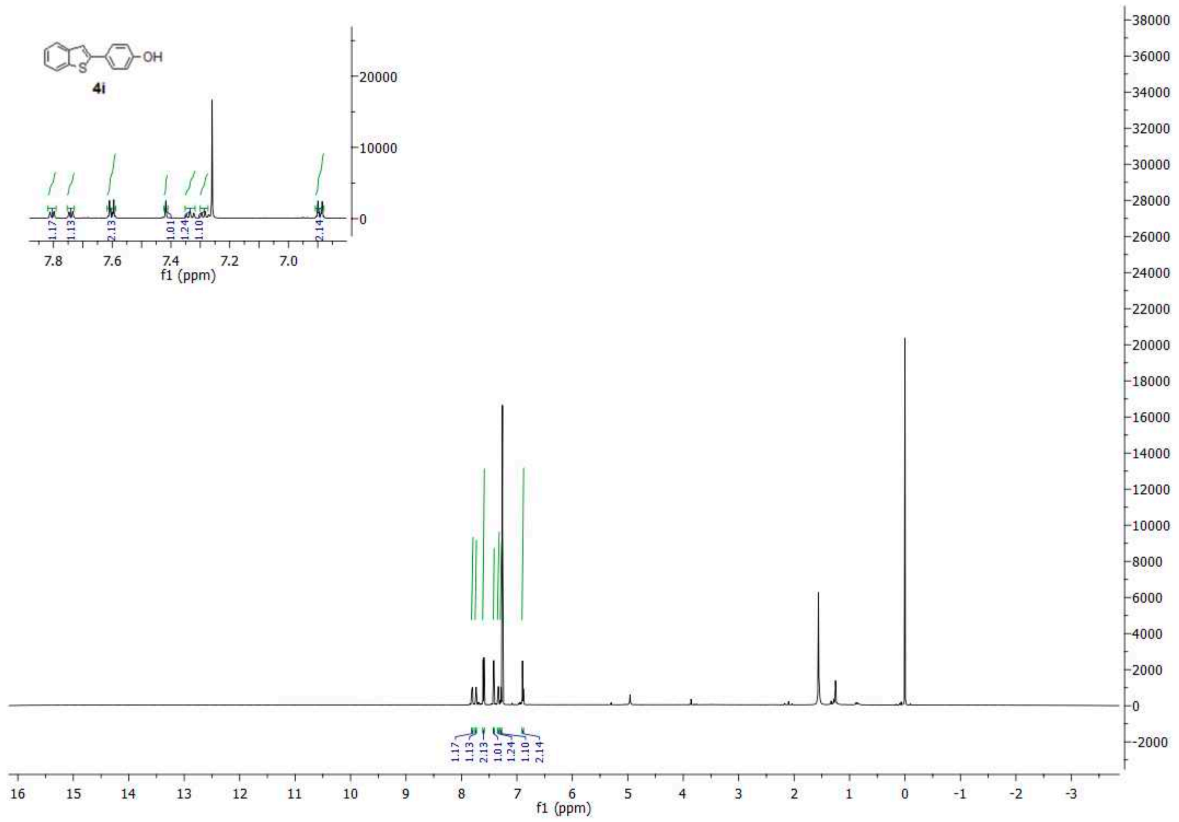

Figure S36.^{13}C NMR spectra of **2-(4-hydroxyphenyl)benzothiophene (4i)**

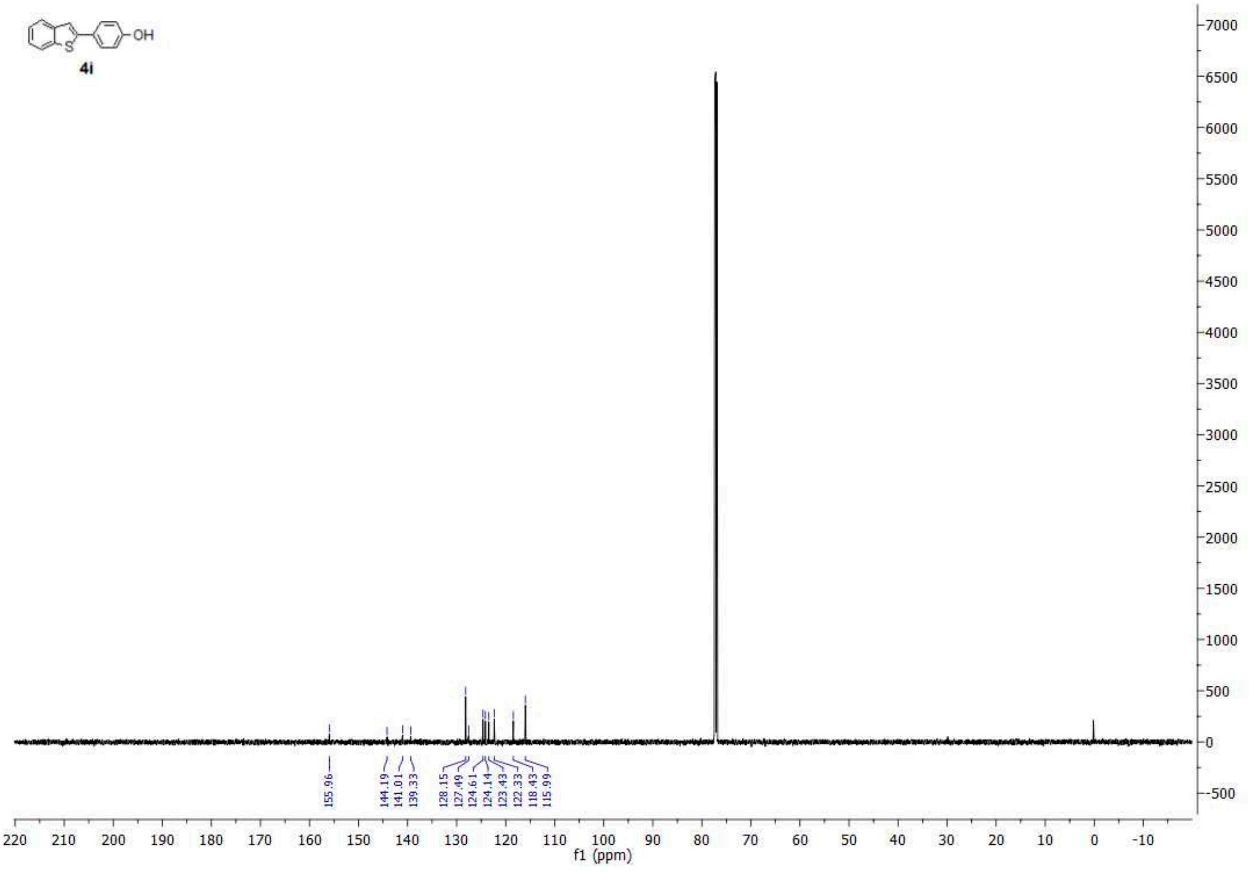

Figure S37.^{1}H NMR spectra of **3-(4-hydroxybenzoyl)-2-(4-hydroxyphenyl)benzothiophene (5i)**

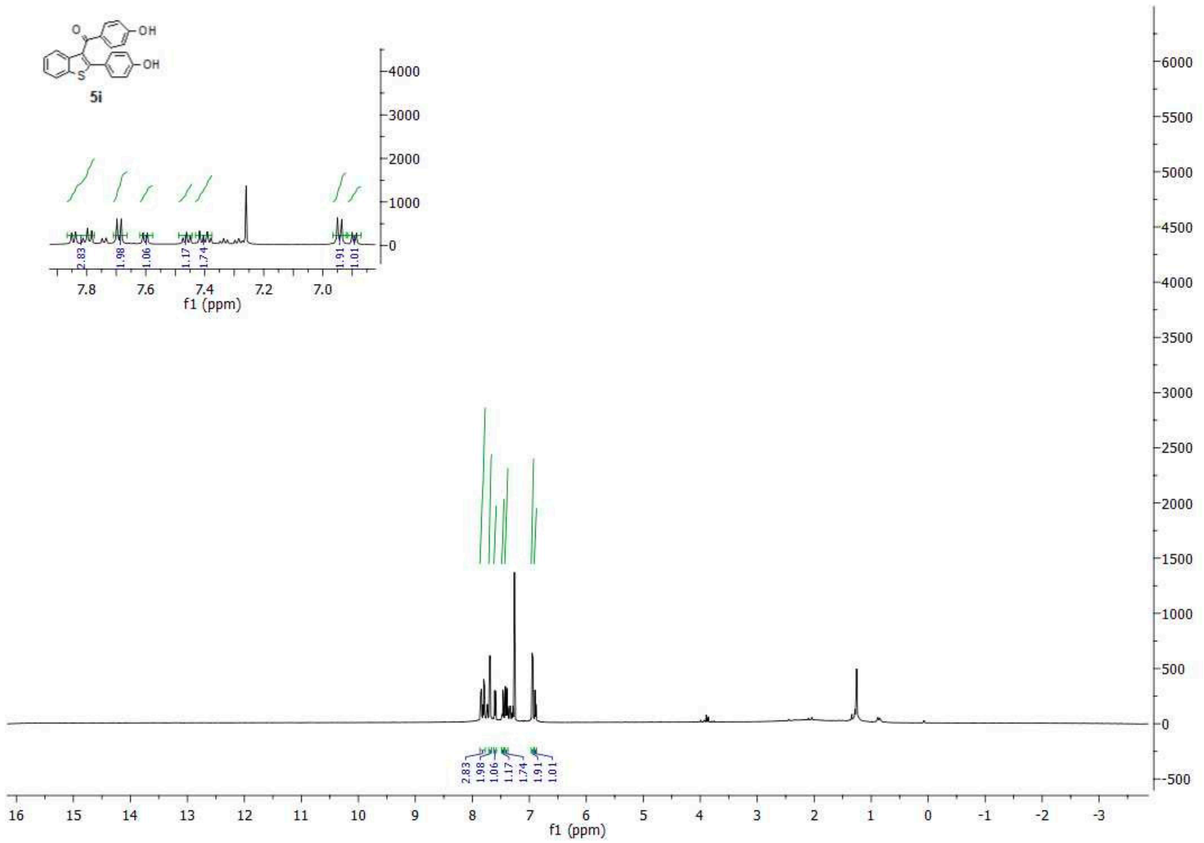

Figure S38.^{13}C NMR spectra of **3-(4-hydroxybenzoyl)-2-(4-hydroxyphenyl)benzothiophene (5i)**

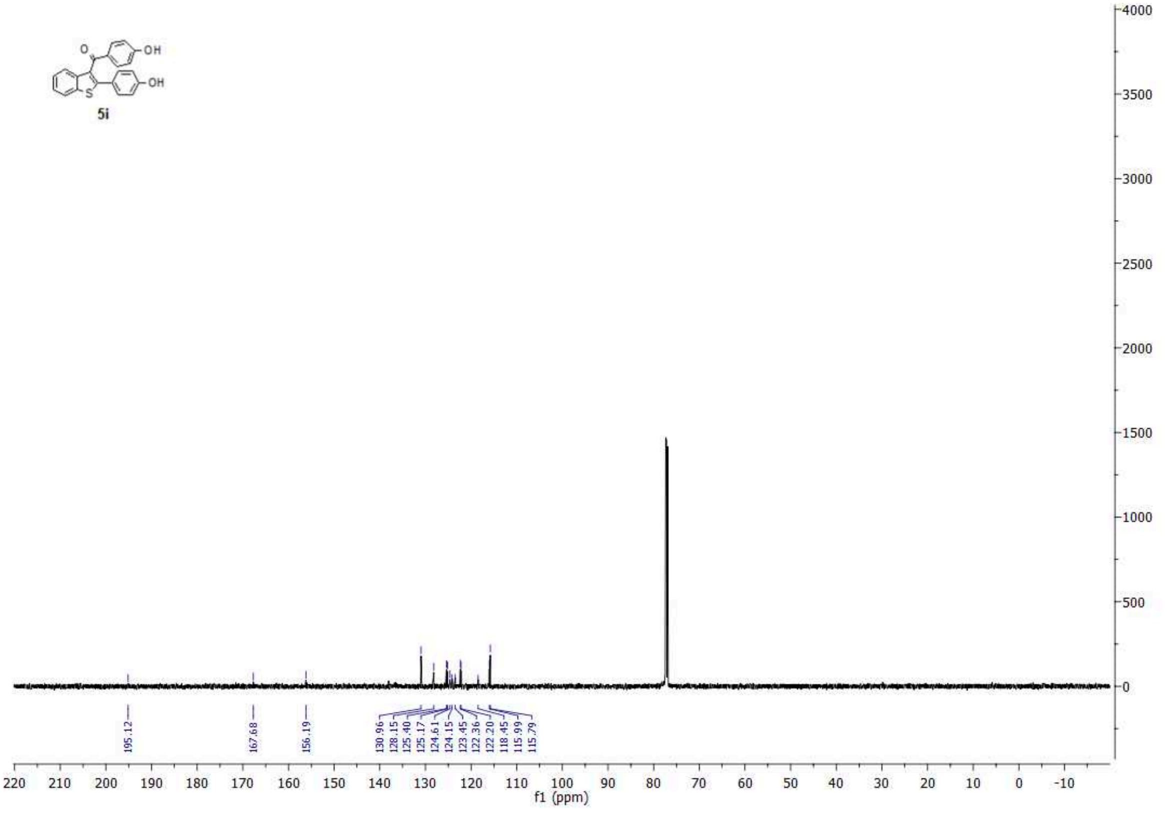

3. Biological Activity

3.1. Dose response curves

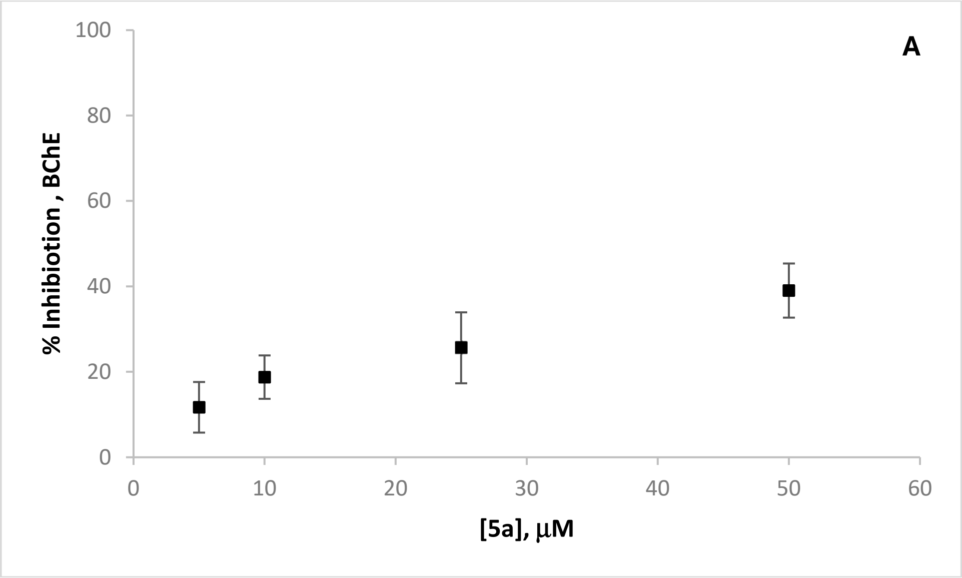

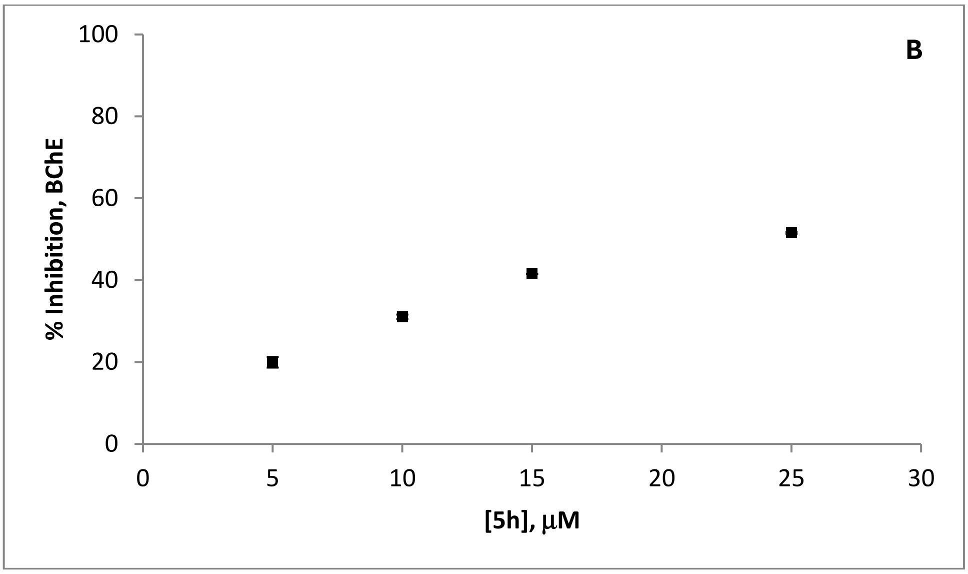

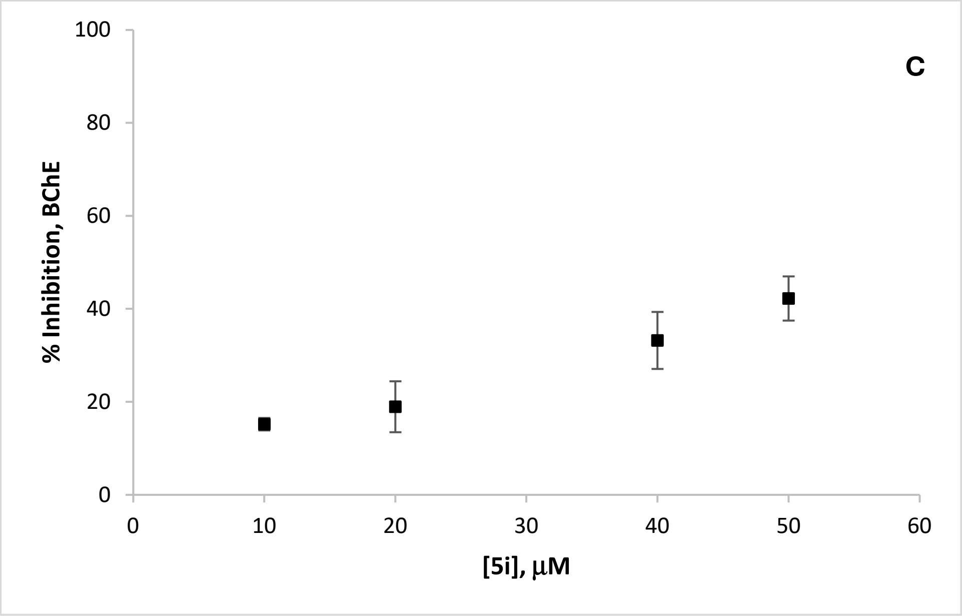

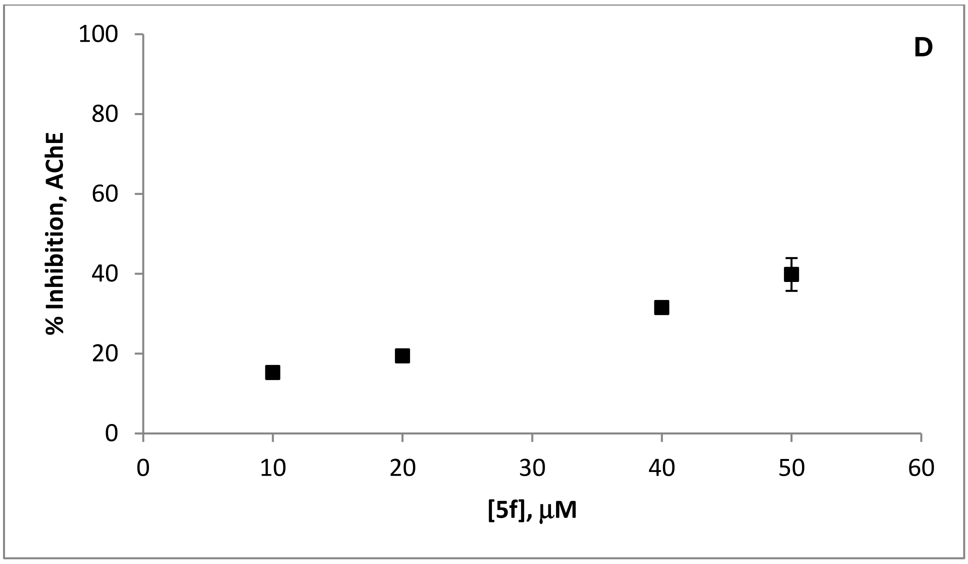

Figure S39. The dose-response curves to determine the IC_{50} values of the compounds **5a**, **5h**, **5i** against BChE (A, B and C) and **5f** against AChE (D).

4. References

1. Y.S. Chang, J.M. Jeong, Y.S. Lee, H.W. Kim, R.B. Ganesha, Y.J. Kim, D.S. Lee, J.K. Chung, M.C. Lee. Nuclear Medicine and Biology 33 (2006) 811-820.

2. A. Arnoldi, M. Carughi. Synthesis, 1988 (1988) 155-157.

3. T. Cai, C. Feng, F. Shen, K. Bian, C. Wu, R. Shen, Y. Gao. Eur. J. Org. Chem. (2021) 653-656.

4. C. Wang, L.L. Sun, B.L. Hu, X.G. Zhang, F. Chen. Tetrahedron. 70 (2014) 7969-7972.

5. K. Toyota, H. Tanaka, T. Hanagasaki. Results in Chemistry. 4 (2022) 1000487.

6. F. Gao, J.T. Wang, L.L. Liu, N. Ma, C. Yang, Y. Gao, W. Xia. Chem.Commun. 53 (2017) 8533-8536.

7. J. Romero-Parra, J. Mella-Raipan, V. Palmieri, M. Allara, M.J. Torres, H. Pessoa-Mahana, P. Iturriaga-Vasquez, R. Escobar, M. Faúndez, V. Di Marzo, C.D. Pessoa-Mahana. Eur. J. Org. Chem. (2016) 17-35.

8. P. P. Kulkarni,·A. J. Kadam, R. B. Mane, Uday V. Desai and P. P. Wadgaonkar. J. Chem. Research (S). (1999) 394-395.

9. V.I. Bonano, J.K.U. Yokoyama- Yasunaka, D. C. Miguel, S.A. Jones, J.A. Dodge, S.R.B. Uliana. Chem Biol Drug Des 2014; 83: 289-296.
